# Detection of clade 2.3.4.4 highly pathogenic avian influenza H5 viruses in healthy wild birds in the Hadeji‐Nguru wetland, Nigeria 2022

**DOI:** 10.1111/irv.13254

**Published:** 2024-02-03

**Authors:** Kayode Olawuyi, Olukayode Orole, Clement Meseko, Isabella Monne, Ismaila Shittu, Zecchin Bianca, Alice Fusaro, Bitrus Inuwa, Ruth Akintola, Josiah Ibrahim, Maryam Muhammad

**Affiliations:** ^1^ National Veterinary Research Institute Vom Nigeria; ^2^ Department of Microbiology Federal University of Lafia Lafia Nigeria; ^3^ Istituto Zooprofilattico Sperimentale delle Venezie Padova Italy; ^4^ AP Leventis Ornithological Research Institute Jos Nigeria

**Keywords:** AIV, H5N1, Hadejia‐Nguru, HPAI, migratory wild birds

## Abstract

**Background:**

The introduction of multiple avian influenza virus (AIV) subtypes into Nigeria has resulted in several poultry outbreaks purportedly linked to trade and wild birds. The role of wild birds in perpetuating AIV in Nigeria was, therefore, elucidated.

**Methods:**

A cross‐sectional study was conducted among wild aquatic bird species at the Hadejia‐Nguru wetlands in Northeastern Nigeria between March and April 2022. A total of 452 swabs (226 cloacae and 226 oropharyngeal) were collected using a mist net to capture the birds. These samples were tested by RT‐qPCR, followed by sequencing.

**Results:**

Highly pathogenic AIV of the H5N1 subtype was identified in clinically healthy wild bird species, namely, African jacana, ruff, spur‐winged goose, squared‐tailed nightjar, white‐faced whistling ducks, and white stork. A prevalence of 11.1% (25/226) was recorded. Phylogenetic analysis of the complete HA gene segment indicated the presence of clade 2.3.4.4b. However, these H5N1 viruses characterized from these wild birds cluster separately from the H5N1 viruses characterized in Nigerian poultry since early 2021. Specifically, the viruses form two distinct genetic groups both linked with the Eurasian H5N1 gene pool but likely resulting from two distinct introductions of the virus in the region. Whole‐genome characterization of the viruses reveals the presence of mammalian adaptive marker E627K in two Afro‐tropical resident aquatic ducks. This has zoonotic potential.

**Conclusion:**

Our findings highlight the key role of surveillance in wild birds to monitor the diversity of viruses in this area, provide the foundations of epidemiological understanding, and facilitate risk assessment.

## 1 INTRODUCTION

Wetlands are terrestrial or semi‐terrestrial ecosystems characterized by low drainage, slow waters, or seldom standing water bodies filled with soil. Wetlands are very important and valuable components of the ecosystem. They serve as a habitat for man and animals, a source of food, shelter, and other ecosystem functions. For many faunas, wetlands are the life‐enhancing systems of the environment; they consist of direct and indirect components. Ecologically, wetlands are very important habitats for birds as they utilize the resources for feeding, breeding, foraging, and resting.^1^ The seasonal long‐distance migration of wild birds from Europe to Africa along the Western Siberian‐Mediterranean‐West Africa flyway during winter and the subsequent stopover at the wetlands plays an important role in the introduction of highly pathogenic avian influenza virus (HPAI) to the African region.^2^, ^3^, ^4^ In Nigeria, several coastal and inland wetlands serve as congregation sites for both migratory and resident waterfowl. The Hadejia‐Nguru wetland is the largest in Northeastern Nigeria with an estimated coverage area of 3500 km^2^.^5^ These wetlands serve as seasonal shelters and migratory routes for approximately 5.4 million wild aquatic birds, making seasonal movements between the temperate zone and the tropics during winter.^6^ Over 80 wild bird species have been caught in this location during bird counts and avian influenza surveillance.^7^ The interaction between the migratory birds and the indigenous resident birds during stop‐overs at the wetlands provides a veritable platform for the exchange of influenza A viruses and their genetic materials.

Nigeria within the sub‐Saharan agroecological region has witnessed several introductions of avian influenza virus (AIV) since 2006, and there is strong evidence of the role of wild birds in the ecology and epidemiology of HPAI.^8^, ^9^, ^10^, ^11^, ^12^, ^13^ The Hadejia‐Nguru wetlands are located about 220 km from the first HPAI H5N1 outbreaks reported in Kano State in January 2015 and could be a source of infection and maintenance of the virus.^2^, ^8^, ^14^ Although there is low transmissibility of HPAIV from birds to humans and high fatality has been reported in humans in previous epidemics, the potential for a mutant strain of HPAIV to become pandemic through spillover from infected migratory birds poses a threat to public health.^15^

Several phylogenetically distinct sub‐lineages of the HPAIV have been reported in wild migratory birds.^16^ In Nigeria, the HPAIV H5N1 belonging to the genetic clade 2.3.2.1c was reported in 2015 from samples collected from backyard poultry as well as live bird market (LBM).^17^, ^18^ Before that, however, outbreaks of HPAIV subtype H5N1 with genetic clades 2.2 and 2.2.1 had occurred in the country in 2006–2008.^19^, ^20^ These outbreaks have had a devastating impact on the poultry industry as well as international trade in poultry and poultry products.^21^ Owing to the antigenic variability and the segmented nature of the influenza virus genome, several reassortant strains and subtypes of the AIVs have been reported in Nigerian poultry.^22^ These include the H5N1,^17^, ^18^, ^19^, ^21^ H5N2,^23^ H5N8,^12^, ^24^ H5N6,^25^ and H9N2.^26^ Previously, Gaidet et al.^27^ had reported the detection of the H5N2 subtype of the AIV with highly pathogenic characteristics in apparently healthy wild waterfowl species sampled in the Hadejia‐Nguru wetlands in Nigeria. It is speculated that AIV is not yet endemic in Nigeria, but rather that there are repeated introductions by migratory wild birds (Meseko pers. com). For over a decade, no active AIV surveillance has been implemented in the wild bird population hosted by one of the most relevant wetlands of the Western African region, Hadejia‐Nguru. This has negatively impacted the ability to design strategies to recognize and mitigate potential AI threats for the region coming from wildlife populations. Therefore, the objective of the surveillance effort described herein was to identify the circulating AIV subtypes in the wild birds in the Hadejia‐Nguru wetlands and to gain deeper insight into the ecology of the virus. Making use of the unique collection of samples from this important wild bird area, we provide new insights into the epidemiology of the disease in wild migratory and resident birds in Northeastern Nigeria.

## 2 MATERIALS AND METHODS

### 2.1 Description of study location

**FIGURE 1:**
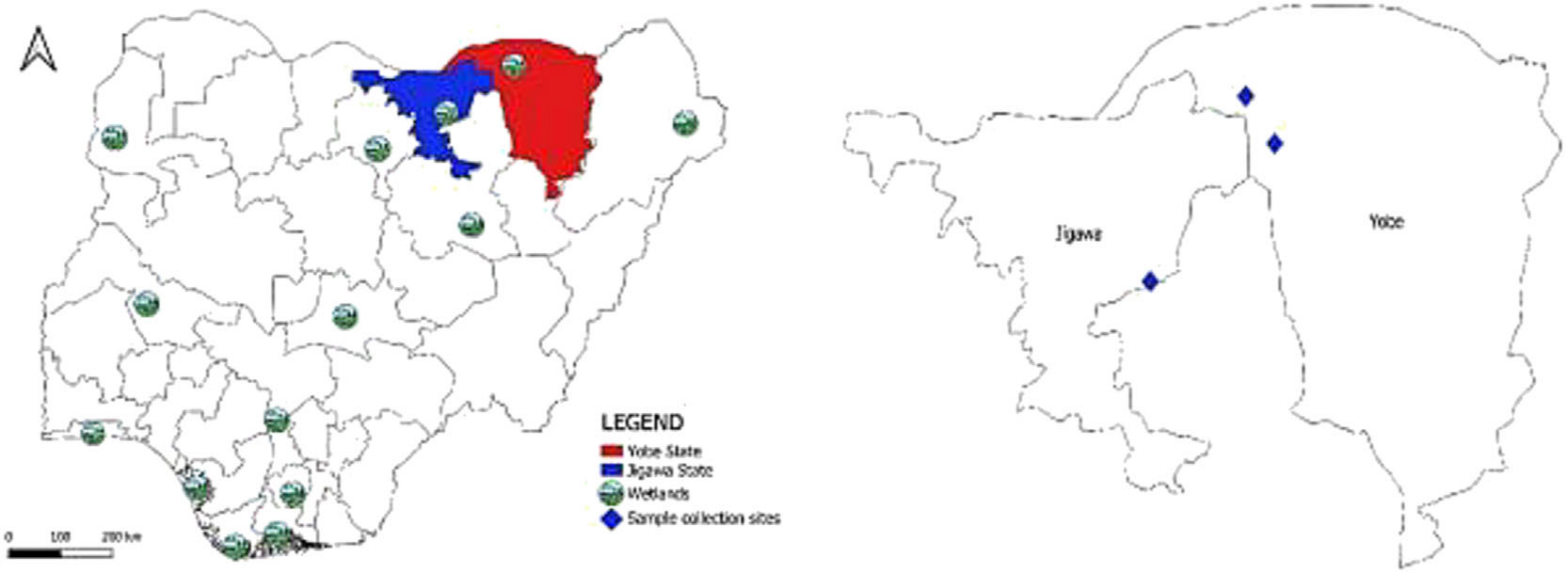
Map of Nigeria showing wetlands, Jigawa and Yobe states, and sample collection sites.

The Hadejia‐Nguru wetlands are a wide expanse of floodplain wetlands situated in northeast Nigeria, in the Sudan‐Sahelian region, which is the zone between the Sudanian Savanna in the south and the Sahel in the North. It is one of Nigeria's major wetlands, covering an area of about 3500 km^2^ with an altitude of 152–305 m above sea level and a depth of about 1.6–1.7 m. The annual rainfall ranges between 200 and 600 mm, from late May to September. The mean minimum temperature ranges from 12°C in December to January, reaching a maximum of 40°C in April.^5^, ^28^ These wetlands are drained by two major rivers, the Hadejia and the Jama'are, which flow and converge into Lake Chad (Figure 1). The wetlands enclose Kano, Jigawa, Yobe, Gombe, and Bauchi States. It is an ecologically and economically rich habitat for the biodiversity of various fauna and flora. The area is a major tourist location for the Palaearctic and Afrotropical migrant water birds. Large numbers of diverse migratory species mix with resident wild birds and domestic waterfowl.^7^

### 2.2 Study design, sample collection, and processing

**FIGURE 2:**
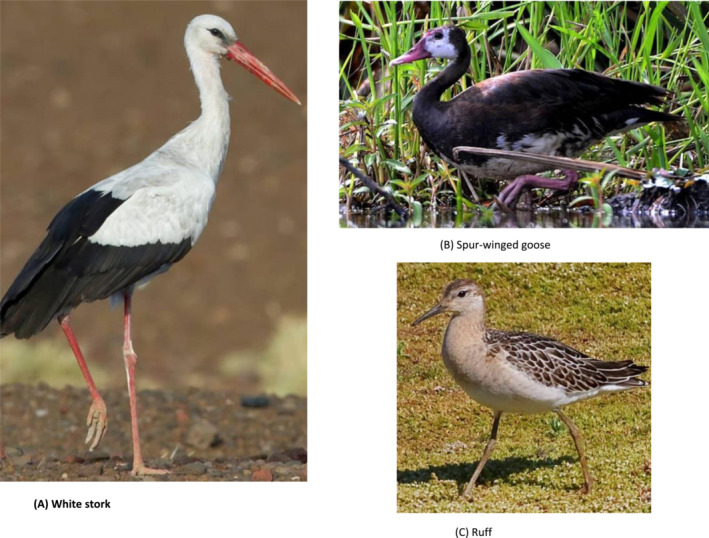
(A) White stork, (B) spur‐winged goose, and (C) ruff.

A cross‐sectional study design was adopted for this survey. Wild birds were sampled opportunistically using a mist net and bird‐call sound playbacks. Birds that get entangled are quickly removed to avoid injury, and a swab sample is collected. The birds are released back into their natural habitat as a key conservation element. All the birds sampled were apparently healthy (Figure 2A–C).

Bird species were identified with the aid of bird atlas. Tracheal and cloacae swabs were collected from migratory and resident aquatic wild birds between March and April 2022. These samples were collected into 1‐mL glycerol‐based virus transport medium and transported on ice to the National Veterinary Research Institute, Vom Regional Support Laboratory for Animal Influenza and Newcastle Disease, where the samples were analyzed using a standardized protocol as previously described.^25^, ^29^

### 2.3 Molecular detection of avian influenza virus

Using the QIAamp Viral RNA Mini Kit (Qiagen, Hilden, Germany), total RNA extraction was conducted based on the manufacturer's instructions. To detect the influenza A virus matrix gene (M‐gene),^30^ the RNA extracts were tested by the quantitative reverse‐transcriptase polymerase chain reaction (RT‐qPCR) using the QuanTitect® Multiplex RT‐PCR kit (Qiagen®, Hilden Germany) in the Rotor‐Gene Q thermocycler (Qiagen, Hilden, Germany). The M‐gene‐positive samples were subsequently sub‐typed for the hemagglutinin gene (HA) using an established H5N1 duplex protocol.^31^ The QuantiTect® Multiplex RT‐PCR kit was used (Qiagen, Hilden Germany). Briefly, the master mix reagents included a 5× PCR buffer, H5 probe mix (10 μm), N1 probe mix (10 μm), dNTP (10 μm), Mgcl2 (25 μm), Rnasin (40 μm), and enzyme mix (1 μm). The RNA template (5 μm) was added to the mix and was amplified using the following cycling conditions: 50°C for 30 min, 95°C for 15 min, 95°C for 30 s, 56°C for 30 s, and 72°C for 10 s with 45 cycles/repeats. The fluorescence for H5 was acquired at the HEX, whereas the N1 was at the FAM. These represent the targeted probes/dye. The Ct value less than or equal to 35 for each sample was considered positive for H5 and N1 genes respectively.

### 2.4 Virus isolation

All samples that were positive for M‐gene were selected for virus isolation according to a standard method.^29^ The samples were inoculated into 9‐ to 11‐day chicken embryonated eggs obtained from a specific pathogen‐free (SPF) flock. The inoculated eggs were incubated at 37°C and candled at 24‐h intervals for 5 days. Eggs with dead embryos within the first 24 h post‐inoculation were discarded as non‐specific mortalities. Subsequently, eggs with dead embryos were set aside in a refrigerator at +4°C. The resultant allantoic fluid containing the virus was harvested and tested for the presence of hemagglutinating activities serologically.^29^

### 2.5 Genome sequencing and phylogenetic analysis

Seventeen HPAI H5 positive clinical specimens were sent to the European, WOAH, and FAO reference laboratory for avian influenza (AI) in Italy (Istituto Zooprofilattico Sperimentale delle Venezie) for sequencing and genetic data analysis. A target RT‐PCR approach was used to amplify influenza A virus whole genomes as previously described.^12^ Sequencing libraries were obtained using the Illumina DNA prep kit (Illumina, San Diego, CA, USA) and sequenced on MiSeq instrument using a 2 × 300 bp PE mode. Raw sequencing reads produced by MiSeq instrument were cleaned with Trimmomatic v0.32 with minimum quality 20. Illumina DNA prep adapter sequences were clipped from reads using scythe v0.991 (https://github.com/vsbuffalo/scythe), and amplification primers were removed using sickle v1.33 (https://github.com/najoshi/sickle). Reads shorter than 80 bp or unpaired were discarded. The cleaned reads were aligned against a reference genome using the MEM algorithm from BWA v0.7.12‐r1039. Picard tools v2.1.0 (http://broadinstitute.github.io/picard/) and GATK v3.530‐32^32^ were used to improve alignment quality, correct potential errors, and recalibrate base quality score. LoFreq v2.1.2.33^33^ was used to call single nucleotide polymorphisms that were reported in a vcf (Variant Call Format) file. The generated vcf file was then used to produce the consensus sequences using an in‐house script. Briefly, this script calls the base for each position with a coverage >10×, considering all the polymorphisms with a frequency higher than 25%. “N” is assigned to all positions with a coverage lower than 10 reads.

Consensus sequences were aligned using MAFFT v7 online server (https://mafft.cbrc.jp/alignment/server/)^34^ and compared with sequences of most related virus strains available in GISAID. Maximum likelihood phylogenetic trees of each gene segment were obtained by using IQTREE v1.6.6 (https://github.com/iqtree/iqtree1), and its robustness was determined through an ultrafast bootstrap resampling analysis of 1000 replications.^35^ Phylogenetic trees were visualized using Fig Tree v1.4.4 software (http://tree.bio.ed.ac.uk/software/figtree/). Virus sequences were deposited in the GISAID EpiFlu Database (http://platform.gisaid.org) with the accession numbers EPI_ISL_18462524‐18462541 (Tables S1 and S2).

## 3 RESULTS

In all, a total of 452 swab samples from 226 birds were collected (226 tracheal and 226 cloaca swabs) from 30 different bird species. Of these birds, 75% (170/226) were adults and 25% (56/226) were juveniles. The resident aquatic birds were 70.1% (158/226), 22.2% (50/226) were Palearctic migratory birds, and 8% (18/226) were intra‐African migrants. The detailed distribution of the bird species, migrant type, molecular analysis results, and prevalence of AIV from Baturia, Dagona waterfowl sanctuary, and Nguru Lake of the Hadejia‐Nguru wetlands respectively are presented in Table 1. A total of 129 wild birds were sampled from the Dagona waterfowl sanctuary. AIV subtype H5N1 was detected from apparently healthy African jacana (*Actophilornis africanus*) (1.6%), white‐faced whistling ducks (WFWD) (*Dendrocygna viduata*) (3.1%), and ruff (*Calidris pugnax*) (2.3%) from this location. A location‐specific prevalence of 7% for AIV was obtained (Table 1). AIV was also detected from 5/44 of the captured wild birds from the Baturia section of the Hadejia‐Nguru wetland with a prevalence of 11.4% among the WFWD, and at Nguru Lake, 53 wild birds were sampled. AIV subtype H5N1 was detected from spur‐winged goose (*Plectroptrus gambensis*) (3.8%), squared‐tailed night jar (*Caprimulgus fosii*) (5.7%), and white stork (*Ciconia Ciconia*) (11.3%) from this location. The overall prevalence of AIV from the Hadejia‐Nguru wetlands was 11.1% (25/226) (Table 2).

**TABLE 1:** Distribution of bird species, migrant type, molecular analysis results, and the prevalence of AIV at Hadejia‐Nguru wetlands, Northeast Nigeria.

Common name	Species	Location	No. sampled		Migrant type	Mgene +Ve/No sampled	Prevalence (%)	H5N1 + Ve/Mgene +Ve
			CS	OS				
							*N* = 129, 44, & 53	
African jacana	*Actophilornis africanus*	Baturia	9	9	Resident	0/9		
		Dagona	49	49		2/49	1.6	2/2
		Nguru	4	4		0/4		
African Pygmy Goose	*Nettapus auritus*	Baturia	0	0	Intra‐African migrant	0/0		
		Dagona	10	10		0/10		
		Nguru	0	0		0/0		
WFWD	*Dendrocygna viduata*	Baturia	14	14	Resident	5/14	11.4	5/5
		Dagona	16	16		4/16	3.1	4/4
		Nguru	0	0		0/0		
Spur‐winged goose	*Plectropterus gambensis*	Baturia	2	2	Resident	0/2		
		Dagona	4	4		0/4		
		Nguru	13	13		2/13	3.8	2/2
Squacco heron	*Ardeola ralloides*	Baturia	1	1	Palearctic	0/1		
		Dagona	7	7		0/7		
		Nguru	0	0				
Square‐tailed night jar		Baturia	0	0	Resident			
		Dagona	0	0				
		Nguru	11	11		3/11	5.7	3/3
Ruff	*Calidris pugnax*	Baturia	2	2	Palearctic	0/2		
		Dagona	14	14		3/14	2.3	3/3
		Nguru	1	1		0/1		
Cattle egret	*Bubulcus ibis*	Baturia	5	5	Resident	0/5		
		Dagona	7	7		0/7		
		Nguru	0	0				
Pied kingfisher	*Ceryle rudis*	Dagona	6	6	Resident	0/6		
Black Headed Heron	*Ardea melanocephala*	Dagona	7	7	Resident	0/7		
Common snipe	*Gallinago gallinago*	Dagona	2	2	Paleartic	0/2		
Laughing dove	*Spilopelia senegalensis*	Dagona	2	2	Resident	0/2		
Red‐eyed dove	*Streptopelia semitorquata*	Dagona	2	2	Resident	0/2		
Fulvous Whistling Duck.	*Dendrocygna bicolor*	Dagona	2	2	Resident	0/2		
		Baturiya	1	1		0/1		
Glossy ibis	*Plegadis falcinellus*	Dagona	1	1	Paleartic	0/1		
Grey heron	*Ardea cinerea*	Dagona	1	1	Paleartic	0/1		
African. Open Billed Stork	*Anastomus lamelligerus*	Dagona	1	1	Intra‐African migrant	0/1		
Knob billed duck	*Sarkidiornis melanotos*	Baturiya	2	2	Intra‐African migrant	0/2		
Egyptian goose	*Alopochen aegyptiaca*	Baturiya	1	1	Intra‐African migrant	0/1		
Hadada ibis	*Bostrychia hagedash*	Baturiya	1	1		0/1		
African spoonbill	*Platalea alba*	Baturiya	1	1	Intra‐African migrant	0/1		
Wigeons	Mareca penelope	Baturiya	1	1	Paleartic	0/1		
African barn owl	*Tyto capensis*	Baturiya	2	2	Intra‐African migrant	0/2		
Red‐eyed dove	*Streptopelia semitorquata*	Baturiya	1	1	Resident	0/1		
Whitestork	*Ciconia ciconia*	Nguru	17	17	Paleartic	6/17	11.3	6/6
Purple swamphen	*Porphyrio porphyrio*	Nguru	2	2	Resident			
Little egret	*Egretta garzetta*	Nguru	1	1	Intra‐African migrant	0/1		
Little bitten	*Ixobrychus minutus*	Nguru	1	1	Pacleartic	0/1		
Black kite	*Milvus migrans*	Nguru	1	1	Intra‐African migrant	0/1		
Intermediate egret	*Ardea intermedia*	Nguru	1	1	Resident	0/1		

*Note*: *N* = 129, Dagona water fowl sanctuary; *N* = 44, Baturia; *N* = 53, Nguru lake.

Abbreviations: CS, cloaca swab; OS, oropharyngeal swab.

**TABLE 2:** Overall prevalence of AIV from wild birds in Hadejia‐Nguru wetland, Northeastern Nigeria 2022.

Location	Common name	Species	No. sampled	No. Mgene +Ve	No. H5N1 + Ve	Prevalence (%) *N* = 226	Sequence analysis
**DWS**	African jacana	*Actophilornis africanus*	49	2	2	0.9	2 HPAI
	Ruff	*Calidris pugnax*	14	3	3	1.3	2 HPAI
	WFWD	*Dendrocygna viduata*	16	4	4	1.8	2 HPAI
**BTR**	WFWD	*Dendrocygna viduata*	14	5	5	2.2	3 HPAI
**NL**	Squared‐tailed night jar	*Caprimulgus fossii*	11	3	3	1.3	2 HPAI
	Spur‐winged goose	*Plectropterus gambensis*	13	2	2	0.9	2 HPAI
	White stork	*Ciconia Ciconia*	17	6	6	2.7	4 HPAI
		**Other species**	92	0	0	0	

*Note*: No. of HPAI confirmed by genome sequencing. Other species = samples collected but negative for AIV.

Abbreviations: BTR, Baturia; DWS, Dagona waterfowl sanctuary; NL, Nguru lake.

Virus isolation in embryonating SPF chicken eggs was attempted for all M gene‐positive samples according to standard procedures.^29^ A total of 25 virus isolates were obtained and stored appropriately.

## 4 PHYLOGENETIC AND GENOMIC ANALYSIS

**FIGURE 3:**
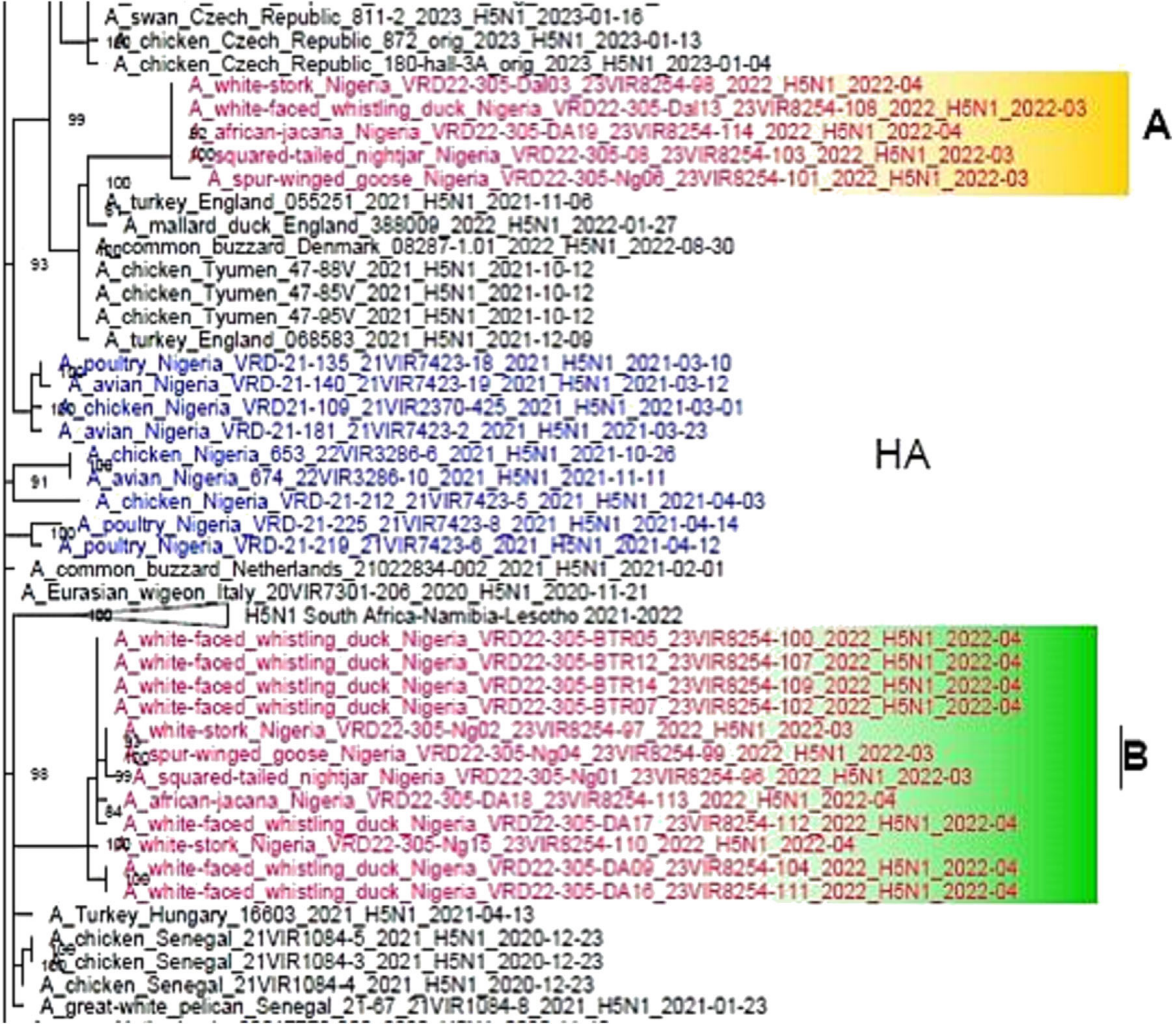
Maximum likelihood phylogenetic tree of the HA gene segment obtained in IQtree v1.6.6. The viruses analyzed in this work are marked in pink and cluster within group A (yellow box) and group B (green box). The viruses previously detected in Nigeria are shown in blue. Ultrafast bootstrap values higher than 80 are shown next to the nodes.

Whole‐genome sequences were obtained for 17 H5N1 viruses identified in wild birds in Nigerian wetlands. Analysis of the hemagglutinin (HA) gene segment confirms the presence of a cleavage site motif typical of the HPAI viruses (KRRKR*GLF) and shows that the HPAI H5N1 viruses from wild birds (Figure 3) belong to clade 2.3.4.4b but cluster separately from the H5N1 viruses, which have been circulating in the Nigerian poultry population since early 2021.^36^ Specifically, the viruses identified in wild birds form two distinct groups in all the phylogenetic trees provisionally named here A and B groups (Figure 2 and Figures S1–S7). Most of the viruses detected in wild birds in the Hadejia‐Nguru wetlands cluster together in group B with viruses identified in wild and domestic birds in Senegal in December 2020–January 2021 and with viruses detected in Europe, suggesting that these viruses have been introduced concurrently into the West African region from Europe during the winter season 2020–2021. Five viruses fall within group A and cluster with H5N1 viruses, which have been circulating in Europe during the 2021–2022 epidemic wave, suggesting that it may represent a more recent introduction in Nigeria (Figure 2). All the 17 viruses characterized are descendants of the H5N1 A/Eurasian_Wigeon/Netherlands/1/2020‐like genotype, which most likely emerged in Europe in 2020. Molecular analysis of the consensus sequences of the HPAI H5N1 viruses from wild birds revealed the presence of several mutations that are likely associated with an increased zoonotic potential (Table 3). The mutations identified in the HA protein, which have proved to increase in vitro binding to human‐type receptors (i.e., S133A, S154N, T156A, S107R, T108I, K218Q, and S223R) and the NS1 changes, described to increase replication in mammalian cells, have been identified in the majority of the A(H5N1) viruses circulating in Europe since October 2020; same applies to the mutations identified in the PB1 and PA genes, which have been already reported in 2.3.4.4b viruses detected in Europe.^37^ It is, therefore, likely that these mutations have been acquired elsewhere before being introduced in Nigerian wildlife.

**TABLE 3:** Amino acid markers associated with specific phenotypic effects found in the genome of HPAI H5N1 viruses sequenced in the current study.

Protein	Mutation	Effect	Samples
**PB2**	K389R	Increased polymerase activity and replication in mammalian cell line	All H5N1 Nigerian viruses analyzed
	**E627K**	**Enhanced polymerase activity, and increased virulence in mice, contribute to airborne pathogenicity of IAVs in ferrets and contact transmission in guinea pigs.**	**A/white‐faced_whistling_duck/Nigeria/ DA16_23VIR8254‐111/2022, A/white‐faced_whistling_duck/Nigeria/DA09_23VIR8254‐104/2022**
	L89V, G309D	Increased polymerase activity in mammalian cell line and increased virulence in mice	All H5N1 Nigerian viruses analyzed
**HA**	S133A	Increased pseudovirus binding to α2–6	All H5N1 Nigerian viruses analyzed
	S154N	Increased virus binding to α2–6	All H5N1 Nigerian viruses analyzed
	T156A	Increased virus binding to α2–6, increased transmission in guinea pigs	All H5N1 Nigerian viruses analyzed
	S107R, T108I	Increased virulence in chickens and mice, increased pH of fusion	All H5N1 Nigerian viruses analyzed
	K218Q, S223R	Increased virus binding to α2–3 and α2–6	All H5N1 Nigerian viruses analyzed
**NS1**	P42S	Increased virulence and decreased antiviral response in mice	All H5N1 Nigerian viruses analyzed
	I106M	Increased viral replication in mammalian cells virulence in mice	All H5N1 Nigerian viruses analyzed
	C138F	Increased replication in mammalian cells, decreased interferon response	All H5N1 Nigerian viruses analyzed
	V149A	Increased virulence and decreased interferon response in chickens	All H5N1 Nigerian viruses analyzed
	L103F, I106M	Increased virulence in mice	All H5N1 Nigerian viruses analyzed
	K55E, K66E, C138F	Enhanced replication in mammalian cells, decreased IF response	All H5N1 Nigerian viruses analyzed
**PB1**	D3V	Increased polymerase activity and viral replication in avian and mammalian cell lines	All H5N1 Nigerian viruses analyzed
	D622G	Increased polymerase activity and virulence in mice	All H5N1 Nigerian viruses analyzed
**PB1‐F2**	N66S	Enhanced replication, virulence, and antiviral response in mice	All H5N1 Nigerian viruses analyzed
**PA**	S37A	Increased polymerase activity in mammalian cell line	All H5N1 Nigerian viruses analyzed
	P190S	Decreased virulence in mice	All H5N1 Nigerian viruses analyzed
	N383D	Increased polymerase activity in mammalian and avian cell lines	All H5N1 Nigerian viruses analyzed
	Q400P	Decreased virulence in mice	A/white‐stork/Nigeria/Dal03_23VIR8254‐98/2022_H5N1_2022‐04, A/spur‐winged_goose/Nigeria/ ‐Ng06_23VIR8254‐101/2022_H5N1_2022‐03, A/squared‐tailed_nightjar/Nigeria/08_23VIR8254‐103/2022_H5N1_2022‐03, A/white‐faced_whistling_duck/Nigeria/Dal13_23VIR8254‐108/2022_H5N1_2022‐03, A/african‐jacana/Nigeria/DA19_23VIR8254‐114/2022_H5N1_2022‐04
	N409S	Increased polymerase activity and replication in mammalian cell line	All H5N1 Nigerian viruses analyzed
**NP**	M105V	Increased virulence in chickens	All H5N1 Nigerian analyzed
	A184K	Increased replication in avian cells and virulence in chickens, enhanced IFN response	All H5N1 Nigerian analyzed
**M1**	N30D	Increased virulence in mice	All H5N1 Nigerian analyzed
	I43M	Increased virulence in mice, chickens, and ducks	All H5N1 Nigerian analyzed
	T215A	Increased virulence in mice	All H5N1 Nigerian viruses analyzed

Two viruses (A/white‐faced_whistling_duck/Nigeria/DA16_23VIR8254‐111/2022, A/white‐faced_whistling_duck/Nigeria/DA09_23VIR8254‐104/2022) present the E627K mutation in the PB2 gene, a well‐known molecular marker of mammalian adaptation. The viruses presenting this adaptive mutation were both detected in Afro‐tropical resident aquatic ducks sampled in the same location (the Dagonal waterfowl sanctuary) in April 2022 suggesting that the two affected animals may have been exposed to the same pathogen source.

## 5 DISCUSSION

In this study, 30 different wild bird species were identified; these include, among others, afro‐tropical ducks such as white‐faced whistling ducks, spur‐winged geese, and fulvous whistling ducks. Also found were waders such as ruff, common snip, herons such as grey‐head heron, common squacco heron, and black‐headed heron (Table 1). This finding corroborates previous reports of the species of wild birds found in the Hadejia‐Nguru wetland.^7^, ^11^, ^38^ African jacana (*Actophilornis africanus*), ruff (*Calidris pugnax*), spur‐winged goose (*Plectropterus gambensis*), squared‐tailed nightjar (*Caprimulgus fossii*), white stork (*Ciconia Ciconia*), and white‐faced whistling ducks (WFWD) (*Dendrocygna viduata*) are Afrotropical and Palearctic wild bird species found to harbor HPAI of the H5N1 subtype in the study. Spur‐winged goose and the WFWD (Anatidae species) were previously reported to harbor the low pathogenic AIVs.^39^, ^40^ These ducks exhibit similar foraging behavior and share the same feeding and roosting habitat. Their gregarious nature and foraging in shallow waters expose them to AIV infection. The detection of HPAI from these species within the region partly agrees with the report of Gaidet et al.,^27^ who earlier detected the H5N2 subtype of the AIV with a highly pathogenic characteristic in apparently healthy wild waterfowl from the Hadejia‐Nguru wetland. These H5N2 viruses were genetically related to the H5 low pathogenic strains found in Eurasian wild and domestic ducks. In our study, H5N1 virus belonging to clade 2.3.4.4b was detected over a decade later in the same location and presented even in this case the highest similarities with Eurasian viruses confirming these wetlands as an area at risk of introduction of viruses from Eurasia.

The African jacana species, from which AIV of the H5N1 subtype was also detected, are waders common in freshwater wetlands in sub‐Saharan Africa. These intra‐African migrants walk on floating vegetation to feed on aquatic insects. The detection of AIV in this species of wild birds disagrees with the studies by Poen et al.^41^ who reported that no AIV was detected in the African jacana species screened in South Africa. HPAI was also detected in the cloaca and oropharyngeal samples from white stork species (*Ciconia Ciconia*). These medium to large wading birds travel long distances over the Weser River in Germany, fly southward through France and Spain, and move over the Strait of Gibraltar to West Africa. The birds arrive in the West African subregion in November and return to Europe around April–May of the following year.^42^ These birds feed on crickets, earthworms, beetles, amphibians, and other insects, which are abundant in the Hadejia‐Nguru wetlands, which is a wintering site for the Eurasian migrants. The detection of the AIV H5N1 subtype belonging to clade 2.2.3.4b in white stork species is consistent with the report by Abolnik et al.^4^ who also detected AIV clade 2.3.4.4b among moribund white stork in South Africa. Infections in this species have been repeatability reported also in Europe suggesting that white stork could spread the virus along its migratory route.^37^

Ruff (*Calidris pugnax*) was another migratory bird species found infected by H5N1 in this study. This is a medium‐sized shorebird that breeds mostly across northern Europe and winter in Africa. These gregarious bird species travel over 14,000 km annually in large flocks from Europe to the wintering ground in West Africa with few stopovers.^43^ The detection of HPAI H5N1 in this healthy bird indicates that birds belonging to this species may have been also involved in the dissemination of the virus from its breeding site in Europe.

Through phylogenetic analysis, we were able to demonstrate the existence of a genetic relationship between the viruses identified in our study and the ones previously detected in the Eurasian territories. In particular, we identified the introduction of at least two distinct genetic clusters of the H5N1 virus (groups A and B) in the wild bird population of Hadejia‐Nguru wetlands. Specifically, 12 viruses isolated from five different wild bird species cluster together (group B) and with viruses identified in wild and domestic birds in Europe (2020–2021) and in Western (2020–2022) and Southern African regions (2021–2022).^44^ The chronological order of the events and the phylogenies obtained for most of the genes suggest that group B viruses may have been introduced concurrently in multiple destinations in the West African region from Europe during the winter season of 2020–2021 and have been then able to persist and further spread in the African territories. The five viruses clustering within group A, identified from both Afrotropical species and Palearctic species, show the highest identity with H5N1 viruses, which have been circulating in Europe more recently starting from 2021. This may suggest that viruses belonging to the A group have been introduced independently and more recently in Nigeria than group B viruses. Surprisingly, the molecular analysis revealed the presence of the mammalian adaptive markers E627K in two viruses identified in Afro‐tropical resident aquatic ducks from Dagonal waterfowl sanctuary. Data from the European region indicate that these mutations have rarely been identified in the HPAI A(H5) viruses of clade 2.3.4.4b collected in birds in Europe since October 2020, whereas about 47% of the H5N1 viruses characterized from infected mammals contain at least one of the adaptive markers associated with increased virulence and replication in mammals in the PB2 protein (E627K, D701N, T271A, or K526R).^37^, ^45^ This observation suggests that these mutations with potential public health implications likely emerge upon transmission to mammals. So far, no 2.3.4.4b H5N1 infected mammals have been reported in West Africa, but these findings highlight the risk that undetected spillover events may have been occurring also in this region. Furthermore, limited surveillance of AIV in mammals in Nigeria may cause missed opportunities for early detection and early warning of emerging novel influenza viruses with this or another type of important mutation. In a previous report on AIV in mammals in Nigeria,^11^ the exposure of domestic pigs to the H5N1 virus was described, which highlights the potential public health and pandemic risk by unreported but circulating AIV with mammalian adaptations. Therefore, the need for expansion and enhancement of surveillance in poultry and mammals cannot be over‐emphasized due to interspecies transmission and the occurrence of mutations of note.

Although no mortality events were observed in the wild birds sampled in the current study, the virus was actively replicating in the respiratory and digestive tract as evidenced in the positive results from oropharyngeal and cloaca swabs respectively. The detection of HPAI virus subtype H5N1 of clade 2.3.4.4b in apparently healthy wild birds in Nigeria confirms previous reports even in Nigeria^46^ when two isolates of HPAI were recovered from the tracheal swab samples from apparently healthy ducks. There are other reports from Russia,^47^ China,^48^ South Korea,^49^ and Italy.^50^ However, it is worth noting that, since early 2023, multiple mass mortality events in wild birds have been reported in other parts of West Africa including the Gambia, Senegal, and Guinea Bissau.^44^, ^51^ Therefore, considering the dynamic situation of HPAI, it is pertinent to consider the conservation risk for wildlife inhabiting the Nigerian wetlands and the possibility of cross‐sectoral workings to include the Ministry of Environment in a one‐health approach strategy to develop and implement risk mitigation plans to protect the region.

The maintenance of this virus in the wild bird population could partly explain the sustained outbreaks of the HPAI H5N1 subtype in poultry across the country. These wild birds are sometimes captured by hunters, kept within the village, and taken to the LBM for sale. These birds eventually interact with poultry, hence the recurring outbreaks. This assertion is partly supported by reports from these authors^11^, ^52^, ^53^ who opined that the migration of these birds enhances the geographical spread of the influenza virus and may bridge infections to resident aquatic birds and gallinaceous poultry. It is also worthy of note that the Hadejia‐Nguru wetland location is about 220 km northeast of Kano City where the re‐emergence of H5N1 was previously reported in January 2015 from backyard poultry.^17^

Ruff and white storks are important Palearctic birds sampled in this study and were positive for the HPAI H5N1. This implies that as these birds migrate from one continent to another, they can transmit the virus to the resident aquatic birds, which can then further disseminate the virus across the region. In a recent report on the diversity and evolution of highly pathogenic avian influenza in Nigeria,^36^ 2023 confirm the detection of H5N1, H5N2, and H5N8 of the clade 2.3.4.4b in commercial poultry farms across different states in Nigeria. Comparison of the H5Nx viruses of the clade 2.3.4.4b viruses detected and characterized in poultry in this study^36^ revealed that these poultry viruses are genetically distinct from the ones identified in Hadejia Nguru wetland. The adduced reasons for this genetic divergence can include the inadequate/limited surveillance activities for HPAI in wild birds in wetlands across the country. This has resulted in the paucity of data on the subtypes of HPAI viruses in circulation per time in these wetlands. The Hadjia‐Nguru wetland though is an important internationally recognized wetland, and there are other wetlands through which wild birds can access the country to introduce the virus, which includes the Apoi Creek in Bayelsa, Forge Islands in Kebbi, Oguta Lake in Imo, Pandam and Wase lakes in Nassarawa‐Plateau, and the upper Orashi forests in Rivers state.^54^, ^55^ We, therefore, recommend sustained risk‐based surveillance for AIV in wild birds for early detection, early warning alert for the poultry industry, disease mitigation, and control.

### 5.1 Study limitation

This study was not able to trace the onward trajectory destinations of the migratory birds because the resources and technology of fitting satellite telemetry were limited. We recommend close monitoring of migratory and resident wild birds not only in Hadejia‐Nguru but other wetlands across the country where the independent introduction of AIV has been reported in the past. A system of surveillance using local waterfowl as a sentinel to study the type, time, and frequency of introduction of AIV from wild to domestic waterfowl is recommended.

## AUTHOR CONTRIBUTIONS

**Kayode Abraham Olawuyi:** Conceptualization; data curation; formal analysis; investigation; methodology; project administration; writing—original draft; writing—review and editing. **Olukayode Olugbenga Orole:** Conceptualization; funding acquisition; resources; supervision; validation; writing—review and editing. **Clement Adebajo Meseko:** Conceptualization; funding acquisition; resources; supervision; validation; writing— review and editing. **Isabella Monne:** Conceptualization; funding acquisition; resources; software; supervision; validation; writing—review and editing. **Ismaila Shittu:** Conceptualization; funding acquisition; resources; supervision; validation; writing—review and editing. **Zecchin Bianca:** Writing—review and editing. **Alice Fusaro:** Writing—review and editing. **Bitrus Inuwa:** Formal analysis. **Ruth Iyasha Akintola:** Formal analysis. **Josiah Ibrahim:** Methodology. **Maryam Muhammad:** Conceptualization; funding acquisition; resources; supervision; validation; writing—review and editing.

## CONFLICT OF INTEREST STATEMENT

The authors declare no conflict of interest.

### PEER REVIEW

The peer review history for this article is available at https://www.webofscience.com/api/gateway/wos/peer-review/10.1111/irv.13254.

## ETHICS STATEMENT

Ethical approval for the study was obtained from the Animal Use and Care Committee of the National Veterinary Research Institute, Nigeria, under the number AEC/02/132/23. Access to the Hadejia‐Nguru wetland conservation was obtained from the National Park Service with approval reference number NPH/GEN/121/T/52.

## Supporting information


**Figure S1.** Maximum Likelihood phylogenetic tree of the PB2 gene segment obtained in IQtree v1.6.6. The viruses analyzed in this work are marked in pink and cluster within group A (yellow box) and group B (green box). The viruses previously detected in Nigeria are shown in blue. Ultrafast bootstrap values higher than 80 are shown next to the nodes.
**Figure S2.** Maximum Likelihood phylogenetic tree of the PB1 gene segment obtained in IQtree v1.6.6. The viruses analyzed in this work are marked in pink and cluster within group A (yellow box) and group B (green box). The viruses previously detected in Nigeria are shown in blue. Ultrafast bootstrap values higher than 80 are shown next to the nodes.
**Figure S3.** Maximum Likelihood phylogenetic tree of the PA gene segment obtained in IQtree v1.6.6. The viruses analyzed in this work are marked in pink and cluster within group A (yellow box) and group B (green box). The viruses previously detected in Nigeria are shown in blue. Ultrafast bootstrap values higher than 80 are shown next to the nodes.
**Figure S4.** Maximum Likelihood phylogenetic tree of the NP gene segment obtained in IQtree v1.6.6. The viruses analyzed in this work are marked in pink and cluster within group A (yellow box) and group B (green box). The viruses previously detected in Nigeria are shown in blue. Ultrafast bootstrap values higher than 80 are shown next to the nodes.
**Figure S5.** Maximum Likelihood phylogenetic tree of the NA gene segment obtained in IQtree v1.6.6. The viruses analyzed in this work are marked in pink and cluster within group A (yellow box) and group B (green box). The viruses previously detected in Nigeria are shown in blue. Ultrafast bootstrap values higher than 80 are shown next to the nodes.
**Figure S6.** Maximum Likelihood phylogenetic tree of the M gene segment obtained in IQtree v1.6.6. The viruses analyzed in this work are marked in pink and cluster within group A (yellow box) and group B (green box). The viruses previously detected in Nigeria are shown in blue. Ultrafast bootstrap values higher than 80 are shown next to the nodes.
**Figure S7.** Maximum Likelihood phylogenetic tree of the NS gene segment obtained in IQtree v1.6.6. The viruses analyzed in this work are marked in pink. The viruses previously detected in Nigeria are shown in blue. Ultrafast bootstrap values higher than 80 are shown next to the nodes.
**Table S1.** Sample information.
**Table S2.** Acknowledgment table of the authors, originating and submitting laboratories of the sequences from GISAID's EpiFlu™ Database on which this research is based in part. All submitters of data may be contacted directly via www.gisaid.org.

## Data Availability

The data that support the findings of this study are available in the supplementary material of this article.
