# Supplementary material for: Detection of clade 2.3.4.4 highly pathogenic avian influenza H5 viruses in healthy wild birds in the Hadeji‐Nguru wetland, Nigeria 2022
Source: Influenza Other Respir Viruses. 2024 Feb 3;18(2):e13254. doi: 10.1111/irv.13254 (PMC10837781; doi:10.1111/irv.13254)
Supplement: Supplementary file 1 — Figure S1. Maximum Likelihood phylogenetic tree of the PB2 gene segment obtained in IQtree v1.6.6. The viruses analyzed in this work are marked in pink and cluster within group A (yellow box) and group B (green box). The viruses previously detected in Nigeria are shown in blue. Ultrafast bootstrap values higher than 80 are shown next to the nodes. Figure S2. Maximum Likelihood phylogenetic tree of the PB1 gene segment obtained in IQtree v1.6.6. The viruses analyzed in this work are marked in pink and cluster within group A (yellow box) and group B (green box). The viruses previously detected in Nigeria are shown in blue. Ultrafast bootstrap values higher than 80 are shown next to the nodes. Figure S3. Maximum Likelihood phylogenetic tree of the PA gene segment obtained in IQtree v1.6.6. The viruses analyzed in this work are marked in pink and cluster within group A (yellow box) and group B (green box). The viruses previously detected in Nigeria are shown in blue. Ultrafast bootstrap values higher than 80 are shown next to the nodes. Figure S4. Maximum Likelihood phylogenetic tree of the NP gene segment obtained in IQtree v1.6.6. The viruses analyzed in this work are marked in pink and cluster within group A (yellow box) and group B (green box). The viruses previously detected in Nigeria are shown in blue. Ultrafast bootstrap values higher than 80 are shown next to the nodes. Figure S5. Maximum Likelihood phylogenetic tree of the NA gene segment obtained in IQtree v1.6.6. The viruses analyzed in this work are marked in pink and cluster within group A (yellow box) and group B (green box). The viruses previously detected in Nigeria are shown in blue. Ultrafast bootstrap values higher than 80 are shown next to the nodes. Figure S6. Maximum Likelihood phylogenetic tree of the M gene segment obtained in IQtree v1.6.6. The viruses analyzed in this work are marked in pink and cluster within group A (yellow box) and group B (green box). The viruses previously detected in Nig [file IRV-18-e13254-s001.docx]

**Supplementary material**

**Figure S1**. Maximum Likelihood phylogenetic tree of the PB2 gene segment obtained in IQtree v1.6.6. The viruses analyzed in this work are marked in pink and cluster within group A (yellow box) and group B (green box). The viruses previously detected in Nigeria are shown in blue. Ultrafast bootstrap values higher than 80 are shown next to the nodes.


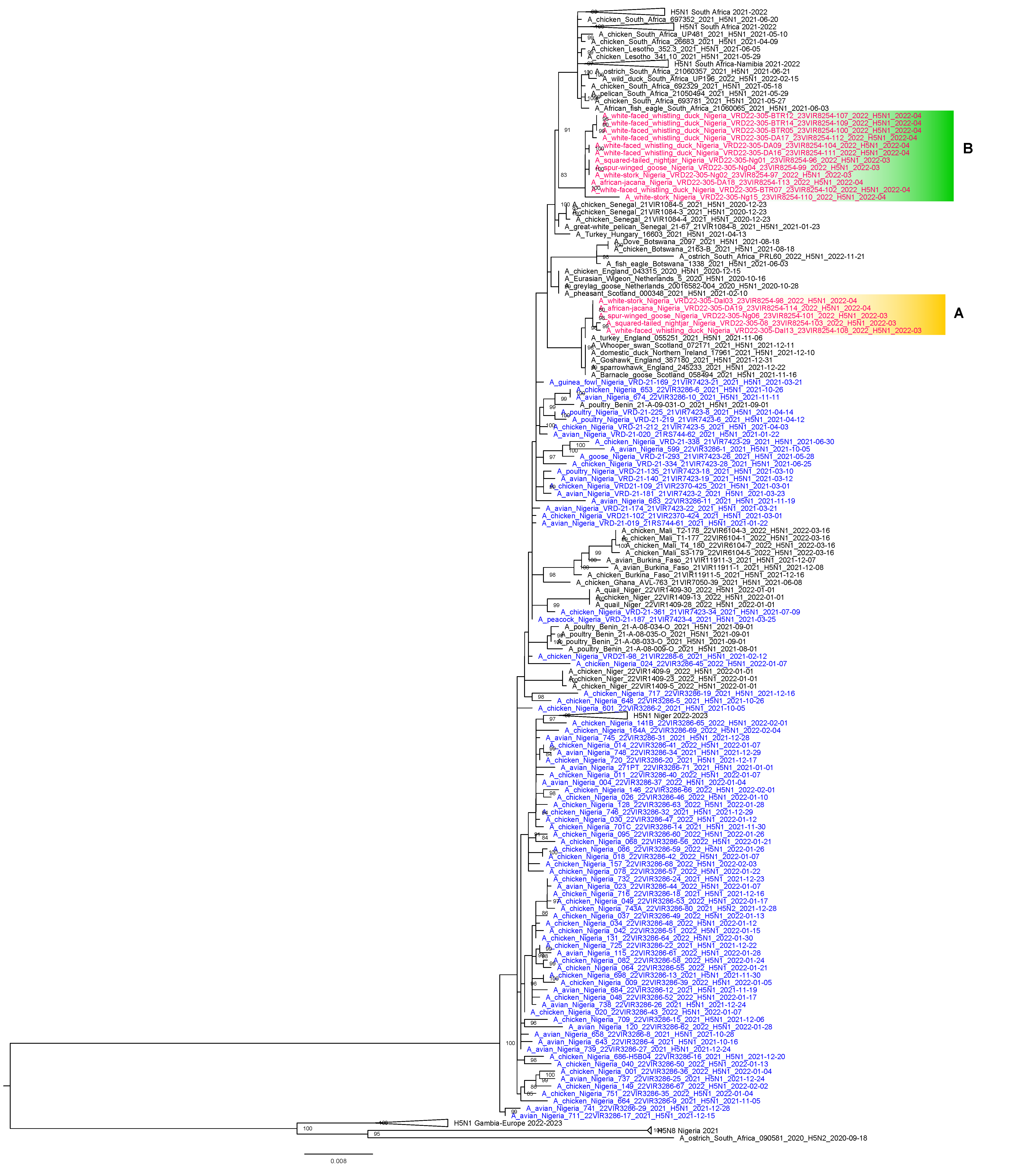


**Figure S2**. Maximum Likelihood phylogenetic tree of the PB1 gene segment obtained in IQtree v1.6.6. The viruses analyzed in this work are marked in pink and cluster within group A (yellow box) and group B (green box). The viruses previously detected in Nigeria are shown in blue. Ultrafast bootstrap values higher than 80 are shown next to the nodes.


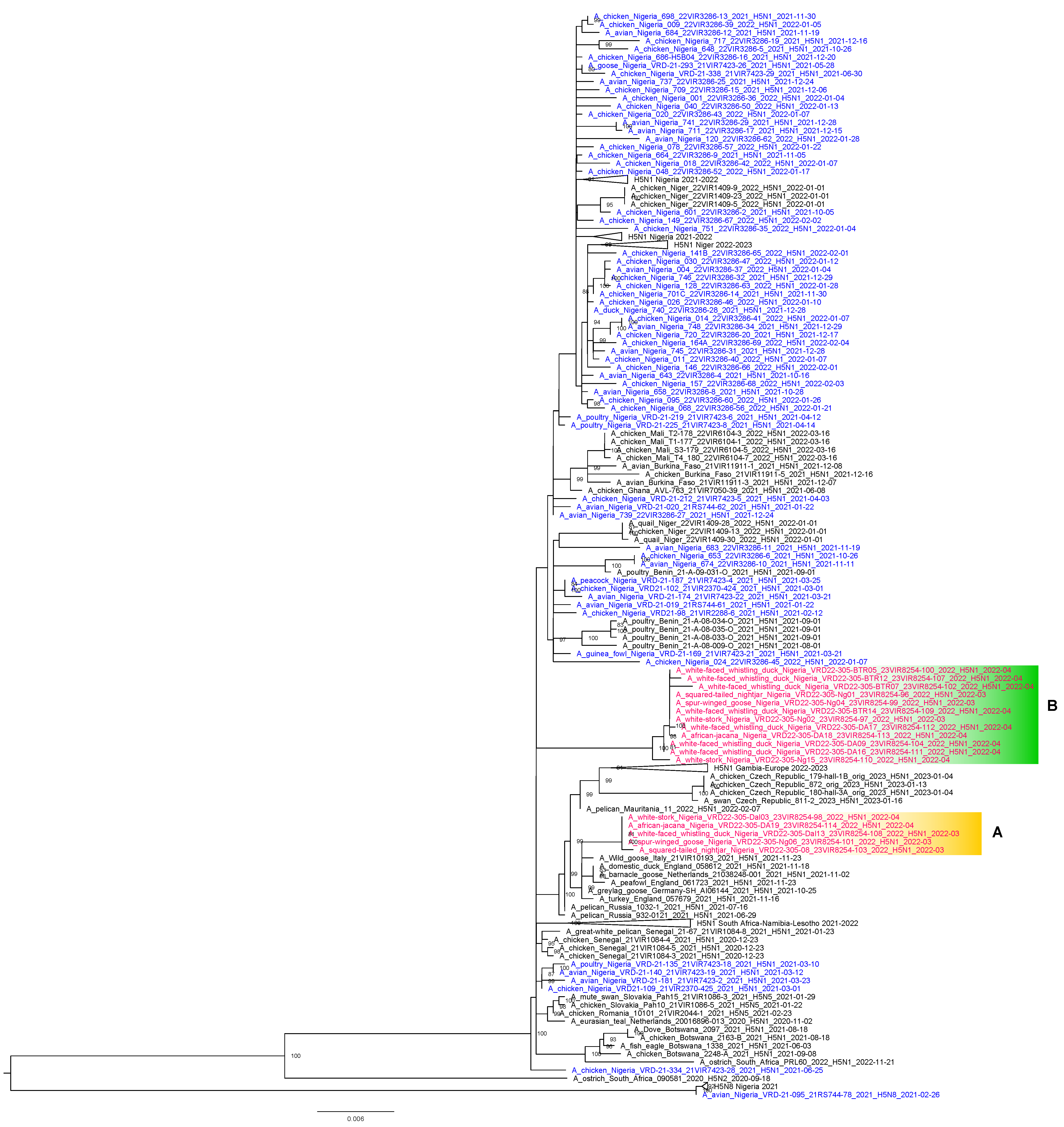


**Figure S3**. Maximum Likelihood phylogenetic tree of the PA gene segment obtained in IQtree v1.6.6. The viruses analyzed in this work are marked in pink and cluster within group A (yellow box) and group B (green box). The viruses previously detected in Nigeria are shown in blue. Ultrafast bootstrap values higher than 80 are shown next to the nodes.


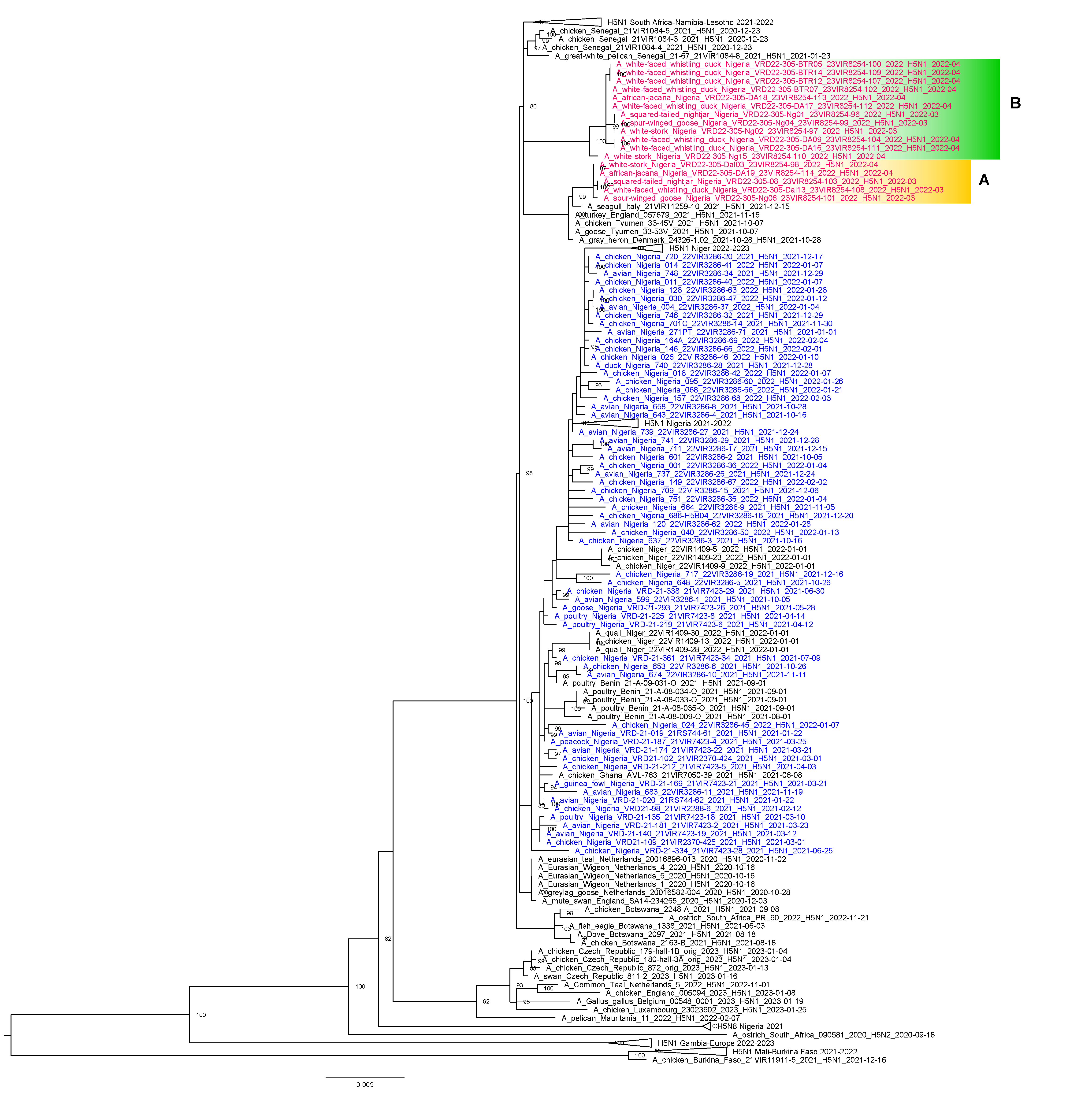


**Figure S4**. Maximum Likelihood phylogenetic tree of the NP gene segment obtained in IQtree v1.6.6. The viruses analyzed in this work are marked in pink and cluster within group A (yellow box) and group B (green box). The viruses previously detected in Nigeria are shown in blue. Ultrafast bootstrap values higher than 80 are shown next to the nodes.


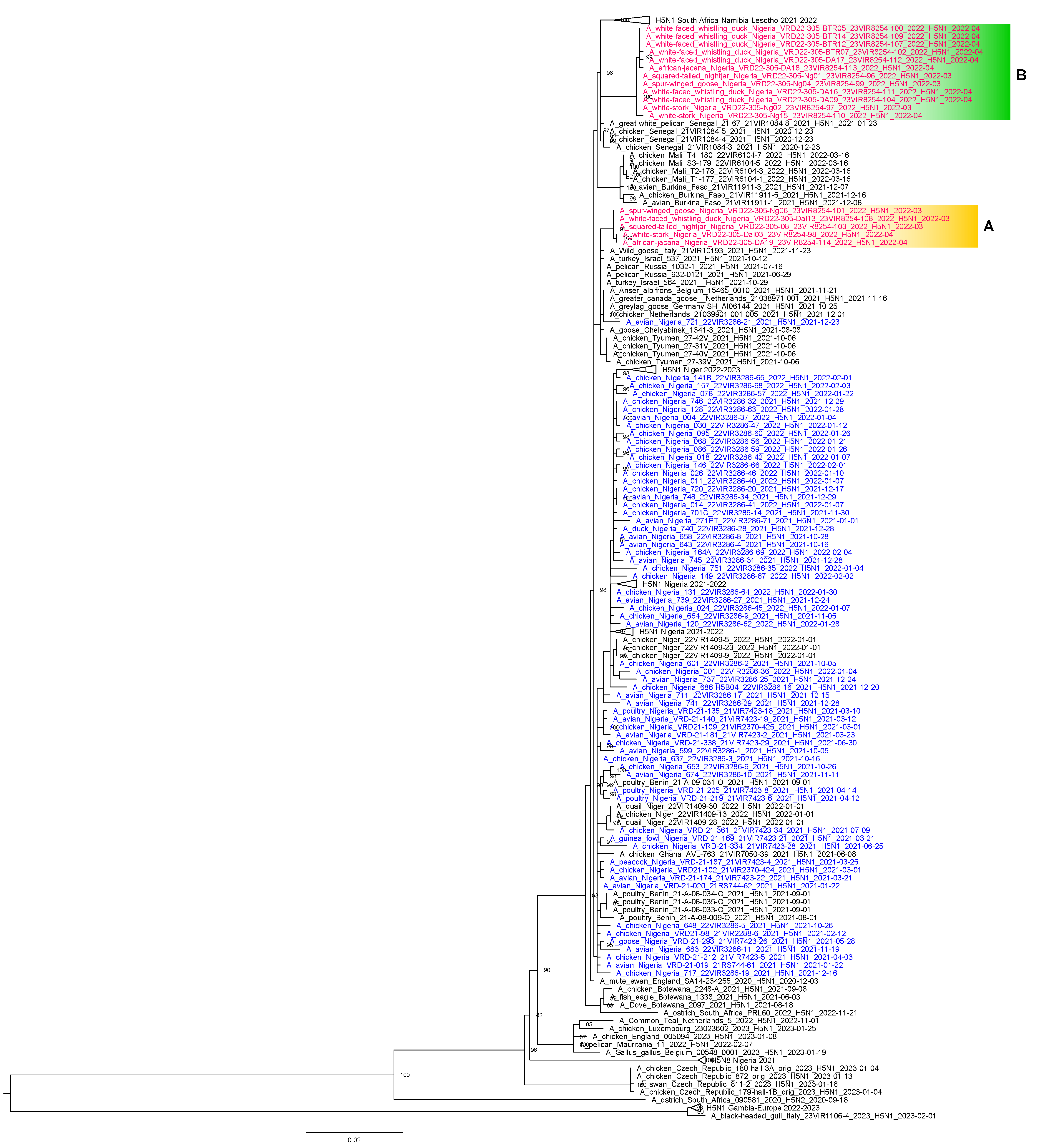


**Figure S5**. Maximum Likelihood phylogenetic tree of the NA gene segment obtained in IQtree v1.6.6. The viruses analyzed in this work are marked in pink and cluster within group A (yellow box) and group B (green box). The viruses previously detected in Nigeria are shown in blue. Ultrafast bootstrap values higher than 80 are shown next to the nodes.


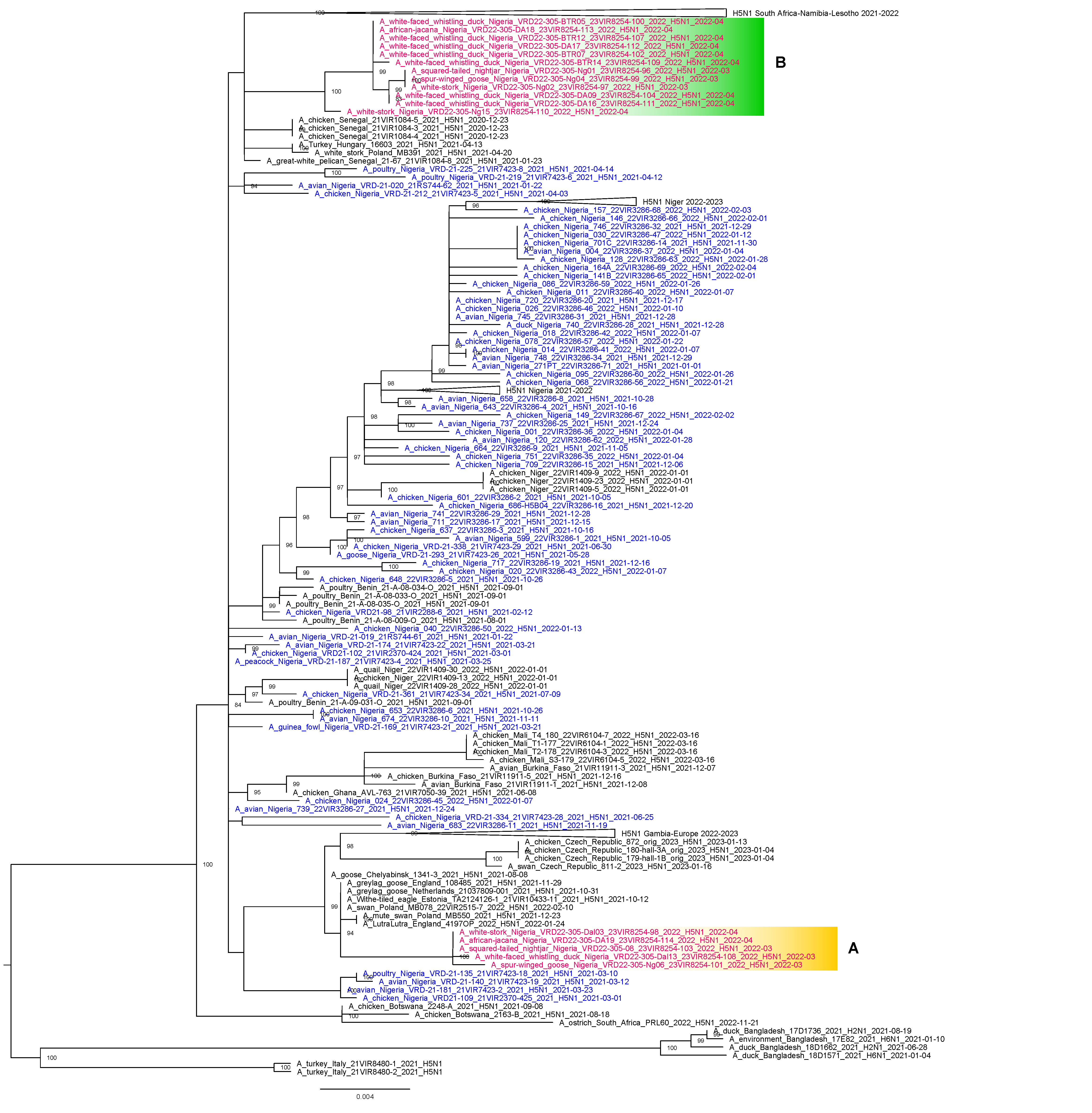


**Figure S6**. Maximum Likelihood phylogenetic tree of the M gene segment obtained in IQtree v1.6.6. The viruses analyzed in this work are marked in pink and cluster within group A (yellow box) and group B (green box). The viruses previously detected in Nigeria are shown in blue. Ultrafast bootstrap values higher than 80 are shown next to the nodes.


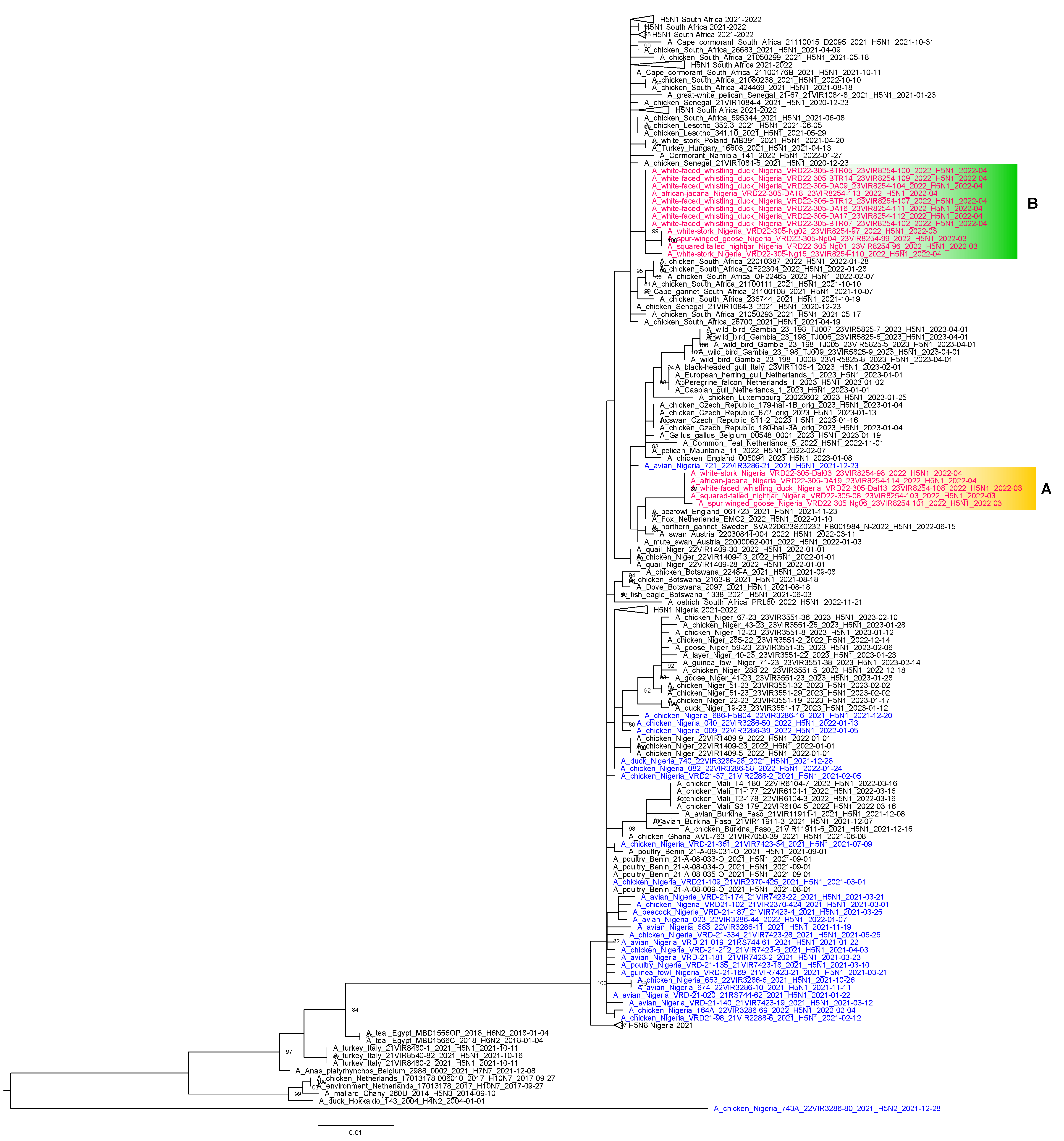

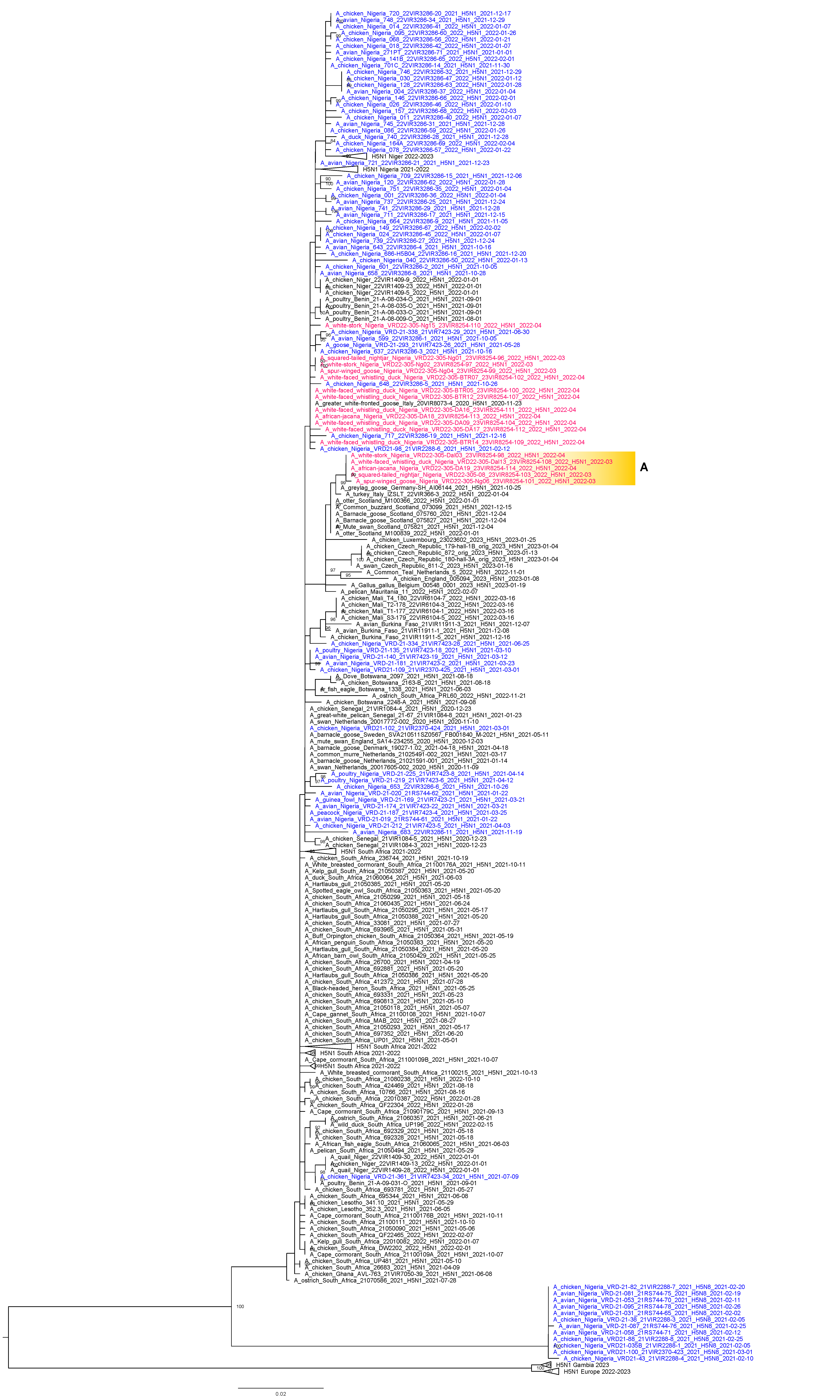


**Figure S7**. Maximum Likelihood phylogenetic tree of the NS gene segment obtained in IQtree v1.6.6. The viruses analyzed in this work are marked in pink. The viruses previously detected in Nigeria are shown in blue. Ultrafast bootstrap values higher than 80 are shown next to the nodes.

**Table S1.** Samples information.

| **Gisaid Isolate ID** | **Virus** | **Subtype** | **Country** | **Location** | **Host** | **Collection Date** |
| --- | --- | --- | --- | --- | --- | --- |
| EPI_ISL_18462524 | A/squared-tailed_nightjar/Nigeria/VRD22-305-Ng01_23VIR8254-96/2022 | H5N1 | Nigeria | Yobe | squared-tailed_nightjar | 2022-03 |
| EPI_ISL_18462525 | A/white-stork/Nigeria/VRD22-305-Ng02_23VIR8254-97/2022 | H5N1 | Nigeria | Yobe | white-stork | 2022-03 |
| EPI_ISL_18462526 | A/white-stork/Nigeria/VRD22-305-Dal03_23VIR8254-98/2022 | H5N1 | Nigeria | Yobe | white-stork | 2022-04 |
| EPI_ISL_18462527 | A/spur-winged_goose/Nigeria/VRD22-305-Ng04_23VIR8254-99/2022 | H5N1 | Nigeria | Yobe | spur-winged_goose | 2022-03 |
| EPI_ISL_18462528 | A/white-faced_whistling_duck/Nigeria/VRD22-305-BTR05_23VIR8254-100/2022 | H5N1 | Nigeria | Jigawa | white-faced_whistling_duck | 2022-04 |
| EPI_ISL_18462529 | A/spur-winged_goose/Nigeria/VRD22-305-Ng06_23VIR8254-101/2022 | H5N1 | Nigeria | Yobe | spur-winged_goose | 2022-03 |
| EPI_ISL_18462530 | A/white-faced_whistling_duck/Nigeria/VRD22-305-BTR07_23VIR8254-102/2022 | H5N1 | Nigeria | Jigawa | white-faced_whistling_duck | 2022-04 |
| EPI_ISL_18462531 | A/squared-tailed_nightjar/Nigeria/VRD22-305-08_23VIR8254-103/2022 | H5N1 | Nigeria | Yobe | squared-tailed_nightjar | 2022-03 |
| EPI_ISL_18462532 | A/white-faced_whistling_duck/Nigeria/VRD22-305-DA09_23VIR8254-104/2022 | H5N1 | Nigeria | Yobe | white-faced_whistling_duck | 2022-04 |
| EPI_ISL_18462533 | A/white-stork/Nigeria/VRD22-305-Ng10_23VIR8254-105/2022 | H5N1 | Nigeria | Yobe | white-stork | 2022-04 |
| EPI_ISL_18462534 | A/white-faced_whistling_duck/Nigeria/VRD22-305-BTR12_23VIR8254-107/2022 | H5N1 | Nigeria | Jigawa | white-faced_whistling_duck | 2022-04 |
| EPI_ISL_18462535 | A/white-faced_whistling_duck/Nigeria/VRD22-305-Dal13_23VIR8254-108/2022 | H5N1 | Nigeria | Yobe | white-faced_whistling_duck | 2022-03 |
| EPI_ISL_18462536 | A/white-faced_whistling_duck/Nigeria/VRD22-305-BTR14_23VIR8254-109/2022 | H5N1 | Nigeria | Jigawa-Baturia | white-faced_whistling_duck | 2022-04 |
| EPI_ISL_18462537 | A/white-stork/Nigeria/VRD22-305-Ng15_23VIR8254-110/2022 | H5N1 | Nigeria | Yobe-Nguru | white-stork | 2022-04 |
| EPI_ISL_18462538 | A/white-faced_whistling_duck/Nigeria/VRD22-305-DA16_23VIR8254-111/2022 | H5N1 | Nigeria | Yobe-Dagona | white-faced_whistling_duck | 2022-04 |
| EPI_ISL_18462539 | A/white-faced_whistling_duck/Nigeria/VRD22-305-DA17_23VIR8254-112/2022 | H5N1 | Nigeria | Yobe-Dagona | white-faced_whistling_duck | 2022-04 |
| EPI_ISL_18462540 | A/african-jacana/Nigeria/VRD22-305-DA18_23VIR8254-113/2022 | H5N1 | Nigeria | Yobe-Dagona | african-jacana | 2022-04 |
| EPI_ISL_18462541 | A/african-jacana/Nigeria/VRD22-305-DA19_23VIR8254-114/2022 | H5N1 | Nigeria | Yobe-Dagona | african-jacana | 2022-04 |

**Table S2.** Acknowledgement table of the authors, originating and submitting laboratories of the sequences from GISAID’s EpiFlu™ Database on which this research is based in part. All submitters of data may be contacted directly via www.gisaid.org.

| **Isolate-ID** | **Country** | **Collection date** | **Isolate name** | **Originating Lab** | **Submitting Lab** | **Authors** |
| --- | --- | --- | --- | --- | --- | --- |
| [EPI_ISL_17414662](https://platform.epicov.org/epi3/start/EPI_ISL/17414662) | Nigeria | 2021-May-28 | A/goose/Nigeria/VRD-21-293_21VIR7423-26/2021 | National Veterinary Research Institute | Istituto Zooprofilattico Sperimentale Delle Venezie | Meseko, C.; Milani, A.; Inuwa, B.; Chinyere, C.; Shittu, I.; Ahmed, J.; Giussani, E.; Palumbo, E.; Zecchin, B.; Bonfante, F.; Maniero, S.; Fusaro, A.; Gobbo, F.; Terregino, C.; Olasoju, T.; Monne, I.; Muhammad, M. |
| [EPI_ISL_17638503](https://platform.epicov.org/epi3/start/EPI_ISL/17638503) | Niger | 2023-Feb-06 | A/goose/Niger/59-23_23VIR3551-35/2023 | Laboratoire Central de l'Elevage (LABOCEL) | Istituto Zooprofilattico Sperimentale Delle Venezie | Souley, M. M.; Milani, A.; Yaou, B.; Amadou, H.; Haido, A. M.; Issiako, A.; Varotto, M.; Giussani, E.; Palumbo, E.; Zecchin, B.; Fusaro, A. |
| [EPI_ISL_17638499](https://platform.epicov.org/epi3/start/EPI_ISL/17638499) | Niger | 2023-Jan-28 | A/goose/Niger/41-23_23VIR3551-23/2023 | Laboratoire Central de l'Elevage (LABOCEL) | Istituto Zooprofilattico Sperimentale Delle Venezie | Souley, M. M.; Milani, A.; Yaou, B.; Amadou, H.; Haido, A. M.; Issiako, A.; Varotto, M.; Giussani, E.; Palumbo, E.; Zecchin, B.; Fusaro, A. |
| [EPI_ISL_14643356](https://platform.epicov.org/epi3/start/EPI_ISL/14643356) | South Africa | 2021-May-20 | A/Hartlaubs gull/South Africa/21050386/2021 (H5N1) | Western Cape Provincial Veterinary Laboratory | University of Pretoria | Abolnik, Celia |
| [EPI_ISL_14640323](https://platform.epicov.org/epi3/start/EPI_ISL/14640323) | South Africa | 2021-May-20 | A/Hartlaubs gull/South Africa/21050384/2021 (H5N1) | Western Cape Provincial Veterinary Laboratory | University of Pretoria | Abolnik, Celia |
| [EPI_ISL_14639383](https://platform.epicov.org/epi3/start/EPI_ISL/14639383) | South Africa | 2021-May-20 | A/Kelp gull/South Africa/21050387/2021 (H5N1) | Western Cape Provincial Veterinary Laboratory | University of Pretoria | Abolnik, Celia |
| [EPI_ISL_14638201](https://platform.epicov.org/epi3/start/EPI_ISL/14638201) | South Africa | 2021-May-20 | A/Hartlaubs gull/South Africa/21050388/2021 (H5N1) | Western Cape Provincial Veterinary Laboratory | University of Pretoria | Abolnik, Celia |
| [EPI_ISL_14637676](https://platform.epicov.org/epi3/start/EPI_ISL/14637676) | South Africa | 2021-May-20 | A/Hartlaubs gull/21050385/2021 (H5N1) | Western Cape Provincial Veterinary Laboratory | University of Pretoria | Abolnik, Celia |
| [EPI_ISL_14619009](https://platform.epicov.org/epi3/start/EPI_ISL/14619009) | South Africa | 2021-May-17 | A/Hartlaubs gull/South Africa/21050295/2021 (H5N1) | Western Cape Provincial Veterinary Laboratory | University of Pretoria | Abolnik, Celia |
| [EPI_ISL_15852552](https://platform.epicov.org/epi3/start/EPI_ISL/15852552) | South Africa | 2022-Jan-07 | A/Kelp gull/South Africa/22010082/2022 | Western Cape Provincial Veterinary Laboratory | University of Pretoria | Abolnik, C. |
| [EPI_ISL_14933722](https://platform.epicov.org/epi3/start/EPI_ISL/14933722) | South Africa | 2021-Jul-28 | A/ostrich/South Africa/21070586/2021 (H5N1) | Western Cape Provincial Veterinary Laboratory | University of Pretoria | Abolnik, Celia |
| [EPI_ISL_14918327](https://platform.epicov.org/epi3/start/EPI_ISL/14918327) | South Africa | 2021-Jun-23 | A/ostrich/South Africa/21060425/2021 (H5N1) | Western Cape Provincial Veterinary Laboratory | University of Pretoria | Abolnik, Celia |
| [EPI_ISL_14918305](https://platform.epicov.org/epi3/start/EPI_ISL/14918305) | South Africa | 2021-Jun-21 | A/ostrich/South Africa/21060357/2021 (H5N1) | Western Cape Provincial Veterinary Laboratory | University of Pretoria | Abolnik, Celia |
| [EPI_ISL_14918030](https://platform.epicov.org/epi3/start/EPI_ISL/14918030) | South Africa | 2021-Jun-14 | A/ostrich/South Africa/21060311/2021 (H5N1) | Western Cape Provincial Veterinary Laboratory | University of Pretoria | Abolnik, Celia |
| [EPI_ISL_16675872](https://platform.epicov.org/epi3/start/EPI_ISL/16675872) | South Africa | 2022-Nov-21 | A/ostrich/South Africa/PRL60/2022 | Faculty of Veterinary Science, University of Pretoria | University of Pretoria | Abolnik, C. |
| [EPI_ISL_15852186](https://platform.epicov.org/epi3/start/EPI_ISL/15852186) | South Africa | 2021-Nov-11 | A/seabird/South Africa/21110253/2021 | Western Cape Provincial Veterinary Laboratory | University of Pretoria | Abolnik, C. |
| [EPI_ISL_14973559](https://platform.epicov.org/epi3/start/EPI_ISL/14973559) | South Africa | 2021-Oct-07 | A/Cape gannet/South Africa/21100108/2021 (H5N1) | Western Cape Provincial Veterinary Laboratory | University of Pretoria | Abolnik, Celia |
| [EPI_ISL_14645331](https://platform.epicov.org/epi3/start/EPI_ISL/14645331) | South Africa | 2021-May-25 | A/Black-headed heron/South Africa/2021 (H5N1) | Western Cape Provincial Veterinary Laboratory | University of Pretoria | Abolnik, Celia |
| [EPI_ISL_14639837](https://platform.epicov.org/epi3/start/EPI_ISL/14639837) | South Africa | 2021-May-20 | A/Spotted eagle owl/South Africa/21050363/2021 (H5N1) | Western Cape Provincial Veterinary Laboratory | University of Pretoria | Abolnik, Celia |
| [EPI_ISL_15853010](https://platform.epicov.org/epi3/start/EPI_ISL/15853010) | South Africa | 2022-Feb-08 | A/European white stork/South Africa/BA107/2022 | Faculty of Veterinary Science, University of Pretoria | University of Pretoria | Abolnik, C. |
| [EPI_ISL_17071946](https://platform.epicov.org/epi3/start/EPI_ISL/17071946) | South Africa | 2022-Nov-04 | A/Cape gannet/South Africa/702625 G123/2022 | Assurecloud (Pty) Ltd | University of Pretoria | Abolnik, C. |
| [EPI_ISL_15853934](https://platform.epicov.org/epi3/start/EPI_ISL/15853934) | South Africa | 2022-Aug-01 | A/Common tern/South Africa/693799 DOA236/2022 | Assurecloud (Pty) Ltd | University of Pretoria | Abolnik, C. |
| [EPI_ISL_15853920](https://platform.epicov.org/epi3/start/EPI_ISL/15853920) | South Africa | 2022-May-10 | A/Common tern/South Africa/686940 CT013/2022 | Assurecloud (Pty) Ltd | University of Pretoria | Abolnik, C. |
| [EPI_ISL_15853835](https://platform.epicov.org/epi3/start/EPI_ISL/15853835) | South Africa | 2022-Mar-28 | A/African black oystercatcher/South Africa/682489/2022 | Assurecloud (Pty) Ltd | University of Pretoria | Abolnik, C. |
| [EPI_ISL_15853610](https://platform.epicov.org/epi3/start/EPI_ISL/15853610) | South Africa | 2022-Mar-22 | A/Swift tern/South Africa/681998 ST009/2022 | Assurecloud (Pty) Ltd | University of Pretoria | Abolnik, C. |
| [EPI_ISL_15853608](https://platform.epicov.org/epi3/start/EPI_ISL/15853608) | South Africa | 2022-Mar-21 | A/Common tern/South Africa/681998 CT010/2022 | Assurecloud (Pty) Ltd | University of Pretoria | Abolnik, C. |
| [EPI_ISL_11007545](https://platform.epicov.org/epi3/start/EPI_ISL/11007545) | Niger | 2022-Jan-01 | A/quail/Niger/22VIR1409-30/2022 | Laboratoire Central de l’Elevage (LABOCEL) | Istituto Zooprofilattico Sperimentale delle Venezie | Souley, M.M.; Yaou, B.; Amadou, H.; Haido, A.M.; Issiako, A.; Alassane, A.; Barbierato, G.; Zecchin, B.; Fusaro, A.; Schivo, A.; Salviato, A.; Palumbo, E.; Giussani, E.; Monne, I.; Terregino, C. |
| [EPI_ISL_11007544](https://platform.epicov.org/epi3/start/EPI_ISL/11007544) | Niger | 2022-Jan-01 | A/quail/Niger/22VIR1409-28/2022 | Laboratoire Central de l’Elevage (LABOCEL) | Istituto Zooprofilattico Sperimentale delle Venezie | Souley, M.M.; Yaou, B.; Amadou, H.; Haido, A.M.; Issiako, A.; Alassane, A.; Barbierato, G.; Zecchin, B.; Fusaro, A.; Schivo, A.; Salviato, A.; Palumbo, E.; Giussani, E.; Monne, I.; Terregino, C. |
| [EPI_ISL_17634330](https://platform.epicov.org/epi3/start/EPI_ISL/17634330) | Mauritania | 2022-Feb-07 | A/pelican/Mauritania/11/2022 | na | na | Beyit,A.D.; Meki,I.K.; Barry,Y.; Haki,M.L.; El Ghassem,A.; Hamma,S.M.; Abdelwahab,N.; Doumbia,B.; Ahmed Benane,H.; Daf,D.S.; Sidatt,Z.E.A.; Ould Mekhalla,L.; El Mamy,B.; Gueya,M.O.B.; Settypalli,T.B.K.; Ouled Ahmed Ben Ali,H.; Datta,S.; Cattoli,G.; Lamien,C.E.; Dundon,W.G.; Yahya,B.; Elghassem,A.; Baba,D.; Benane,H.A.; El Abidine Sidatt,Z.; Mekhalla,L.O.; Baba,M.O. |
| [EPI_ISL_16839065](https://platform.epicov.org/epi3/start/EPI_ISL/16839065) | Namibia | 2022-Jan-27 | A/Cormorant/Namibia/141/2022 | na | na | Molini,U.; Yabe,J.; Meki,I.K.; Ahmed Ben Ali,H.O.; Settypalli,T.B.K.; Datta,S.; Coetzee,L.M.; Hamunyela,E.; Khaiseb,S.; Cattoli,G.; Lamien,C.E.; Dundon,W.G.; Ouled Ahmed Ben Ali,H. |
| [EPI_ISL_18127714](https://platform.epicov.org/epi3/start/EPI_ISL/18127714) | Gambia | 2023-Apr-01 | A/wild_bird/Gambia/23_198_TJ009_23VIR5825-9/2023 | Istituto Zooprofilattico Sperimentale delle Venezie, EU/OIE/Reference Laboratory and FAO Reference Centre for AI and ND | Istituto Zooprofilattico Sperimentale Delle Venezie | Ceesay, O.; Ceesay, A.; Bah, S.A.; Sambou, B.; Kassama, M.L.; Pastori, A.; Zecchin, B.; Fusaro, A.; Schivo, A.; Salviato, A.; Palumbo, E.; Giussani, E.; Monne, I.; Terregino, C. |
| [EPI_ISL_18127713](https://platform.epicov.org/epi3/start/EPI_ISL/18127713) | Gambia | 2023-Apr-01 | A/wild_bird/Gambia/23_198_TJ008_23VIR5825-8/2023 | Istituto Zooprofilattico Sperimentale delle Venezie, EU/OIE/Reference Laboratory and FAO Reference Centre for AI and ND | Istituto Zooprofilattico Sperimentale Delle Venezie | Ceesay, O.; Ceesay, A.; Bah, S.A.; Sambou, B.; Kassama, M.L.; Pastori, A.; Zecchin, B.; Fusaro, A.; Schivo, A.; Salviato, A.; Palumbo, E.; Giussani, E.; Monne, I.; Terregino, C. |
| [EPI_ISL_18127712](https://platform.epicov.org/epi3/start/EPI_ISL/18127712) | Gambia | 2023-Apr-01 | A/wild_bird/Gambia/23_198_TJ007_23VIR5825-7/2023 | Istituto Zooprofilattico Sperimentale delle Venezie, EU/OIE/Reference Laboratory and FAO Reference Centre for AI and ND | Istituto Zooprofilattico Sperimentale Delle Venezie | Ceesay, O.; Ceesay, A.; Bah, S.A.; Sambou, B.; Kassama, M.L.; Pastori, A.; Zecchin, B.; Fusaro, A.; Schivo, A.; Salviato, A.; Palumbo, E.; Giussani, E.; Monne, I.; Terregino, C. |
| [EPI_ISL_18127711](https://platform.epicov.org/epi3/start/EPI_ISL/18127711) | Gambia | 2023-Apr-01 | A/wild_bird/Gambia/23_198_TJ006_23VIR5825-6/2023 | Istituto Zooprofilattico Sperimentale delle Venezie, EU/OIE/Reference Laboratory and FAO Reference Centre for AI and ND | Istituto Zooprofilattico Sperimentale Delle Venezie | Ceesay, O.; Ceesay, A.; Bah, S.A.; Sambou, B.; Kassama, M.L.; Pastori, A.; Zecchin, B.; Fusaro, A.; Schivo, A.; Salviato, A.; Palumbo, E.; Giussani, E.; Monne, I.; Terregino, C. |
| [EPI_ISL_18127710](https://platform.epicov.org/epi3/start/EPI_ISL/18127710) | Gambia | 2023-Apr-01 | A/wild_bird/Gambia/23_198_TJ005_23VIR5825-5/2023 | Istituto Zooprofilattico Sperimentale delle Venezie, EU/OIE/Reference Laboratory and FAO Reference Centre for AI and ND | Istituto Zooprofilattico Sperimentale Delle Venezie | Ceesay, O.; Ceesay, A.; Bah, S.A.; Sambou, B.; Kassama, M.L.; Pastori, A.; Zecchin, B.; Fusaro, A.; Schivo, A.; Salviato, A.; Palumbo, E.; Giussani, E.; Monne, I.; Terregino, C. |
| [EPI_ISL_15853343](https://platform.epicov.org/epi3/start/EPI_ISL/15853343) | South Africa | 2022-Feb-15 | A/wild duck/South Africa/UP196/2022 | Faculty of Veterinary Science, University of Pretoria | University of Pretoria | Abolnik, C. |
| [EPI_ISL_14934885](https://platform.epicov.org/epi3/start/EPI_ISL/14934885) | South Africa | 2021-Sep-13 | A/Cape cormorant/South Africa/21090179C/2021 (H5N1) | Western Cape Provincial Veterinary Laboratory | University of Pretoria | Abolnik, Celia |
| [EPI_ISL_14934885](https://platform.epicov.org/epi3/start/EPI_ISL/14934885) | South Africa | 2021-Sep-13 | A/Cape cormorant/South Africa/21090179C/2021 (H5N1) | Western Cape Provincial Veterinary Laboratory | University of Pretoria | Abolnik, Celia |
| [EPI_ISL_14918893](https://platform.epicov.org/epi3/start/EPI_ISL/14918893) | South Africa | 2021-Jul-01 | A/Blue crane/South Africa/21070050/2021 (H5N1) | Western Cape Provincial Veterinary Laboratory | University of Pretoria | Abolnik, Celia |
| [EPI_ISL_14918839](https://platform.epicov.org/epi3/start/EPI_ISL/14918839) | South Africa | 2021-Jun-28 | A/Blue crane/South Africa/21060475/2021 (H5N1) | Western Cape Provincial Veterinary Laboratory | University of Pretoria | Abolnik, Celia |
| [EPI_ISL_14918031](https://platform.epicov.org/epi3/start/EPI_ISL/14918031) | South Africa | 2021-Jun-19 | A/Sacred ibis/South Africa/21060395/2021 (H5N1) | Western Cape Provincial Veterinary Laboratory | University of Pretoria | Abolnik, Celia |
| [EPI_ISL_14646080](https://platform.epicov.org/epi3/start/EPI_ISL/14646080) | South Africa | 2021-May-29 | A/pelican/South Africa/21050494/2021 (H5N1) | Western Cape Provincial Veterinary Laboratory | University of Pretoria | Abolnik, C |
| [EPI_ISL_14644592](https://platform.epicov.org/epi3/start/EPI_ISL/14644592) | South Africa | 2021-May-25 | A/African barn owl/South Africa/21050429/2021 (H5N1) | Western Cape Provincial Veterinary Laboratory | University of Pretoria | Abolnik, Celia |
| [EPI_ISL_17415170](https://platform.epicov.org/epi3/start/EPI_ISL/17415170) | Nigeria | 2021-Jan-01 | A/avian/Nigeria/271PT_22VIR3286-71/2021 | National Veterinary Research Institute | Istituto Zooprofilattico Sperimentale Delle Venezie | Meseko, C.; Milani, A.; Inuwa, B.; Chinyere, C.; Shittu, I.; Ahmed, J.; Giussani, E.; Palumbo, E.; Zecchin, B.; Bonfante, F.; Maniero, S.; Fusaro, A.; Gobbo, F.; Terregino, C.; Olasoju, T.; Monne, I.; Muhammad, M. |
| [EPI_ISL_17414668](https://platform.epicov.org/epi3/start/EPI_ISL/17414668) | Nigeria | 2021-Mar-21 | A/guinea_fowl/Nigeria/VRD-21-169_21VIR7423-21/2021 | National Veterinary Research Institute | Istituto Zooprofilattico Sperimentale Delle Venezie | Meseko, C.; Milani, A.; Inuwa, B.; Chinyere, C.; Shittu, I.; Ahmed, J.; Giussani, E.; Palumbo, E.; Zecchin, B.; Bonfante, F.; Maniero, S.; Fusaro, A.; Gobbo, F.; Terregino, C.; Olasoju, T.; Monne, I.; Muhammad, M. |
| [EPI_ISL_17414663](https://platform.epicov.org/epi3/start/EPI_ISL/17414663) | Nigeria | 2021-Mar-25 | A/peacock/Nigeria/VRD-21-187_21VIR7423-4/2021 | National Veterinary Research Institute | Istituto Zooprofilattico Sperimentale Delle Venezie | Meseko, C.; Milani, A.; Inuwa, B.; Chinyere, C.; Shittu, I.; Ahmed, J.; Giussani, E.; Palumbo, E.; Zecchin, B.; Bonfante, F.; Maniero, S.; Fusaro, A.; Gobbo, F.; Terregino, C.; Olasoju, T.; Monne, I.; Muhammad, M. |
| [EPI_ISL_17414610](https://platform.epicov.org/epi3/start/EPI_ISL/17414610) | Nigeria | 2021-Mar-23 | A/avian/Nigeria/VRD-21-181_21VIR7423-2/2021 | National Veterinary Research Institute | Istituto Zooprofilattico Sperimentale Delle Venezie | Meseko, C.; Milani, A.; Inuwa, B.; Chinyere, C.; Shittu, I.; Ahmed, J.; Giussani, E.; Palumbo, E.; Zecchin, B.; Bonfante, F.; Maniero, S.; Fusaro, A.; Gobbo, F.; Terregino, C.; Olasoju, T.; Monne, I.; Muhammad, M. |
| [EPI_ISL_17414609](https://platform.epicov.org/epi3/start/EPI_ISL/17414609) | Nigeria | 2021-Mar-21 | A/avian/Nigeria/VRD-21-174_21VIR7423-22/2021 | National Veterinary Research Institute | Istituto Zooprofilattico Sperimentale Delle Venezie | Meseko, C.; Milani, A.; Inuwa, B.; Chinyere, C.; Shittu, I.; Ahmed, J.; Giussani, E.; Palumbo, E.; Zecchin, B.; Bonfante, F.; Maniero, S.; Fusaro, A.; Gobbo, F.; Terregino, C.; Olasoju, T.; Monne, I.; Muhammad, M. |
| [EPI_ISL_17414608](https://platform.epicov.org/epi3/start/EPI_ISL/17414608) | Nigeria | 2021-Mar-12 | A/avian/Nigeria/VRD-21-140_21VIR7423-19/2021 | National Veterinary Research Institute | Istituto Zooprofilattico Sperimentale Delle Venezie | Meseko, C.; Milani, A.; Inuwa, B.; Chinyere, C.; Shittu, I.; Ahmed, J.; Giussani, E.; Palumbo, E.; Zecchin, B.; Bonfante, F.; Maniero, S.; Fusaro, A.; Gobbo, F.; Terregino, C.; Olasoju, T.; Monne, I.; Muhammad, M. |
| [EPI_ISL_17414607](https://platform.epicov.org/epi3/start/EPI_ISL/17414607) | Nigeria | 2021-Feb-26 | A/avian/Nigeria/VRD-21-095_21RS744-78/2021 | National Veterinary Research Institute | Istituto Zooprofilattico Sperimentale Delle Venezie | Meseko, C.; Milani, A.; Inuwa, B.; Chinyere, C.; Shittu, I.; Ahmed, J.; Giussani, E.; Palumbo, E.; Zecchin, B.; Bonfante, F.; Maniero, S.; Fusaro, A.; Gobbo, F.; Terregino, C.; Olasoju, T.; Monne, I.; Muhammad, M. |
| [EPI_ISL_17414606](https://platform.epicov.org/epi3/start/EPI_ISL/17414606) | Nigeria | 2021-Feb-25 | A/avian/Nigeria/VRD-21-087_21RS744-76/2021 | National Veterinary Research Institute | Istituto Zooprofilattico Sperimentale Delle Venezie | Meseko, C.; Milani, A.; Inuwa, B.; Chinyere, C.; Shittu, I.; Ahmed, J.; Giussani, E.; Palumbo, E.; Zecchin, B.; Bonfante, F.; Maniero, S.; Fusaro, A.; Gobbo, F.; Terregino, C.; Olasoju, T.; Monne, I.; Muhammad, M. |
| [EPI_ISL_17414605](https://platform.epicov.org/epi3/start/EPI_ISL/17414605) | Nigeria | 2021-Feb-19 | A/avian/Nigeria/VRD-21-081_21RS744-75/2021 | National Veterinary Research Institute | Istituto Zooprofilattico Sperimentale Delle Venezie | Meseko, C.; Milani, A.; Inuwa, B.; Chinyere, C.; Shittu, I.; Ahmed, J.; Giussani, E.; Palumbo, E.; Zecchin, B.; Bonfante, F.; Maniero, S.; Fusaro, A.; Gobbo, F.; Terregino, C.; Olasoju, T.; Monne, I.; Muhammad, M. |
| [EPI_ISL_17414604](https://platform.epicov.org/epi3/start/EPI_ISL/17414604) | Nigeria | 2021-Feb-12 | A/avian/Nigeria/VRD-21-058_21RS744-71/2021 | National Veterinary Research Institute | Istituto Zooprofilattico Sperimentale Delle Venezie | Meseko, C.; Milani, A.; Inuwa, B.; Chinyere, C.; Shittu, I.; Ahmed, J.; Giussani, E.; Palumbo, E.; Zecchin, B.; Bonfante, F.; Maniero, S.; Fusaro, A.; Gobbo, F.; Terregino, C.; Olasoju, T.; Monne, I.; Muhammad, M. |
| [EPI_ISL_17414603](https://platform.epicov.org/epi3/start/EPI_ISL/17414603) | Nigeria | 2021-Feb-11 | A/avian/Nigeria/VRD-21-053_21RS744-70/2021 | National Veterinary Research Institute | Istituto Zooprofilattico Sperimentale Delle Venezie | Meseko, C.; Milani, A.; Inuwa, B.; Chinyere, C.; Shittu, I.; Ahmed, J.; Giussani, E.; Palumbo, E.; Zecchin, B.; Bonfante, F.; Maniero, S.; Fusaro, A.; Gobbo, F.; Terregino, C.; Olasoju, T.; Monne, I.; Muhammad, M. |
| [EPI_ISL_17414602](https://platform.epicov.org/epi3/start/EPI_ISL/17414602) | Nigeria | 2021-Feb-02 | A/avian/Nigeria/VRD-21-031_21RS744-65/2021 | National Veterinary Research Institute | Istituto Zooprofilattico Sperimentale Delle Venezie | Meseko, C.; Milani, A.; Inuwa, B.; Chinyere, C.; Shittu, I.; Ahmed, J.; Giussani, E.; Palumbo, E.; Zecchin, B.; Bonfante, F.; Maniero, S.; Fusaro, A.; Gobbo, F.; Terregino, C.; Olasoju, T.; Monne, I.; Muhammad, M. |
| [EPI_ISL_17414601](https://platform.epicov.org/epi3/start/EPI_ISL/17414601) | Nigeria | 2021-Jan-22 | A/avian/Nigeria/VRD-21-020_21RS744-62/2021 | National Veterinary Research Institute | Istituto Zooprofilattico Sperimentale Delle Venezie | Meseko, C.; Milani, A.; Inuwa, B.; Chinyere, C.; Shittu, I.; Ahmed, J.; Giussani, E.; Palumbo, E.; Zecchin, B.; Bonfante, F.; Maniero, S.; Fusaro, A.; Gobbo, F.; Terregino, C.; Olasoju, T.; Monne, I.; Muhammad, M. |
| [EPI_ISL_17414600](https://platform.epicov.org/epi3/start/EPI_ISL/17414600) | Nigeria | 2021-Jan-22 | A/avian/Nigeria/VRD-21-019_21RS744-61/2021 | National Veterinary Research Institute | Istituto Zooprofilattico Sperimentale Delle Venezie | Meseko, C.; Milani, A.; Inuwa, B.; Chinyere, C.; Shittu, I.; Ahmed, J.; Giussani, E.; Palumbo, E.; Zecchin, B.; Bonfante, F.; Maniero, S.; Fusaro, A.; Gobbo, F.; Terregino, C.; Olasoju, T.; Monne, I.; Muhammad, M. |
| [EPI_ISL_17414599](https://platform.epicov.org/epi3/start/EPI_ISL/17414599) | Nigeria | 2021-Dec-29 | A/avian/Nigeria/748_22VIR3286-34/2021 | National Veterinary Research Institute | Istituto Zooprofilattico Sperimentale Delle Venezie | Meseko, C.; Milani, A.; Inuwa, B.; Chinyere, C.; Shittu, I.; Ahmed, J.; Giussani, E.; Palumbo, E.; Zecchin, B.; Bonfante, F.; Maniero, S.; Fusaro, A.; Gobbo, F.; Terregino, C.; Olasoju, T.; Monne, I.; Muhammad, M. |
| [EPI_ISL_17414598](https://platform.epicov.org/epi3/start/EPI_ISL/17414598) | Nigeria | 2021-Dec-28 | A/avian/Nigeria/745_22VIR3286-31/2021 | National Veterinary Research Institute | Istituto Zooprofilattico Sperimentale Delle Venezie | Meseko, C.; Milani, A.; Inuwa, B.; Chinyere, C.; Shittu, I.; Ahmed, J.; Giussani, E.; Palumbo, E.; Zecchin, B.; Bonfante, F.; Maniero, S.; Fusaro, A.; Gobbo, F.; Terregino, C.; Olasoju, T.; Monne, I.; Muhammad, M. |
| [EPI_ISL_17414597](https://platform.epicov.org/epi3/start/EPI_ISL/17414597) | Nigeria | 2021-Dec-28 | A/avian/Nigeria/741_22VIR3286-29/2021 | National Veterinary Research Institute | Istituto Zooprofilattico Sperimentale Delle Venezie | Meseko, C.; Milani, A.; Inuwa, B.; Chinyere, C.; Shittu, I.; Ahmed, J.; Giussani, E.; Palumbo, E.; Zecchin, B.; Bonfante, F.; Maniero, S.; Fusaro, A.; Gobbo, F.; Terregino, C.; Olasoju, T.; Monne, I.; Muhammad, M. |
| [EPI_ISL_17414596](https://platform.epicov.org/epi3/start/EPI_ISL/17414596) | Nigeria | 2021-Dec-24 | A/avian/Nigeria/739_22VIR3286-27/2021 | National Veterinary Research Institute | Istituto Zooprofilattico Sperimentale Delle Venezie | Meseko, C.; Milani, A.; Inuwa, B.; Chinyere, C.; Shittu, I.; Ahmed, J.; Giussani, E.; Palumbo, E.; Zecchin, B.; Bonfante, F.; Maniero, S.; Fusaro, A.; Gobbo, F.; Terregino, C.; Olasoju, T.; Monne, I.; Muhammad, M. |
| [EPI_ISL_17414595](https://platform.epicov.org/epi3/start/EPI_ISL/17414595) | Nigeria | 2021-Dec-24 | A/avian/Nigeria/738_22VIR3286-26/2021 | National Veterinary Research Institute | Istituto Zooprofilattico Sperimentale Delle Venezie | Meseko, C.; Milani, A.; Inuwa, B.; Chinyere, C.; Shittu, I.; Ahmed, J.; Giussani, E.; Palumbo, E.; Zecchin, B.; Bonfante, F.; Maniero, S.; Fusaro, A.; Gobbo, F.; Terregino, C.; Olasoju, T.; Monne, I.; Muhammad, M. |
| [EPI_ISL_17414594](https://platform.epicov.org/epi3/start/EPI_ISL/17414594) | Nigeria | 2021-Dec-24 | A/avian/Nigeria/737_22VIR3286-25/2021 | National Veterinary Research Institute | Istituto Zooprofilattico Sperimentale Delle Venezie | Meseko, C.; Milani, A.; Inuwa, B.; Chinyere, C.; Shittu, I.; Ahmed, J.; Giussani, E.; Palumbo, E.; Zecchin, B.; Bonfante, F.; Maniero, S.; Fusaro, A.; Gobbo, F.; Terregino, C.; Olasoju, T.; Monne, I.; Muhammad, M. |
| [EPI_ISL_17414593](https://platform.epicov.org/epi3/start/EPI_ISL/17414593) | Nigeria | 2021-Dec-23 | A/avian/Nigeria/721_22VIR3286-21/2021 | National Veterinary Research Institute | Istituto Zooprofilattico Sperimentale Delle Venezie | Meseko, C.; Milani, A.; Inuwa, B.; Chinyere, C.; Shittu, I.; Ahmed, J.; Giussani, E.; Palumbo, E.; Zecchin, B.; Bonfante, F.; Maniero, S.; Fusaro, A.; Gobbo, F.; Terregino, C.; Olasoju, T.; Monne, I.; Muhammad, M. |
| [EPI_ISL_17414592](https://platform.epicov.org/epi3/start/EPI_ISL/17414592) | Nigeria | 2021-Dec-15 | A/avian/Nigeria/711_22VIR3286-17/2021 | National Veterinary Research Institute | Istituto Zooprofilattico Sperimentale Delle Venezie | Meseko, C.; Milani, A.; Inuwa, B.; Chinyere, C.; Shittu, I.; Ahmed, J.; Giussani, E.; Palumbo, E.; Zecchin, B.; Bonfante, F.; Maniero, S.; Fusaro, A.; Gobbo, F.; Terregino, C.; Olasoju, T.; Monne, I.; Muhammad, M. |
| [EPI_ISL_17414591](https://platform.epicov.org/epi3/start/EPI_ISL/17414591) | Nigeria | 2021-Nov-19 | A/avian/Nigeria/684_22VIR3286-12/2021 | National Veterinary Research Institute | Istituto Zooprofilattico Sperimentale Delle Venezie | Meseko, C.; Milani, A.; Inuwa, B.; Chinyere, C.; Shittu, I.; Ahmed, J.; Giussani, E.; Palumbo, E.; Zecchin, B.; Bonfante, F.; Maniero, S.; Fusaro, A.; Gobbo, F.; Terregino, C.; Olasoju, T.; Monne, I.; Muhammad, M. |
| [EPI_ISL_17414590](https://platform.epicov.org/epi3/start/EPI_ISL/17414590) | Nigeria | 2021-Nov-19 | A/avian/Nigeria/683_22VIR3286-11/2021 | National Veterinary Research Institute | Istituto Zooprofilattico Sperimentale Delle Venezie | Meseko, C.; Milani, A.; Inuwa, B.; Chinyere, C.; Shittu, I.; Ahmed, J.; Giussani, E.; Palumbo, E.; Zecchin, B.; Bonfante, F.; Maniero, S.; Fusaro, A.; Gobbo, F.; Terregino, C.; Olasoju, T.; Monne, I.; Muhammad, M. |
| [EPI_ISL_17414589](https://platform.epicov.org/epi3/start/EPI_ISL/17414589) | Nigeria | 2021-Nov-11 | A/avian/Nigeria/674_22VIR3286-10/2021 | National Veterinary Research Institute | Istituto Zooprofilattico Sperimentale Delle Venezie | Meseko, C.; Milani, A.; Inuwa, B.; Chinyere, C.; Shittu, I.; Ahmed, J.; Giussani, E.; Palumbo, E.; Zecchin, B.; Bonfante, F.; Maniero, S.; Fusaro, A.; Gobbo, F.; Terregino, C.; Olasoju, T.; Monne, I.; Muhammad, M. |
| [EPI_ISL_17414588](https://platform.epicov.org/epi3/start/EPI_ISL/17414588) | Nigeria | 2021-Oct-28 | A/avian/Nigeria/658_22VIR3286-8/2021 | National Veterinary Research Institute | Istituto Zooprofilattico Sperimentale Delle Venezie | Meseko, C.; Milani, A.; Inuwa, B.; Chinyere, C.; Shittu, I.; Ahmed, J.; Giussani, E.; Palumbo, E.; Zecchin, B.; Bonfante, F.; Maniero, S.; Fusaro, A.; Gobbo, F.; Terregino, C.; Olasoju, T.; Monne, I.; Muhammad, M. |
| [EPI_ISL_17414587](https://platform.epicov.org/epi3/start/EPI_ISL/17414587) | Nigeria | 2021-Oct-16 | A/avian/Nigeria/643_22VIR3286-4/2021 | National Veterinary Research Institute | Istituto Zooprofilattico Sperimentale Delle Venezie | Meseko, C.; Milani, A.; Inuwa, B.; Chinyere, C.; Shittu, I.; Ahmed, J.; Giussani, E.; Palumbo, E.; Zecchin, B.; Bonfante, F.; Maniero, S.; Fusaro, A.; Gobbo, F.; Terregino, C.; Olasoju, T.; Monne, I.; Muhammad, M. |
| [EPI_ISL_17414586](https://platform.epicov.org/epi3/start/EPI_ISL/17414586) | Nigeria | 2021-Oct-05 | A/avian/Nigeria/599_22VIR3286-1/2021 | National Veterinary Research Institute | Istituto Zooprofilattico Sperimentale Delle Venezie | Meseko, C.; Milani, A.; Inuwa, B.; Chinyere, C.; Shittu, I.; Ahmed, J.; Giussani, E.; Palumbo, E.; Zecchin, B.; Bonfante, F.; Maniero, S.; Fusaro, A.; Gobbo, F.; Terregino, C.; Olasoju, T.; Monne, I.; Muhammad, M. |
| [EPI_ISL_17414585](https://platform.epicov.org/epi3/start/EPI_ISL/17414585) | Nigeria | 2022-Jan-28 | A/avian/Nigeria/120_22VIR3286-62/2022 | National Veterinary Research Institute | Istituto Zooprofilattico Sperimentale Delle Venezie | Meseko, C.; Milani, A.; Inuwa, B.; Chinyere, C.; Shittu, I.; Ahmed, J.; Giussani, E.; Palumbo, E.; Zecchin, B.; Bonfante, F.; Maniero, S.; Fusaro, A.; Gobbo, F.; Terregino, C.; Olasoju, T.; Monne, I.; Muhammad, M. |
| [EPI_ISL_17414584](https://platform.epicov.org/epi3/start/EPI_ISL/17414584) | Nigeria | 2022-Jan-28 | A/avian/Nigeria/115_22VIR3286-61/2022 | National Veterinary Research Institute | Istituto Zooprofilattico Sperimentale Delle Venezie | Meseko, C.; Milani, A.; Inuwa, B.; Chinyere, C.; Shittu, I.; Ahmed, J.; Giussani, E.; Palumbo, E.; Zecchin, B.; Bonfante, F.; Maniero, S.; Fusaro, A.; Gobbo, F.; Terregino, C.; Olasoju, T.; Monne, I.; Muhammad, M. |
| [EPI_ISL_17414583](https://platform.epicov.org/epi3/start/EPI_ISL/17414583) | Nigeria | 2022-Jan-07 | A/avian/Nigeria/023_22VIR3286-44/2022 | National Veterinary Research Institute | Istituto Zooprofilattico Sperimentale Delle Venezie | Meseko, C.; Milani, A.; Inuwa, B.; Chinyere, C.; Shittu, I.; Ahmed, J.; Giussani, E.; Palumbo, E.; Zecchin, B.; Bonfante, F.; Maniero, S.; Fusaro, A.; Gobbo, F.; Terregino, C.; Olasoju, T.; Monne, I.; Muhammad, M. |
| [EPI_ISL_17414582](https://platform.epicov.org/epi3/start/EPI_ISL/17414582) | Nigeria | 2022-Jan-04 | A/avian/Nigeria/004_22VIR3286-37/2022 | National Veterinary Research Institute | Istituto Zooprofilattico Sperimentale Delle Venezie | Meseko, C.; Milani, A.; Inuwa, B.; Chinyere, C.; Shittu, I.; Ahmed, J.; Giussani, E.; Palumbo, E.; Zecchin, B.; Bonfante, F.; Maniero, S.; Fusaro, A.; Gobbo, F.; Terregino, C.; Olasoju, T.; Monne, I.; Muhammad, M. |
| [EPI_ISL_17638498](https://platform.epicov.org/epi3/start/EPI_ISL/17638498) | Niger | 2023-Jan-23 | A/layer/Niger/40-23_23VIR3551-22/2023 | Laboratoire Central de l'Elevage (LABOCEL) | Istituto Zooprofilattico Sperimentale Delle Venezie | Souley, M. M.; Milani, A.; Yaou, B.; Amadou, H.; Haido, A. M.; Issiako, A.; Varotto, M.; Giussani, E.; Palumbo, E.; Zecchin, B.; Fusaro, A. |
| [EPI_ISL_13048382](https://platform.epicov.org/epi3/start/EPI_ISL/13048382) | Burkina Faso | 2021-Dec-07 | A/avian/Burkina_Faso/21VIR11911-3/2021 | Laboratoire National d’Elevage | Istituto Zooprofilattico Sperimentale Delle Venezie | LALIDIA-OUOBA, B.; LAMOUNI-ZERBO-OUERMI, H.; Zecchin, B.; Barbierato, G.; OUANDAOGO-SANDAOGO, H.; GUITTI-KINDO, M.; GUIGMA, D.; Barro, N., Palumbo, E.; Giussani, E.; Bortolami, A.; Terregino, C.; Fusaro, A.; Monne, I. |
| [EPI_ISL_13048381](https://platform.epicov.org/epi3/start/EPI_ISL/13048381) | Burkina Faso | 2021-Dec-08 | A/avian/Burkina_Faso/21VIR11911-1/2021 | Laboratoire National d’Elevage | Istituto Zooprofilattico Sperimentale Delle Venezie | LALIDIA-OUOBA, B.; LAMOUNI-ZERBO-OUERMI, H.; Zecchin, B.; Barbierato, G.; OUANDAOGO-SANDAOGO, H.; GUITTI-KINDO, M.; GUIGMA, D.; Barro, N., Palumbo, E.; Giussani, E.; Bortolami, A.; Terregino, C.; Fusaro, A.; Monne, I. |
| [EPI_ISL_12045918](https://platform.epicov.org/epi3/start/EPI_ISL/12045918) | Botswana | 2021-Aug-18 | A/Dove/Botswana/2097/2021 | Botswana National Veterinary Laboratory | Animal and Plant Health Agency (APHA) | na |
| [EPI_ISL_12045920](https://platform.epicov.org/epi3/start/EPI_ISL/12045920) | Botswana | 2021-Jun-03 | A/fish_eagle/Botswana/1338/2021 | Botswana National Veterinary Laboratory | Animal and Plant Health Agency (APHA) | na |
| [EPI_ISL_12045919](https://platform.epicov.org/epi3/start/EPI_ISL/12045919) | Botswana | 2021-Sep-13 | A/Dove/Botswana/2334/2021 | Botswana National Veterinary Laboratory | Animal and Plant Health Agency (APHA) | na |
| [EPI_ISL_11007547](https://platform.epicov.org/epi3/start/EPI_ISL/11007547) | Niger | 2022-Jan-01 | A/chicken/Niger/22VIR1409-9/2022 | Laboratoire Central de l’Elevage (LABOCEL) | Istituto Zooprofilattico Sperimentale delle Venezie | Souley, M.M.; Yaou, B.; Amadou, H.; Haido, A.M.; Issiako, A.; Alassane, A.; Barbierato, G.; Zecchin, B.; Fusaro, A.; Schivo, A.; Salviato, A.; Palumbo, E.; Giussani, E.; Monne, I.; Terregino, C. |
| [EPI_ISL_11007546](https://platform.epicov.org/epi3/start/EPI_ISL/11007546) | Niger | 2022-Jan-01 | A/chicken/Niger/22VIR1409-5/2022 | Laboratoire Central de l’Elevage (LABOCEL) | Istituto Zooprofilattico Sperimentale delle Venezie | Souley, M.M.; Yaou, B.; Amadou, H.; Haido, A.M.; Issiako, A.; Alassane, A.; Barbierato, G.; Zecchin, B.; Fusaro, A.; Schivo, A.; Salviato, A.; Palumbo, E.; Giussani, E.; Monne, I.; Terregino, C. |
| [EPI_ISL_11007543](https://platform.epicov.org/epi3/start/EPI_ISL/11007543) | Niger | 2022-Jan-01 | A/chicken/Niger/22VIR1409-23/2022 | Laboratoire Central de l’Elevage (LABOCEL) | Istituto Zooprofilattico Sperimentale delle Venezie | Souley, M.M.; Yaou, B.; Amadou, H.; Haido, A.M.; Issiako, A.; Alassane, A.; Barbierato, G.; Zecchin, B.; Fusaro, A.; Schivo, A.; Salviato, A.; Palumbo, E.; Giussani, E.; Monne, I.; Terregino, C. |
| [EPI_ISL_11007542](https://platform.epicov.org/epi3/start/EPI_ISL/11007542) | Niger | 2022-Jan-01 | A/chicken/Niger/22VIR1409-13/2022 | Laboratoire Central de l’Elevage (LABOCEL) | Istituto Zooprofilattico Sperimentale delle Venezie | Souley, M.M.; Yaou, B.; Amadou, H.; Haido, A.M.; Issiako, A.; Alassane, A.; Barbierato, G.; Zecchin, B.; Fusaro, A.; Schivo, A.; Salviato, A.; Palumbo, E.; Giussani, E.; Monne, I.; Terregino, C. |
| [EPI_ISL_15350905](https://platform.epicov.org/epi3/start/EPI_ISL/15350905) | Ghana | 2021-Jun-08 | A/chicken/Ghana/AVL-763_21VIR7050-39/2021 | Veterinary Services Directorate, Ministry of Food and Agriculture | Istituto Zooprofilattico Sperimentale delle Venezie | Odoom, T.; Ababio, P.T.; Danso, F.; Abakeh, P.; Youri, G.M.; Fia, G.E.; Yingar, D.T.; Daniel, B.; Arthur, D.; Kutame, M.S.; Barbierato, G.; Zecchin, B.; Fusaro, A.; Schivo, A.; Salviato, A.; Palumbo, E.; Giussani, E.; Pastori, A.; Monne, I.; Terregino, C. |
| [EPI_ISL_14769967](https://platform.epicov.org/epi3/start/EPI_ISL/14769967) | Lesotho | 2021-Jun-05 | A/chicken/Lesotho/352.3/2021 | na | na | Makalo,M.R.; Dundon,W.G.; Settypalli,T.B.; Datta,S.; Lamien,C.E.; Cattoli,G.; Phalatsi,M.S.; Lepheana,R.J.; Matlali,M.; Mahloane,R.G.; Molomo,M.; Mphaka,P.C. |
| [EPI_ISL_14769966](https://platform.epicov.org/epi3/start/EPI_ISL/14769966) | Lesotho | 2021-May-29 | A/chicken/Lesotho/341.10/2021 | na | na | Makalo,M.R.; Dundon,W.G.; Settypalli,T.B.; Datta,S.; Lamien,C.E.; Cattoli,G.; Phalatsi,M.S.; Lepheana,R.J.; Matlali,M.; Mahloane,R.G.; Molomo,M.; Mphaka,P.C. |
| [EPI_ISL_4061491](https://platform.epicov.org/epi3/start/EPI_ISL/4061491) | Nigeria | 2021-Feb-12 | A/chicken/Nigeria/VRD21-98_21VIR2288-6/2021 | na | na | Shittu,I.; Meseko,C.; Nwosuh,C.; Muhammad,M.; Alabi,O.; Tassoni,L.; Schivo,A.; Salviato,A.; Edoardo,G.; Zecchin,B.; Fusaro,A.; Monne,I. |
| [EPI_ISL_4061490](https://platform.epicov.org/epi3/start/EPI_ISL/4061490) | Nigeria | 2021-Feb-25 | A/chicken/Nigeria/VRD21-88_21VIR2288-8/2021 | na | na | Shittu,I.; Meseko,C.; Nwosuh,C.; Muhammad,M.; Alabi,O.; Tassoni,L.; Schivo,A.; Salviato,A.; Edoardo,G.; Zecchin,B.; Fusaro,A.; Monne,I. |
| [EPI_ISL_4061488](https://platform.epicov.org/epi3/start/EPI_ISL/4061488) | Nigeria | 2021-Feb-11 | A/chicken/Nigeria/VRD21-53B_21VIR2288-5/2021 | na | na | Shittu,I.; Meseko,C.; Nwosuh,C.; Muhammad,M.; Alabi,O.; Tassoni,L.; Schivo,A.; Salviato,A.; Edoardo,G.; Zecchin,B.; Fusaro,A.; Monne,I. |
| [EPI_ISL_4061486](https://platform.epicov.org/epi3/start/EPI_ISL/4061486) | Nigeria | 2021-Feb-05 | A/chicken/Nigeria/VRD21-37_21VIR2288-2/2021 | na | na | Shittu,I.; Meseko,C.; Nwosuh,C.; Muhammad,M.; Alabi,O.; Tassoni,L.; Schivo,A.; Salviato,A.; Edoardo,G.; Zecchin,B.; Fusaro,A.; Monne,I. |
| [EPI_ISL_4061485](https://platform.epicov.org/epi3/start/EPI_ISL/4061485) | Nigeria | 2021-Mar-01 | A/chicken/Nigeria/VRD21-109_21VIR2370-425/2021 | na | na | Shittu,I.; Meseko,C.; Nwosuh,C.; Muhammad,M.; Alabi,O.; Tassoni,L.; Schivo,A.; Salviato,A.; Edoardo,G.; Zecchin,B.; Fusaro,A.; Monne,I. |
| [EPI_ISL_4061484](https://platform.epicov.org/epi3/start/EPI_ISL/4061484) | Nigeria | 2021-Mar-01 | A/chicken/Nigeria/VRD21-102_21VIR2370-424/2021 | na | na | Shittu,I.; Meseko,C.; Nwosuh,C.; Muhammad,M.; Alabi,O.; Tassoni,L.; Schivo,A.; Salviato,A.; Edoardo,G.; Zecchin,B.; Fusaro,A.; Monne,I. |
| [EPI_ISL_4061483](https://platform.epicov.org/epi3/start/EPI_ISL/4061483) | Nigeria | 2021-Mar-01 | A/chicken/Nigeria/VRD21-100_21VIR2370-423/2021 | na | na | Shittu,I.; Meseko,C.; Nwosuh,C.; Muhammad,M.; Alabi,O.; Tassoni,L.; Schivo,A.; Salviato,A.; Edoardo,G.; Zecchin,B.; Fusaro,A.; Monne,I. |
| [EPI_ISL_4061482](https://platform.epicov.org/epi3/start/EPI_ISL/4061482) | Nigeria | 2021-Feb-05 | A/chicken/Nigeria/VRD21-035B_21VIR2288-1/2021 | na | na | Shittu,I.; Meseko,C.; Nwosuh,C.; Muhammad,M.; Alabi,O.; Tassoni,L.; Schivo,A.; Salviato,A.; Edoardo,G.; Zecchin,B.; Fusaro,A.; Monne,I. |
| [EPI_ISL_4061481](https://platform.epicov.org/epi3/start/EPI_ISL/4061481) | Nigeria | 2021-Feb-10 | A/chicken/Nigeria/VRD21-43_21VIR2288-4/2021 | na | na | Shittu,I.; Meseko,C.; Nwosuh,C.; Muhammad,M.; Alabi,O.; Tassoni,L.; Schivo,A.; Salviato,A.; Edoardo,G.; Zecchin,B.; Fusaro,A.; Monne,I. |
| [EPI_ISL_17414667](https://platform.epicov.org/epi3/start/EPI_ISL/17414667) | Nigeria | 2021-Dec-28 | A/chicken/Nigeria/743A_22VIR3286-80/2021 | National Veterinary Research Institute | Istituto Zooprofilattico Sperimentale Delle Venezie | Meseko, C.; Milani, A.; Inuwa, B.; Chinyere, C.; Shittu, I.; Ahmed, J.; Giussani, E.; Palumbo, E.; Zecchin, B.; Bonfante, F.; Maniero, S.; Fusaro, A.; Gobbo, F.; Terregino, C.; Olasoju, T.; Monne, I.; Muhammad, M. |
| [EPI_ISL_17414660](https://platform.epicov.org/epi3/start/EPI_ISL/17414660) | Nigeria | 2021-Feb-20 | A/chicken/Nigeria/VRD-21-82_21VIR2288-7/2021 | National Veterinary Research Institute | Istituto Zooprofilattico Sperimentale Delle Venezie | Meseko, C.; Milani, A.; Inuwa, B.; Chinyere, C.; Shittu, I.; Ahmed, J.; Giussani, E.; Palumbo, E.; Zecchin, B.; Bonfante, F.; Maniero, S.; Fusaro, A.; Gobbo, F.; Terregino, C.; Olasoju, T.; Monne, I.; Muhammad, M. |
| [EPI_ISL_17414659](https://platform.epicov.org/epi3/start/EPI_ISL/17414659) | Nigeria | 2021-Feb-05 | A/chicken/Nigeria/VRD-21-38_21VIR2288-3/2021 | National Veterinary Research Institute | Istituto Zooprofilattico Sperimentale Delle Venezie | Meseko, C.; Milani, A.; Inuwa, B.; Chinyere, C.; Shittu, I.; Ahmed, J.; Giussani, E.; Palumbo, E.; Zecchin, B.; Bonfante, F.; Maniero, S.; Fusaro, A.; Gobbo, F.; Terregino, C.; Olasoju, T.; Monne, I.; Muhammad, M. |
| [EPI_ISL_17414658](https://platform.epicov.org/epi3/start/EPI_ISL/17414658) | Nigeria | 2021-Jul-09 | A/chicken/Nigeria/VRD-21-361_21VIR7423-34/2021 | National Veterinary Research Institute | Istituto Zooprofilattico Sperimentale Delle Venezie | Meseko, C.; Milani, A.; Inuwa, B.; Chinyere, C.; Shittu, I.; Ahmed, J.; Giussani, E.; Palumbo, E.; Zecchin, B.; Bonfante, F.; Maniero, S.; Fusaro, A.; Gobbo, F.; Terregino, C.; Olasoju, T.; Monne, I.; Muhammad, M. |
| [EPI_ISL_17414657](https://platform.epicov.org/epi3/start/EPI_ISL/17414657) | Nigeria | 2021-Jun-30 | A/chicken/Nigeria/VRD-21-338_21VIR7423-29/2021 | National Veterinary Research Institute | Istituto Zooprofilattico Sperimentale Delle Venezie | Meseko, C.; Milani, A.; Inuwa, B.; Chinyere, C.; Shittu, I.; Ahmed, J.; Giussani, E.; Palumbo, E.; Zecchin, B.; Bonfante, F.; Maniero, S.; Fusaro, A.; Gobbo, F.; Terregino, C.; Olasoju, T.; Monne, I.; Muhammad, M. |
| [EPI_ISL_17414656](https://platform.epicov.org/epi3/start/EPI_ISL/17414656) | Nigeria | 2021-Jun-25 | A/chicken/Nigeria/VRD-21-334_21VIR7423-28/2021 | National Veterinary Research Institute | Istituto Zooprofilattico Sperimentale Delle Venezie | Meseko, C.; Milani, A.; Inuwa, B.; Chinyere, C.; Shittu, I.; Ahmed, J.; Giussani, E.; Palumbo, E.; Zecchin, B.; Bonfante, F.; Maniero, S.; Fusaro, A.; Gobbo, F.; Terregino, C.; Olasoju, T.; Monne, I.; Muhammad, M. |
| [EPI_ISL_17414655](https://platform.epicov.org/epi3/start/EPI_ISL/17414655) | Nigeria | 2021-Apr-03 | A/chicken/Nigeria/VRD-21-212_21VIR7423-5/2021 | National Veterinary Research Institute | Istituto Zooprofilattico Sperimentale Delle Venezie | Meseko, C.; Milani, A.; Inuwa, B.; Chinyere, C.; Shittu, I.; Ahmed, J.; Giussani, E.; Palumbo, E.; Zecchin, B.; Bonfante, F.; Maniero, S.; Fusaro, A.; Gobbo, F.; Terregino, C.; Olasoju, T.; Monne, I.; Muhammad, M. |
| [EPI_ISL_17414654](https://platform.epicov.org/epi3/start/EPI_ISL/17414654) | Nigeria | 2022-Jan-04 | A/chicken/Nigeria/751_22VIR3286-35/2022 | National Veterinary Research Institute | Istituto Zooprofilattico Sperimentale Delle Venezie | Meseko, C.; Milani, A.; Inuwa, B.; Chinyere, C.; Shittu, I.; Ahmed, J.; Giussani, E.; Palumbo, E.; Zecchin, B.; Bonfante, F.; Maniero, S.; Fusaro, A.; Gobbo, F.; Terregino, C.; Olasoju, T.; Monne, I.; Muhammad, M. |
| [EPI_ISL_17414653](https://platform.epicov.org/epi3/start/EPI_ISL/17414653) | Nigeria | 2021-Dec-29 | A/chicken/Nigeria/746_22VIR3286-32/2021 | National Veterinary Research Institute | Istituto Zooprofilattico Sperimentale Delle Venezie | Meseko, C.; Milani, A.; Inuwa, B.; Chinyere, C.; Shittu, I.; Ahmed, J.; Giussani, E.; Palumbo, E.; Zecchin, B.; Bonfante, F.; Maniero, S.; Fusaro, A.; Gobbo, F.; Terregino, C.; Olasoju, T.; Monne, I.; Muhammad, M. |
| [EPI_ISL_17414652](https://platform.epicov.org/epi3/start/EPI_ISL/17414652) | Nigeria | 2021-Dec-23 | A/chicken/Nigeria/732_22VIR3286-24/2021 | National Veterinary Research Institute | Istituto Zooprofilattico Sperimentale Delle Venezie | Meseko, C.; Milani, A.; Inuwa, B.; Chinyere, C.; Shittu, I.; Ahmed, J.; Giussani, E.; Palumbo, E.; Zecchin, B.; Bonfante, F.; Maniero, S.; Fusaro, A.; Gobbo, F.; Terregino, C.; Olasoju, T.; Monne, I.; Muhammad, M. |
| [EPI_ISL_17414651](https://platform.epicov.org/epi3/start/EPI_ISL/17414651) | Nigeria | 2021-Dec-22 | A/chicken/Nigeria/725_22VIR3286-22/2021 | National Veterinary Research Institute | Istituto Zooprofilattico Sperimentale Delle Venezie | Meseko, C.; Milani, A.; Inuwa, B.; Chinyere, C.; Shittu, I.; Ahmed, J.; Giussani, E.; Palumbo, E.; Zecchin, B.; Bonfante, F.; Maniero, S.; Fusaro, A.; Gobbo, F.; Terregino, C.; Olasoju, T.; Monne, I.; Muhammad, M. |
| [EPI_ISL_17414650](https://platform.epicov.org/epi3/start/EPI_ISL/17414650) | Nigeria | 2021-Dec-17 | A/chicken/Nigeria/720_22VIR3286-20/2021 | National Veterinary Research Institute | Istituto Zooprofilattico Sperimentale Delle Venezie | Meseko, C.; Milani, A.; Inuwa, B.; Chinyere, C.; Shittu, I.; Ahmed, J.; Giussani, E.; Palumbo, E.; Zecchin, B.; Bonfante, F.; Maniero, S.; Fusaro, A.; Gobbo, F.; Terregino, C.; Olasoju, T.; Monne, I.; Muhammad, M. |
| [EPI_ISL_17414649](https://platform.epicov.org/epi3/start/EPI_ISL/17414649) | Nigeria | 2021-Dec-16 | A/chicken/Nigeria/717_22VIR3286-19/2021 | National Veterinary Research Institute | Istituto Zooprofilattico Sperimentale Delle Venezie | Meseko, C.; Milani, A.; Inuwa, B.; Chinyere, C.; Shittu, I.; Ahmed, J.; Giussani, E.; Palumbo, E.; Zecchin, B.; Bonfante, F.; Maniero, S.; Fusaro, A.; Gobbo, F.; Terregino, C.; Olasoju, T.; Monne, I.; Muhammad, M. |
| [EPI_ISL_17414648](https://platform.epicov.org/epi3/start/EPI_ISL/17414648) | Nigeria | 2021-Dec-16 | A/chicken/Nigeria/716_22VIR3286-18/2021 | National Veterinary Research Institute | Istituto Zooprofilattico Sperimentale Delle Venezie | Meseko, C.; Milani, A.; Inuwa, B.; Chinyere, C.; Shittu, I.; Ahmed, J.; Giussani, E.; Palumbo, E.; Zecchin, B.; Bonfante, F.; Maniero, S.; Fusaro, A.; Gobbo, F.; Terregino, C.; Olasoju, T.; Monne, I.; Muhammad, M. |
| [EPI_ISL_17414647](https://platform.epicov.org/epi3/start/EPI_ISL/17414647) | Nigeria | 2021-Dec-06 | A/chicken/Nigeria/709_22VIR3286-15/2021 | National Veterinary Research Institute | Istituto Zooprofilattico Sperimentale Delle Venezie | Meseko, C.; Milani, A.; Inuwa, B.; Chinyere, C.; Shittu, I.; Ahmed, J.; Giussani, E.; Palumbo, E.; Zecchin, B.; Bonfante, F.; Maniero, S.; Fusaro, A.; Gobbo, F.; Terregino, C.; Olasoju, T.; Monne, I.; Muhammad, M. |
| [EPI_ISL_17414646](https://platform.epicov.org/epi3/start/EPI_ISL/17414646) | Nigeria | 2021-Nov-30 | A/chicken/Nigeria/701C_22VIR3286-14/2021 | National Veterinary Research Institute | Istituto Zooprofilattico Sperimentale Delle Venezie | Meseko, C.; Milani, A.; Inuwa, B.; Chinyere, C.; Shittu, I.; Ahmed, J.; Giussani, E.; Palumbo, E.; Zecchin, B.; Bonfante, F.; Maniero, S.; Fusaro, A.; Gobbo, F.; Terregino, C.; Olasoju, T.; Monne, I.; Muhammad, M. |
| [EPI_ISL_17414645](https://platform.epicov.org/epi3/start/EPI_ISL/17414645) | Nigeria | 2021-Nov-30 | A/chicken/Nigeria/698_22VIR3286-13/2021 | National Veterinary Research Institute | Istituto Zooprofilattico Sperimentale Delle Venezie | Meseko, C.; Milani, A.; Inuwa, B.; Chinyere, C.; Shittu, I.; Ahmed, J.; Giussani, E.; Palumbo, E.; Zecchin, B.; Bonfante, F.; Maniero, S.; Fusaro, A.; Gobbo, F.; Terregino, C.; Olasoju, T.; Monne, I.; Muhammad, M. |
| [EPI_ISL_17414644](https://platform.epicov.org/epi3/start/EPI_ISL/17414644) | Nigeria | 2021-Dec-20 | A/chicken/Nigeria/686-H5B04_22VIR3286-16/2021 | National Veterinary Research Institute | Istituto Zooprofilattico Sperimentale Delle Venezie | Meseko, C.; Milani, A.; Inuwa, B.; Chinyere, C.; Shittu, I.; Ahmed, J.; Giussani, E.; Palumbo, E.; Zecchin, B.; Bonfante, F.; Maniero, S.; Fusaro, A.; Gobbo, F.; Terregino, C.; Olasoju, T.; Monne, I.; Muhammad, M. |
| [EPI_ISL_17414643](https://platform.epicov.org/epi3/start/EPI_ISL/17414643) | Nigeria | 2021-Nov-05 | A/chicken/Nigeria/664_22VIR3286-9/2021 | National Veterinary Research Institute | Istituto Zooprofilattico Sperimentale Delle Venezie | Meseko, C.; Milani, A.; Inuwa, B.; Chinyere, C.; Shittu, I.; Ahmed, J.; Giussani, E.; Palumbo, E.; Zecchin, B.; Bonfante, F.; Maniero, S.; Fusaro, A.; Gobbo, F.; Terregino, C.; Olasoju, T.; Monne, I.; Muhammad, M. |
| [EPI_ISL_17414642](https://platform.epicov.org/epi3/start/EPI_ISL/17414642) | Nigeria | 2021-Oct-26 | A/chicken/Nigeria/653_22VIR3286-6/2021 | National Veterinary Research Institute | Istituto Zooprofilattico Sperimentale Delle Venezie | Meseko, C.; Milani, A.; Inuwa, B.; Chinyere, C.; Shittu, I.; Ahmed, J.; Giussani, E.; Palumbo, E.; Zecchin, B.; Bonfante, F.; Maniero, S.; Fusaro, A.; Gobbo, F.; Terregino, C.; Olasoju, T.; Monne, I.; Muhammad, M. |
| [EPI_ISL_17414641](https://platform.epicov.org/epi3/start/EPI_ISL/17414641) | Nigeria | 2021-Oct-26 | A/chicken/Nigeria/648_22VIR3286-5/2021 | National Veterinary Research Institute | Istituto Zooprofilattico Sperimentale Delle Venezie | Meseko, C.; Milani, A.; Inuwa, B.; Chinyere, C.; Shittu, I.; Ahmed, J.; Giussani, E.; Palumbo, E.; Zecchin, B.; Bonfante, F.; Maniero, S.; Fusaro, A.; Gobbo, F.; Terregino, C.; Olasoju, T.; Monne, I.; Muhammad, M. |
| [EPI_ISL_17414640](https://platform.epicov.org/epi3/start/EPI_ISL/17414640) | Nigeria | 2021-Oct-16 | A/chicken/Nigeria/637_22VIR3286-3/2021 | National Veterinary Research Institute | Istituto Zooprofilattico Sperimentale Delle Venezie | Meseko, C.; Milani, A.; Inuwa, B.; Chinyere, C.; Shittu, I.; Ahmed, J.; Giussani, E.; Palumbo, E.; Zecchin, B.; Bonfante, F.; Maniero, S.; Fusaro, A.; Gobbo, F.; Terregino, C.; Olasoju, T.; Monne, I.; Muhammad, M. |
| [EPI_ISL_17414639](https://platform.epicov.org/epi3/start/EPI_ISL/17414639) | Nigeria | 2021-Oct-05 | A/chicken/Nigeria/601_22VIR3286-2/2021 | National Veterinary Research Institute | Istituto Zooprofilattico Sperimentale Delle Venezie | Meseko, C.; Milani, A.; Inuwa, B.; Chinyere, C.; Shittu, I.; Ahmed, J.; Giussani, E.; Palumbo, E.; Zecchin, B.; Bonfante, F.; Maniero, S.; Fusaro, A.; Gobbo, F.; Terregino, C.; Olasoju, T.; Monne, I.; Muhammad, M. |
| [EPI_ISL_17414638](https://platform.epicov.org/epi3/start/EPI_ISL/17414638) | Nigeria | 2022-Feb-04 | A/chicken/Nigeria/164A_22VIR3286-69/2022 | National Veterinary Research Institute | Istituto Zooprofilattico Sperimentale Delle Venezie | Meseko, C.; Milani, A.; Inuwa, B.; Chinyere, C.; Shittu, I.; Ahmed, J.; Giussani, E.; Palumbo, E.; Zecchin, B.; Bonfante, F.; Maniero, S.; Fusaro, A.; Gobbo, F.; Terregino, C.; Olasoju, T.; Monne, I.; Muhammad, M. |
| [EPI_ISL_17414637](https://platform.epicov.org/epi3/start/EPI_ISL/17414637) | Nigeria | 2022-Feb-03 | A/chicken/Nigeria/157_22VIR3286-68/2022 | National Veterinary Research Institute | Istituto Zooprofilattico Sperimentale Delle Venezie | Meseko, C.; Milani, A.; Inuwa, B.; Chinyere, C.; Shittu, I.; Ahmed, J.; Giussani, E.; Palumbo, E.; Zecchin, B.; Bonfante, F.; Maniero, S.; Fusaro, A.; Gobbo, F.; Terregino, C.; Olasoju, T.; Monne, I.; Muhammad, M. |
| [EPI_ISL_17414636](https://platform.epicov.org/epi3/start/EPI_ISL/17414636) | Nigeria | 2022-Feb-02 | A/chicken/Nigeria/149_22VIR3286-67/2022 | National Veterinary Research Institute | Istituto Zooprofilattico Sperimentale Delle Venezie | Meseko, C.; Milani, A.; Inuwa, B.; Chinyere, C.; Shittu, I.; Ahmed, J.; Giussani, E.; Palumbo, E.; Zecchin, B.; Bonfante, F.; Maniero, S.; Fusaro, A.; Gobbo, F.; Terregino, C.; Olasoju, T.; Monne, I.; Muhammad, M. |
| [EPI_ISL_17414635](https://platform.epicov.org/epi3/start/EPI_ISL/17414635) | Nigeria | 2022-Feb-01 | A/chicken/Nigeria/146_22VIR3286-66/2022 | National Veterinary Research Institute | Istituto Zooprofilattico Sperimentale Delle Venezie | Meseko, C.; Milani, A.; Inuwa, B.; Chinyere, C.; Shittu, I.; Ahmed, J.; Giussani, E.; Palumbo, E.; Zecchin, B.; Bonfante, F.; Maniero, S.; Fusaro, A.; Gobbo, F.; Terregino, C.; Olasoju, T.; Monne, I.; Muhammad, M. |
| [EPI_ISL_17414634](https://platform.epicov.org/epi3/start/EPI_ISL/17414634) | Nigeria | 2022-Feb-01 | A/chicken/Nigeria/141B_22VIR3286-65/2022 | National Veterinary Research Institute | Istituto Zooprofilattico Sperimentale Delle Venezie | Meseko, C.; Milani, A.; Inuwa, B.; Chinyere, C.; Shittu, I.; Ahmed, J.; Giussani, E.; Palumbo, E.; Zecchin, B.; Bonfante, F.; Maniero, S.; Fusaro, A.; Gobbo, F.; Terregino, C.; Olasoju, T.; Monne, I.; Muhammad, M. |
| [EPI_ISL_17414633](https://platform.epicov.org/epi3/start/EPI_ISL/17414633) | Nigeria | 2022-Jan-30 | A/chicken/Nigeria/131_22VIR3286-64/2022 | National Veterinary Research Institute | Istituto Zooprofilattico Sperimentale Delle Venezie | Meseko, C.; Milani, A.; Inuwa, B.; Chinyere, C.; Shittu, I.; Ahmed, J.; Giussani, E.; Palumbo, E.; Zecchin, B.; Bonfante, F.; Maniero, S.; Fusaro, A.; Gobbo, F.; Terregino, C.; Olasoju, T.; Monne, I.; Muhammad, M. |
| [EPI_ISL_17414632](https://platform.epicov.org/epi3/start/EPI_ISL/17414632) | Nigeria | 2022-Jan-28 | A/chicken/Nigeria/128_22VIR3286-63/2022 | National Veterinary Research Institute | Istituto Zooprofilattico Sperimentale Delle Venezie | Meseko, C.; Milani, A.; Inuwa, B.; Chinyere, C.; Shittu, I.; Ahmed, J.; Giussani, E.; Palumbo, E.; Zecchin, B.; Bonfante, F.; Maniero, S.; Fusaro, A.; Gobbo, F.; Terregino, C.; Olasoju, T.; Monne, I.; Muhammad, M. |
| [EPI_ISL_17414631](https://platform.epicov.org/epi3/start/EPI_ISL/17414631) | Nigeria | 2022-Jan-26 | A/chicken/Nigeria/095_22VIR3286-60/2022 | National Veterinary Research Institute | Istituto Zooprofilattico Sperimentale Delle Venezie | Meseko, C.; Milani, A.; Inuwa, B.; Chinyere, C.; Shittu, I.; Ahmed, J.; Giussani, E.; Palumbo, E.; Zecchin, B.; Bonfante, F.; Maniero, S.; Fusaro, A.; Gobbo, F.; Terregino, C.; Olasoju, T.; Monne, I.; Muhammad, M. |
| [EPI_ISL_17414630](https://platform.epicov.org/epi3/start/EPI_ISL/17414630) | Nigeria | 2022-Jan-26 | A/chicken/Nigeria/086_22VIR3286-59/2022 | National Veterinary Research Institute | Istituto Zooprofilattico Sperimentale Delle Venezie | Meseko, C.; Milani, A.; Inuwa, B.; Chinyere, C.; Shittu, I.; Ahmed, J.; Giussani, E.; Palumbo, E.; Zecchin, B.; Bonfante, F.; Maniero, S.; Fusaro, A.; Gobbo, F.; Terregino, C.; Olasoju, T.; Monne, I.; Muhammad, M. |
| [EPI_ISL_17414629](https://platform.epicov.org/epi3/start/EPI_ISL/17414629) | Nigeria | 2022-Jan-24 | A/chicken/Nigeria/082_22VIR3286-58/2022 | National Veterinary Research Institute | Istituto Zooprofilattico Sperimentale Delle Venezie | Meseko, C.; Milani, A.; Inuwa, B.; Chinyere, C.; Shittu, I.; Ahmed, J.; Giussani, E.; Palumbo, E.; Zecchin, B.; Bonfante, F.; Maniero, S.; Fusaro, A.; Gobbo, F.; Terregino, C.; Olasoju, T.; Monne, I.; Muhammad, M. |
| [EPI_ISL_17414628](https://platform.epicov.org/epi3/start/EPI_ISL/17414628) | Nigeria | 2022-Jan-22 | A/chicken/Nigeria/078_22VIR3286-57/2022 | National Veterinary Research Institute | Istituto Zooprofilattico Sperimentale Delle Venezie | Meseko, C.; Milani, A.; Inuwa, B.; Chinyere, C.; Shittu, I.; Ahmed, J.; Giussani, E.; Palumbo, E.; Zecchin, B.; Bonfante, F.; Maniero, S.; Fusaro, A.; Gobbo, F.; Terregino, C.; Olasoju, T.; Monne, I.; Muhammad, M. |
| [EPI_ISL_17414627](https://platform.epicov.org/epi3/start/EPI_ISL/17414627) | Nigeria | 2022-Jan-21 | A/chicken/Nigeria/068_22VIR3286-56/2022 | National Veterinary Research Institute | Istituto Zooprofilattico Sperimentale Delle Venezie | Meseko, C.; Milani, A.; Inuwa, B.; Chinyere, C.; Shittu, I.; Ahmed, J.; Giussani, E.; Palumbo, E.; Zecchin, B.; Bonfante, F.; Maniero, S.; Fusaro, A.; Gobbo, F.; Terregino, C.; Olasoju, T.; Monne, I.; Muhammad, M. |
| [EPI_ISL_17414626](https://platform.epicov.org/epi3/start/EPI_ISL/17414626) | Nigeria | 2022-Jan-21 | A/chicken/Nigeria/064_22VIR3286-55/2022 | National Veterinary Research Institute | Istituto Zooprofilattico Sperimentale Delle Venezie | Meseko, C.; Milani, A.; Inuwa, B.; Chinyere, C.; Shittu, I.; Ahmed, J.; Giussani, E.; Palumbo, E.; Zecchin, B.; Bonfante, F.; Maniero, S.; Fusaro, A.; Gobbo, F.; Terregino, C.; Olasoju, T.; Monne, I.; Muhammad, M. |
| [EPI_ISL_17414625](https://platform.epicov.org/epi3/start/EPI_ISL/17414625) | Nigeria | 2022-Jan-17 | A/chicken/Nigeria/049_22VIR3286-53/2022 | National Veterinary Research Institute | Istituto Zooprofilattico Sperimentale Delle Venezie | Meseko, C.; Milani, A.; Inuwa, B.; Chinyere, C.; Shittu, I.; Ahmed, J.; Giussani, E.; Palumbo, E.; Zecchin, B.; Bonfante, F.; Maniero, S.; Fusaro, A.; Gobbo, F.; Terregino, C.; Olasoju, T.; Monne, I.; Muhammad, M. |
| [EPI_ISL_17414624](https://platform.epicov.org/epi3/start/EPI_ISL/17414624) | Nigeria | 2022-Jan-17 | A/chicken/Nigeria/048_22VIR3286-52/2022 | National Veterinary Research Institute | Istituto Zooprofilattico Sperimentale Delle Venezie | Meseko, C.; Milani, A.; Inuwa, B.; Chinyere, C.; Shittu, I.; Ahmed, J.; Giussani, E.; Palumbo, E.; Zecchin, B.; Bonfante, F.; Maniero, S.; Fusaro, A.; Gobbo, F.; Terregino, C.; Olasoju, T.; Monne, I.; Muhammad, M. |
| [EPI_ISL_17414623](https://platform.epicov.org/epi3/start/EPI_ISL/17414623) | Nigeria | 2022-Jan-15 | A/chicken/Nigeria/042_22VIR3286-51/2022 | National Veterinary Research Institute | Istituto Zooprofilattico Sperimentale Delle Venezie | Meseko, C.; Milani, A.; Inuwa, B.; Chinyere, C.; Shittu, I.; Ahmed, J.; Giussani, E.; Palumbo, E.; Zecchin, B.; Bonfante, F.; Maniero, S.; Fusaro, A.; Gobbo, F.; Terregino, C.; Olasoju, T.; Monne, I.; Muhammad, M. |
| [EPI_ISL_17414622](https://platform.epicov.org/epi3/start/EPI_ISL/17414622) | Nigeria | 2022-Jan-13 | A/chicken/Nigeria/040_22VIR3286-50/2022 | National Veterinary Research Institute | Istituto Zooprofilattico Sperimentale Delle Venezie | Meseko, C.; Milani, A.; Inuwa, B.; Chinyere, C.; Shittu, I.; Ahmed, J.; Giussani, E.; Palumbo, E.; Zecchin, B.; Bonfante, F.; Maniero, S.; Fusaro, A.; Gobbo, F.; Terregino, C.; Olasoju, T.; Monne, I.; Muhammad, M. |
| [EPI_ISL_17414621](https://platform.epicov.org/epi3/start/EPI_ISL/17414621) | Nigeria | 2022-Jan-13 | A/chicken/Nigeria/037_22VIR3286-49/2022 | National Veterinary Research Institute | Istituto Zooprofilattico Sperimentale Delle Venezie | Meseko, C.; Milani, A.; Inuwa, B.; Chinyere, C.; Shittu, I.; Ahmed, J.; Giussani, E.; Palumbo, E.; Zecchin, B.; Bonfante, F.; Maniero, S.; Fusaro, A.; Gobbo, F.; Terregino, C.; Olasoju, T.; Monne, I.; Muhammad, M. |
| [EPI_ISL_17414620](https://platform.epicov.org/epi3/start/EPI_ISL/17414620) | Nigeria | 2022-Jan-12 | A/chicken/Nigeria/034_22VIR3286-48/2022 | National Veterinary Research Institute | Istituto Zooprofilattico Sperimentale Delle Venezie | Meseko, C.; Milani, A.; Inuwa, B.; Chinyere, C.; Shittu, I.; Ahmed, J.; Giussani, E.; Palumbo, E.; Zecchin, B.; Bonfante, F.; Maniero, S.; Fusaro, A.; Gobbo, F.; Terregino, C.; Olasoju, T.; Monne, I.; Muhammad, M. |
| [EPI_ISL_17414619](https://platform.epicov.org/epi3/start/EPI_ISL/17414619) | Nigeria | 2022-Jan-12 | A/chicken/Nigeria/030_22VIR3286-47/2022 | National Veterinary Research Institute | Istituto Zooprofilattico Sperimentale Delle Venezie | Meseko, C.; Milani, A.; Inuwa, B.; Chinyere, C.; Shittu, I.; Ahmed, J.; Giussani, E.; Palumbo, E.; Zecchin, B.; Bonfante, F.; Maniero, S.; Fusaro, A.; Gobbo, F.; Terregino, C.; Olasoju, T.; Monne, I.; Muhammad, M. |
| [EPI_ISL_17414618](https://platform.epicov.org/epi3/start/EPI_ISL/17414618) | Nigeria | 2022-Jan-10 | A/chicken/Nigeria/026_22VIR3286-46/2022 | National Veterinary Research Institute | Istituto Zooprofilattico Sperimentale Delle Venezie | Meseko, C.; Milani, A.; Inuwa, B.; Chinyere, C.; Shittu, I.; Ahmed, J.; Giussani, E.; Palumbo, E.; Zecchin, B.; Bonfante, F.; Maniero, S.; Fusaro, A.; Gobbo, F.; Terregino, C.; Olasoju, T.; Monne, I.; Muhammad, M. |
| [EPI_ISL_17414617](https://platform.epicov.org/epi3/start/EPI_ISL/17414617) | Nigeria | 2022-Jan-07 | A/chicken/Nigeria/024_22VIR3286-45/2022 | National Veterinary Research Institute | Istituto Zooprofilattico Sperimentale Delle Venezie | Meseko, C.; Milani, A.; Inuwa, B.; Chinyere, C.; Shittu, I.; Ahmed, J.; Giussani, E.; Palumbo, E.; Zecchin, B.; Bonfante, F.; Maniero, S.; Fusaro, A.; Gobbo, F.; Terregino, C.; Olasoju, T.; Monne, I.; Muhammad, M. |
| [EPI_ISL_17414616](https://platform.epicov.org/epi3/start/EPI_ISL/17414616) | Nigeria | 2022-Jan-07 | A/chicken/Nigeria/020_22VIR3286-43/2022 | National Veterinary Research Institute | Istituto Zooprofilattico Sperimentale Delle Venezie | Meseko, C.; Milani, A.; Inuwa, B.; Chinyere, C.; Shittu, I.; Ahmed, J.; Giussani, E.; Palumbo, E.; Zecchin, B.; Bonfante, F.; Maniero, S.; Fusaro, A.; Gobbo, F.; Terregino, C.; Olasoju, T.; Monne, I.; Muhammad, M. |
| [EPI_ISL_17414615](https://platform.epicov.org/epi3/start/EPI_ISL/17414615) | Nigeria | 2022-Jan-07 | A/chicken/Nigeria/018_22VIR3286-42/2022 | National Veterinary Research Institute | Istituto Zooprofilattico Sperimentale Delle Venezie | Meseko, C.; Milani, A.; Inuwa, B.; Chinyere, C.; Shittu, I.; Ahmed, J.; Giussani, E.; Palumbo, E.; Zecchin, B.; Bonfante, F.; Maniero, S.; Fusaro, A.; Gobbo, F.; Terregino, C.; Olasoju, T.; Monne, I.; Muhammad, M. |
| [EPI_ISL_17414614](https://platform.epicov.org/epi3/start/EPI_ISL/17414614) | Nigeria | 2022-Jan-07 | A/chicken/Nigeria/014_22VIR3286-41/2022 | National Veterinary Research Institute | Istituto Zooprofilattico Sperimentale Delle Venezie | Meseko, C.; Milani, A.; Inuwa, B.; Chinyere, C.; Shittu, I.; Ahmed, J.; Giussani, E.; Palumbo, E.; Zecchin, B.; Bonfante, F.; Maniero, S.; Fusaro, A.; Gobbo, F.; Terregino, C.; Olasoju, T.; Monne, I.; Muhammad, M. |
| [EPI_ISL_17414613](https://platform.epicov.org/epi3/start/EPI_ISL/17414613) | Nigeria | 2022-Jan-07 | A/chicken/Nigeria/011_22VIR3286-40/2022 | National Veterinary Research Institute | Istituto Zooprofilattico Sperimentale Delle Venezie | Meseko, C.; Milani, A.; Inuwa, B.; Chinyere, C.; Shittu, I.; Ahmed, J.; Giussani, E.; Palumbo, E.; Zecchin, B.; Bonfante, F.; Maniero, S.; Fusaro, A.; Gobbo, F.; Terregino, C.; Olasoju, T.; Monne, I.; Muhammad, M. |
| [EPI_ISL_17414612](https://platform.epicov.org/epi3/start/EPI_ISL/17414612) | Nigeria | 2022-Jan-05 | A/chicken/Nigeria/009_22VIR3286-39/2022 | National Veterinary Research Institute | Istituto Zooprofilattico Sperimentale Delle Venezie | Meseko, C.; Milani, A.; Inuwa, B.; Chinyere, C.; Shittu, I.; Ahmed, J.; Giussani, E.; Palumbo, E.; Zecchin, B.; Bonfante, F.; Maniero, S.; Fusaro, A.; Gobbo, F.; Terregino, C.; Olasoju, T.; Monne, I.; Muhammad, M. |
| [EPI_ISL_17414611](https://platform.epicov.org/epi3/start/EPI_ISL/17414611) | Nigeria | 2022-Jan-04 | A/chicken/Nigeria/001_22VIR3286-36/2022 | National Veterinary Research Institute | Istituto Zooprofilattico Sperimentale Delle Venezie | Meseko, C.; Milani, A.; Inuwa, B.; Chinyere, C.; Shittu, I.; Ahmed, J.; Giussani, E.; Palumbo, E.; Zecchin, B.; Bonfante, F.; Maniero, S.; Fusaro, A.; Gobbo, F.; Terregino, C.; Olasoju, T.; Monne, I.; Muhammad, M. |
| [EPI_ISL_17638504](https://platform.epicov.org/epi3/start/EPI_ISL/17638504) | Niger | 2023-Feb-10 | A/chicken/Niger/67-23_23VIR3551-36/2023 | Laboratoire Central de l'Elevage (LABOCEL) | Istituto Zooprofilattico Sperimentale Delle Venezie | Souley, M. M.; Milani, A.; Yaou, B.; Amadou, H.; Haido, A. M.; Issiako, A.; Varotto, M.; Giussani, E.; Palumbo, E.; Zecchin, B.; Fusaro, A. |
| [EPI_ISL_17638502](https://platform.epicov.org/epi3/start/EPI_ISL/17638502) | Niger | 2023-Feb-02 | A/chicken/Niger/51-23_23VIR3551-32/2023 | Laboratoire Central de l'Elevage (LABOCEL) | Istituto Zooprofilattico Sperimentale Delle Venezie | Souley, M. M.; Milani, A.; Yaou, B.; Amadou, H.; Haido, A. M.; Issiako, A.; Varotto, M.; Giussani, E.; Palumbo, E.; Zecchin, B.; Fusaro, A. |
| [EPI_ISL_17638501](https://platform.epicov.org/epi3/start/EPI_ISL/17638501) | Niger | 2023-Feb-02 | A/chicken/Niger/51-23_23VIR3551-29/2023 | Laboratoire Central de l'Elevage (LABOCEL) | Istituto Zooprofilattico Sperimentale Delle Venezie | Souley, M. M.; Milani, A.; Yaou, B.; Amadou, H.; Haido, A. M.; Issiako, A.; Varotto, M.; Giussani, E.; Palumbo, E.; Zecchin, B.; Fusaro, A. |
| [EPI_ISL_17638500](https://platform.epicov.org/epi3/start/EPI_ISL/17638500) | Niger | 2023-Jan-28 | A/chicken/Niger/43-23_23VIR3551-25/2023 | Laboratoire Central de l'Elevage (LABOCEL) | Istituto Zooprofilattico Sperimentale Delle Venezie | Souley, M. M.; Milani, A.; Yaou, B.; Amadou, H.; Haido, A. M.; Issiako, A.; Varotto, M.; Giussani, E.; Palumbo, E.; Zecchin, B.; Fusaro, A. |
| [EPI_ISL_17638496](https://platform.epicov.org/epi3/start/EPI_ISL/17638496) | Niger | 2023-Jan-17 | A/chicken/Niger/22-23_23VIR3551-19/2023 | Laboratoire Central de l'Elevage (LABOCEL) | Istituto Zooprofilattico Sperimentale Delle Venezie | Souley, M. M.; Milani, A.; Yaou, B.; Amadou, H.; Haido, A. M.; Issiako, A.; Varotto, M.; Giussani, E.; Palumbo, E.; Zecchin, B.; Fusaro, A. |
| [EPI_ISL_17638493](https://platform.epicov.org/epi3/start/EPI_ISL/17638493) | Niger | 2023-Jan-12 | A/chicken/Niger/12-23_23VIR3551-8/2023 | Laboratoire Central de l'Elevage (LABOCEL) | Istituto Zooprofilattico Sperimentale Delle Venezie | Souley, M. M.; Milani, A.; Yaou, B.; Amadou, H.; Haido, A. M.; Issiako, A.; Varotto, M.; Giussani, E.; Palumbo, E.; Zecchin, B.; Fusaro, A. |
| [EPI_ISL_17638492](https://platform.epicov.org/epi3/start/EPI_ISL/17638492) | Niger | 2022-Dec-18 | A/chicken/Niger/288-22_23VIR3551-5/2022 | Laboratoire Central de l'Elevage (LABOCEL) | Istituto Zooprofilattico Sperimentale Delle Venezie | Souley, M. M.; Milani, A.; Yaou, B.; Amadou, H.; Haido, A. M.; Issiako, A.; Varotto, M.; Giussani, E.; Palumbo, E.; Zecchin, B.; Fusaro, A. |
| [EPI_ISL_17638491](https://platform.epicov.org/epi3/start/EPI_ISL/17638491) | Niger | 2022-Dec-14 | A/chicken/Niger/285-22_23VIR3551-2/2022 | Laboratoire Central de l'Elevage (LABOCEL) | Istituto Zooprofilattico Sperimentale Delle Venezie | Souley, M. M.; Milani, A.; Yaou, B.; Amadou, H.; Haido, A. M.; Issiako, A.; Varotto, M.; Giussani, E.; Palumbo, E.; Zecchin, B.; Fusaro, A. |
| [EPI_ISL_16997921](https://platform.epicov.org/epi3/start/EPI_ISL/16997921) | Ghana | 2021-Jun-08 | A/chicken/Ghana/AVL-763_21VIR7050-39/2021 | Veterinary Services Directorate, Ministry of Food and Agriculture | Istituto Zooprofilattico Sperimentale Delle Venezie | Odoom,T.; Ababio,P.T.; Danso,F.; Abakeh,P.; Youri,G.M.; Fia,G.E.; Yingar,D.T.; Daniel,B.; Arthur,D.; Kutame,M.S.; Barbierato,G.; Zecchin,B.; Fusaro,A.; Schivo,A.; Salviato,A.; Palumbo,E.; Giussani,E.; Pastori,A.; Monne,I.; Terregino,C. |
| [EPI_ISL_2276070](https://platform.epicov.org/epi3/start/EPI_ISL/2276070) | Senegal | 2020-Dec-23 | A/chicken/Senegal/21VIR1084-5/2021 | Istituto Zooprofilattico Sperimentale delle Venezie, EU/OIE/Reference Laboratory and FAO Reference Centre for AI and ND | Istituto Zooprofilattico Sperimentale Delle Venezie | Lo, F.T.; Diallo, A.A.; Ba, R.O.; Diouf, M.; Diop, A.; Samb, Y.N.; Diouf, M.; Diop, M.; Lo, M.M.; Diouf, M.N.; Zecchin, B.; Tassoni, L.; Fusaro, A.; Pastori, A.; Monne, I.; Terregino, C. |
| [EPI_ISL_2276069](https://platform.epicov.org/epi3/start/EPI_ISL/2276069) | Senegal | 2020-Dec-23 | A/chicken/Senegal/21VIR1084-4/2021 | Istituto Zooprofilattico Sperimentale delle Venezie, EU/OIE/Reference Laboratory and FAO Reference Centre for AI and ND | Istituto Zooprofilattico Sperimentale Delle Venezie | Lo, F.T.; Diallo, A.A.; Ba, R.O.; Diouf, M.; Diop, A.; Samb, Y.N.; Diouf, M.; Diop, M.; Lo, M.M.; Diouf, M.N.; Zecchin, B.; Tassoni, L.; Fusaro, A.; Pastori, A.; Monne, I.; Terregino, C. |
| [EPI_ISL_2276068](https://platform.epicov.org/epi3/start/EPI_ISL/2276068) | Senegal | 2020-Dec-23 | A/chicken/Senegal/21VIR1084-3/2021 | Istituto Zooprofilattico Sperimentale delle Venezie, EU/OIE/Reference Laboratory and FAO Reference Centre for AI and ND | Istituto Zooprofilattico Sperimentale Delle Venezie | Lo, F.T.; Diallo, A.A.; Ba, R.O.; Diouf, M.; Diop, A.; Samb, Y.N.; Diouf, M.; Diop, M.; Lo, M.M.; Diouf, M.N.; Zecchin, B.; Tassoni, L.; Fusaro, A.; Pastori, A.; Monne, I.; Terregino, C. |
| [EPI_ISL_13957823](https://platform.epicov.org/epi3/start/EPI_ISL/13957823) | Mali | 2022-Mar-16 | A/chicken/Mali/T4_180_22VIR6104-7/2022 | Laboratoire Central Vétérinaire | Istituto Zooprofilattico Sperimentale Delle Venezie | DIAKITÉ, A.; DIAKITÉ, M.A.; NIANGALY, F.; SAMAKÉ, K.; DEMBELÉ, C.; TRAORÉ, C.; SIDIBE, C.A.K.; MAIGA, B.M.ditA.; DIALL, M.; OUATTARA, L.; SALLA, A.; NIANG, M.; COULIBALY, D.; KANOUTÉ, B.; Zecchin, B.; Barbierato, G.; Fusaro, A.; Schivo, A.; Salviato, A.; Palumbo, E.; Giussani, E.; Monne, I.; Terregino, C. |
| [EPI_ISL_13957822](https://platform.epicov.org/epi3/start/EPI_ISL/13957822) | Mali | 2022-Mar-16 | A/chicken/Mali/S3-179_22VIR6104-5/2022 | Laboratoire Central Vétérinaire | Istituto Zooprofilattico Sperimentale Delle Venezie | DIAKITÉ, A.; DIAKITÉ, M.A.; NIANGALY, F.; SAMAKÉ, K.; DEMBELÉ, C.; TRAORÉ, C.; SIDIBE, C.A.K.; MAIGA, B.M.ditA.; DIALL, M.; OUATTARA, L.; SALLA, A.; NIANG, M.; COULIBALY, D.; KANOUTÉ, B.; Zecchin, B.; Barbierato, G.; Fusaro, A.; Schivo, A.; Salviato, A.; Palumbo, E.; Giussani, E.; Monne, I.; Terregino, C. |
| [EPI_ISL_13957821](https://platform.epicov.org/epi3/start/EPI_ISL/13957821) | Mali | 2022-Mar-16 | A/chicken/Mali/T2-178_22VIR6104-3/2022 | Laboratoire Central Vétérinaire | Istituto Zooprofilattico Sperimentale Delle Venezie | DIAKITÉ, A.; DIAKITÉ, M.A.; NIANGALY, F.; SAMAKÉ, K.; DEMBELÉ, C.; TRAORÉ, C.; SIDIBE, C.A.K.; MAIGA, B.M.ditA.; DIALL, M.; OUATTARA, L.; SALLA, A.; NIANG, M.; COULIBALY, D.; KANOUTÉ, B.; Zecchin, B.; Barbierato, G.; Fusaro, A.; Schivo, A.; Salviato, A.; Palumbo, E.; Giussani, E.; Monne, I.; Terregino, C. |
| [EPI_ISL_13957819](https://platform.epicov.org/epi3/start/EPI_ISL/13957819) | Mali | 2022-Mar-16 | A/chicken/Mali/T1-177_22VIR6104-1/2022 | Laboratoire Central Vétérinaire | Istituto Zooprofilattico Sperimentale Delle Venezie | DIAKITÉ, A.; DIAKITÉ, M.A.; NIANGALY, F.; SAMAKÉ, K.; DEMBELÉ, C.; TRAORÉ, C.; SIDIBE, C.A.K.; MAIGA, B.M.ditA.; DIALL, M.; OUATTARA, L.; SALLA, A.; NIANG, M.; COULIBALY, D.; KANOUTÉ, B.; Zecchin, B.; Barbierato, G.; Fusaro, A.; Schivo, A.; Salviato, A.; Palumbo, E.; Giussani, E.; Monne, I.; Terregino, C. |
| [EPI_ISL_13048383](https://platform.epicov.org/epi3/start/EPI_ISL/13048383) | Burkina Faso | 2021-Dec-16 | A/chicken/Burkina_Faso/21VIR11911-5/2021 | Laboratoire National d’Elevage | Istituto Zooprofilattico Sperimentale Delle Venezie | LALIDIA-OUOBA, B.; LAMOUNI-ZERBO-OUERMI, H.; Zecchin, B.; Barbierato, G.; OUANDAOGO-SANDAOGO, H.; GUITTI-KINDO, M.; GUIGMA, D.; Barro, N., Palumbo, E.; Giussani, E.; Bortolami, A.; Terregino, C.; Fusaro, A.; Monne, I. |
| [EPI_ISL_14620233](https://platform.epicov.org/epi3/start/EPI_ISL/14620233) | South Africa | 2021-May-19 | A/Buff Orpington chicken/South Africa/21050364/2021 (H5N1) | Western Cape Provincial Veterinary Laboratory | University of Pretoria | Abolnik, Celia |
| [EPI_ISL_14710708](https://platform.epicov.org/epi3/start/EPI_ISL/14710708) | South Africa | 2021-Jun-03 | A/duck/South Africa/21060064/2021 (H5N1) | Western Cape Provincial Veterinary Laboratory | University of Pretoria | Abolnik, Celia |
| [EPI_ISL_14573124](https://platform.epicov.org/epi3/start/EPI_ISL/14573124) | South Africa | 2021-May-13 | A/Egyptian goose/South Africa/21050245A/2021 (H5N1) | Western Cape Provincial Veterinary Laboratory | University of Pretoria | Abolnik, Celia |
| [EPI_ISL_17414661](https://platform.epicov.org/epi3/start/EPI_ISL/17414661) | Nigeria | 2021-Dec-28 | A/duck/Nigeria/740_22VIR3286-28/2021 | National Veterinary Research Institute | Istituto Zooprofilattico Sperimentale Delle Venezie | Meseko, C.; Milani, A.; Inuwa, B.; Chinyere, C.; Shittu, I.; Ahmed, J.; Giussani, E.; Palumbo, E.; Zecchin, B.; Bonfante, F.; Maniero, S.; Fusaro, A.; Gobbo, F.; Terregino, C.; Olasoju, T.; Monne, I.; Muhammad, M. |
| [EPI_ISL_17638495](https://platform.epicov.org/epi3/start/EPI_ISL/17638495) | Niger | 2023-Jan-12 | A/duck/Niger/19-23_23VIR3551-17/2023 | Laboratoire Central de l'Elevage (LABOCEL) | Istituto Zooprofilattico Sperimentale Delle Venezie | Souley, M. M.; Milani, A.; Yaou, B.; Amadou, H.; Haido, A. M.; Issiako, A.; Varotto, M.; Giussani, E.; Palumbo, E.; Zecchin, B.; Fusaro, A. |
| [EPI_ISL_14710814](https://platform.epicov.org/epi3/start/EPI_ISL/14710814) | South Africa | 2021-Jun-03 | A/African fish eagle/South Africa/21060065/2021 (H5N1) | Western Cape Provincial Veterinary Laboratory | University of Pretoria | Abolnik, Celia |
| [EPI_ISL_15839841](https://platform.epicov.org/epi3/start/EPI_ISL/15839841) | South Africa | 2021-Oct-26 | A/African penguin/South Africa/21100423D/2021 | Western Cape Provincial Veterinary Laboratory | University of Pretoria | Abolnik, C. |
| [EPI_ISL_15839840](https://platform.epicov.org/epi3/start/EPI_ISL/15839840) | South Africa | 2021-Oct-26 | A/African penguin/South Africa/21100423C/2021 | Western Cape Provincial Veterinary Laboratory | University of Pretoria | Abolnik, C. |
| [EPI_ISL_15839838](https://platform.epicov.org/epi3/start/EPI_ISL/15839838) | South Africa | 2021-Oct-26 | A/African penguin/South Africa/21100423B/2021 | Western Cape Provincial Veterinary Laboratory | University of Pretoria | Abolnik, C. |
| [EPI_ISL_15839837](https://platform.epicov.org/epi3/start/EPI_ISL/15839837) | South Africa | 2021-Oct-26 | A/African penguin/South Africa/21100423A/2021 | Western Cape Provincial Veterinary Laboratory | University of Pretoria | Abolnik, C. |
| [EPI_ISL_14638909](https://platform.epicov.org/epi3/start/EPI_ISL/14638909) | South Africa | 2021-May-20 | A/African penguin/South Africa/21050383/2021 (H5N1) | Western Cape Provincial Veterinary Laboratory | University of Pretoria | Abolnik, Celia |
| [EPI_ISL_17072111](https://platform.epicov.org/epi3/start/EPI_ISL/17072111) | South Africa | 2022-Nov-07 | A/African penguin/South Africa/702626 AP821/2022 | Assurecloud (Pty) Ltd | University of Pretoria | Abolnik, C. |
| [EPI_ISL_17071943](https://platform.epicov.org/epi3/start/EPI_ISL/17071943) | South Africa | 2022-Nov-04 | A/African penguin/South Africa/702626 DOA410/2022 | Assurecloud (Pty) Ltd | University of Pretoria | Abolnik, C. |
| [EPI_ISL_17071937](https://platform.epicov.org/epi3/start/EPI_ISL/17071937) | South Africa | 2022-Oct-28 | A/African penguin/South Africa/702068 DOA370/2022 | Assurecloud (Pty) Ltd | University of Pretoria | Abolnik, C. |
| [EPI_ISL_17071932](https://platform.epicov.org/epi3/start/EPI_ISL/17071932) | South Africa | 2022-Oct-11 | A/African penguin/South Africa/700164 DOA318/2022 | Assurecloud (Pty) Ltd | University of Pretoria | Abolnik, C. |
| [EPI_ISL_17071927](https://platform.epicov.org/epi3/start/EPI_ISL/17071927) | South Africa | 2022-Sep-28 | A/African penguin/South Africa/698730 DOA308/2022 | Assurecloud (Pty) Ltd | University of Pretoria | Abolnik, C. |
| [EPI_ISL_17069180](https://platform.epicov.org/epi3/start/EPI_ISL/17069180) | South Africa | 2022-Sep-20 | A/African penguin/South Africa/698162 AP446/2022 | Assurecloud (Pty) Ltd | University of Pretoria | Abolnik, C. |
| [EPI_ISL_15853933](https://platform.epicov.org/epi3/start/EPI_ISL/15853933) | South Africa | 2022-Jul-27 | A/African penguin/South Africa/693215 P1-0347/2022 | Assurecloud (Pty) Ltd | University of Pretoria | Abolnik, C. |
| [EPI_ISL_15853932](https://platform.epicov.org/epi3/start/EPI_ISL/15853932) | South Africa | 2022-Jul-27 | A/African penguin/South Africa/693215 P2-0348/2022 | Assurecloud (Pty) Ltd | University of Pretoria | Abolnik, C. |
| [EPI_ISL_15852237](https://platform.epicov.org/epi3/start/EPI_ISL/15852237) | South Africa | 2021-Nov-18 | A/African penguin/South Africa/669895 AP639/2021 | Assurecloud (Pty) Ltd | University of Pretoria | Abolnik, C. |
| [EPI_ISL_16676447](https://platform.epicov.org/epi3/start/EPI_ISL/16676447) | South Africa | 2022-Jun-17 | A/Common tern/South Africa/22060305/2022 | Western Cape Provincial Veterinary Laboratory | University of Pretoria | Abolnik, C. |
| [EPI_ISL_15838583](https://platform.epicov.org/epi3/start/EPI_ISL/15838583) | South Africa | 2021-Oct-16 | A/Northern giant petrel/South Africa/21100283/2021 | Western Cape Provincial Veterinary Laboratory | University of Pretoria | Abolnik, C. |
| [EPI_ISL_2276071](https://platform.epicov.org/epi3/start/EPI_ISL/2276071) | Senegal | 2021-Jan-23 | A/great-white_pelican/Senegal/21-67_21VIR1084-8/2021 | Istituto Zooprofilattico Sperimentale delle Venezie, EU/OIE/Reference Laboratory and FAO Reference Centre for AI and ND | Istituto Zooprofilattico Sperimentale Delle Venezie | Lo, F.T.; Diallo, A.A.; Ba, R.O.; Diouf, M.; Diop, A.; Samb, Y.N.; Diouf, M.; Diop, M.; Lo, M.M.; Diouf, M.N.; Zecchin, B.; Tassoni, L.; Fusaro, A.; Pastori, A.; Monne, I.; Terregino, C. |
| [EPI_ISL_17638505](https://platform.epicov.org/epi3/start/EPI_ISL/17638505) | Niger | 2023-Feb-14 | A/guinea_fowl/Niger/71-23_23VIR3551-38/2023 | Laboratoire Central de l'Elevage (LABOCEL) | Istituto Zooprofilattico Sperimentale Delle Venezie | Souley, M. M.; Milani, A.; Yaou, B.; Amadou, H.; Haido, A. M.; Issiako, A.; Varotto, M.; Giussani, E.; Palumbo, E.; Zecchin, B.; Fusaro, A. |
| [EPI_ISL_15852340](https://platform.epicov.org/epi3/start/EPI_ISL/15852340) | South Africa | 2021-Dec-06 | A/cormorant/South Africa/21120147/2021 | Western Cape Provincial Veterinary Laboratory | University of Pretoria | Abolnik, C. |
| [EPI_ISL_15838197](https://platform.epicov.org/epi3/start/EPI_ISL/15838197) | South Africa | 2021-Oct-13 | A/White breasted cormorant/South Africa/21100215/2021 | Western Cape Provincial Veterinary Laboratory | University of Pretoria | Abolnik, C. |
| [EPI_ISL_15837851](https://platform.epicov.org/epi3/start/EPI_ISL/15837851) | South Africa | 2021-Oct-11 | A/Cape cormorant/South Africa/21100176B/2021 | Western Cape Provincial Veterinary Laboratory | University of Pretoria | Abolnik, C. |
| [EPI_ISL_14973864](https://platform.epicov.org/epi3/start/EPI_ISL/14973864) | South Africa | 2021-Oct-07 | A/Cape cormorant/South Africa/21100109A/2021 (H5N1) | Western Cape Provincial Veterinary Laboratory | University of Pretoria | Abolnik, Celia |
| [EPI_ISL_14973759](https://platform.epicov.org/epi3/start/EPI_ISL/14973759) | South Africa | 2021-Oct-07 | A/Cape cormorant/South Africa/21100109B/2021 (H5N1) | Western Cape Provincial Veterinary Laboratory | University of Pretoria | Abolnik, Celia |
| [EPI_ISL_14973339](https://platform.epicov.org/epi3/start/EPI_ISL/14973339) | South Africa | 2021-Sep-14 | A/Cape cormorant/South Africa/21090250/2021 (H5N1) | Western Cape Provincial Veterinary Laboratory | University of Pretoria | Abolnik, Celia |
| [EPI_ISL_14973297](https://platform.epicov.org/epi3/start/EPI_ISL/14973297) | South Africa | 2021-Sep-13 | A/Cape cormorant/South Africa/21090779D/2021 (H5N1) | Western Cape Provincial Veterinary Laboratory | University of Pretoria | Abolnik, Celia |
| [EPI_ISL_15853701](https://platform.epicov.org/epi3/start/EPI_ISL/15853701) | South Africa | 2022-Mar-23 | A/Cape cormorant/South Africa/681998 DOA059/2022 | Assurecloud (Pty) Ltd | University of Pretoria | Abolnik, C. |
| [EPI_ISL_14871339](https://platform.epicov.org/epi3/start/EPI_ISL/14871339) | Benin | 2021-Sep-01 | A/poultry/Benin/21-A-09-031-O/2021 | na | na | Sanogo,I.N.; Djegui,F.; Dupre,G.; Rubrum,A.; Jeevan,T.; McKenzie,P.; Webby,R.J.; Ducatez,M.F. |
| [EPI_ISL_14871338](https://platform.epicov.org/epi3/start/EPI_ISL/14871338) | Benin | 2021-Sep-01 | A/poultry/Benin/21-A-08-034-O/2021 | na | na | Sanogo,I.N.; Djegui,F.; Dupre,G.; Rubrum,A.; Jeevan,T.; McKenzie,P.; Webby,R.J.; Ducatez,M.F. |
| [EPI_ISL_14871337](https://platform.epicov.org/epi3/start/EPI_ISL/14871337) | Benin | 2021-Sep-01 | A/poultry/Benin/21-A-08-033-O/2021 | na | na | Sanogo,I.N.; Djegui,F.; Dupre,G.; Rubrum,A.; Jeevan,T.; McKenzie,P.; Webby,R.J.; Ducatez,M.F. |
| [EPI_ISL_14871336](https://platform.epicov.org/epi3/start/EPI_ISL/14871336) | Benin | 2021-Aug-01 | A/poultry/Benin/21-A-08-009-O/2021 | na | na | Sanogo,I.N.; Djegui,F.; Dupre,G.; Rubrum,A.; Jeevan,T.; McKenzie,P.; Webby,R.J.; Ducatez,M.F. |
| [EPI_ISL_14871335](https://platform.epicov.org/epi3/start/EPI_ISL/14871335) | Benin | 2021-Sep-01 | A/poultry/Benin/21-A-08-035-O/2021 | na | na | Sanogo,I.N.; Djegui,F.; Dupre,G.; Rubrum,A.; Jeevan,T.; McKenzie,P.; Webby,R.J.; Ducatez,M.F. |
| [EPI_ISL_17414666](https://platform.epicov.org/epi3/start/EPI_ISL/17414666) | Nigeria | 2021-Apr-14 | A/poultry/Nigeria/VRD-21-225_21VIR7423-8/2021 | National Veterinary Research Institute | Istituto Zooprofilattico Sperimentale Delle Venezie | Meseko, C.; Milani, A.; Inuwa, B.; Chinyere, C.; Shittu, I.; Ahmed, J.; Giussani, E.; Palumbo, E.; Zecchin, B.; Bonfante, F.; Maniero, S.; Fusaro, A.; Gobbo, F.; Terregino, C.; Olasoju, T.; Monne, I.; Muhammad, M. |
| [EPI_ISL_17414665](https://platform.epicov.org/epi3/start/EPI_ISL/17414665) | Nigeria | 2021-Apr-12 | A/poultry/Nigeria/VRD-21-219_21VIR7423-6/2021 | National Veterinary Research Institute | Istituto Zooprofilattico Sperimentale Delle Venezie | Meseko, C.; Milani, A.; Inuwa, B.; Chinyere, C.; Shittu, I.; Ahmed, J.; Giussani, E.; Palumbo, E.; Zecchin, B.; Bonfante, F.; Maniero, S.; Fusaro, A.; Gobbo, F.; Terregino, C.; Olasoju, T.; Monne, I.; Muhammad, M. |
| [EPI_ISL_17414664](https://platform.epicov.org/epi3/start/EPI_ISL/17414664) | Nigeria | 2021-Mar-10 | A/poultry/Nigeria/VRD-21-135_21VIR7423-18/2021 | National Veterinary Research Institute | Istituto Zooprofilattico Sperimentale Delle Venezie | Meseko, C.; Milani, A.; Inuwa, B.; Chinyere, C.; Shittu, I.; Ahmed, J.; Giussani, E.; Palumbo, E.; Zecchin, B.; Bonfante, F.; Maniero, S.; Fusaro, A.; Gobbo, F.; Terregino, C.; Olasoju, T.; Monne, I.; Muhammad, M. |
| [EPI_ISL_15852757](https://platform.epicov.org/epi3/start/EPI_ISL/15852757) | South Africa | 2022-Jan-28 | A/chicken/South Africa/22010387/2022 | Western Cape Provincial Veterinary Laboratory | University of Pretoria | Abolnik, C. |
| [EPI_ISL_15852181](https://platform.epicov.org/epi3/start/EPI_ISL/15852181) | South Africa | 2021-Oct-28 | A/chicken/South Africa/21100506/2021 | Western Cape Provincial Veterinary Laboratory | University of Pretoria | Abolnik, C. |
| [EPI_ISL_15837660](https://platform.epicov.org/epi3/start/EPI_ISL/15837660) | South Africa | 2021-Oct-10 | A/chicken/South Africa/21100111/2021 | Western Cape Provincial Veterinary Laboratory | University of Pretoria | Abolnik, C. |
| [EPI_ISL_15837655](https://platform.epicov.org/epi3/start/EPI_ISL/15837655) | South Africa | 2022-Oct-10 | A/chicken/South Africa/21080238/2021 | Western Cape Provincial Veterinary Laboratory | University of Pretoria | Abolnik, C |
| [EPI_ISL_14918800](https://platform.epicov.org/epi3/start/EPI_ISL/14918800) | South Africa | 2021-Jun-25 | A/chicken/South Africa/21060469/2021 (H5N1) | Western Cape Provincial Veterinary Laboratory | University of Pretoria | Abolnik, Celia |
| [EPI_ISL_14918605](https://platform.epicov.org/epi3/start/EPI_ISL/14918605) | South Africa | 2021-Jun-24 | A/chicken/South Africa/21060435/2021 (H5N1) | Western Cape Provincial Veterinary Laboratory | University of Pretoria | Abolnik, Celia |
| [EPI_ISL_14711165](https://platform.epicov.org/epi3/start/EPI_ISL/14711165) | South Africa | 2021-Jun-14 | A/chicken/South Africa/21060265/2021 (H5N1) | Western Cape Provincial Veterinary Laboratory | University of Pretoria | Abolnik, Celia |
| [EPI_ISL_14619024](https://platform.epicov.org/epi3/start/EPI_ISL/14619024) | South Africa | 2021-May-18 | A/chicken/South Africa/21050299/2021 (H5N1) | Western Cape Provincial Veterinary Laboratory | University of Pretoria | Abolnik, Celia |
| [EPI_ISL_14619020](https://platform.epicov.org/epi3/start/EPI_ISL/14619020) | South Africa | 2021-May-17 | A/chicken/South Africa/21050293/2021 (H5N1) | Western Cape Provincial Veterinary Laboratory | University of Pretoria | Abolnik, Celia |
| [EPI_ISL_14572927](https://platform.epicov.org/epi3/start/EPI_ISL/14572927) | South Africa | 2021-May-10 | A/chicken/South Africa/21050021/2021 (H5N1) | Western Cape Provincial Veterinary Laboratory | University of Pretoria | Abolnik, Celia |
| [EPI_ISL_14572124](https://platform.epicov.org/epi3/start/EPI_ISL/14572124) | South Africa | 2021-May-08 | A/chicken/South Africa/21050125/2021 (H5N1) | Western Cape Provincial Veterinary Laboratory | University of Pretoria | Abolnik, Celia |
| [EPI_ISL_14571452](https://platform.epicov.org/epi3/start/EPI_ISL/14571452) | South Africa | 2021-May-07 | A/chicken/South Africa/21050119/2021 (H5N1) | Western Cape Provincial Veterinary Laboratory | University of Pretoria | Abolnik, Celia |
| [EPI_ISL_14543005](https://platform.epicov.org/epi3/start/EPI_ISL/14543005) | South Africa | 2021-May-07 | A/chicken/South Africa/21050118/2021 (H5N1) | Western Cape Provincial Veterinary Laboratory | University of Pretoria | Abolnik, Celia |
| [EPI_ISL_14543004](https://platform.epicov.org/epi3/start/EPI_ISL/14543004) | South Africa | 2021-May-06 | A/chicken/South Africa/21050090/2021 (H5N1) | Western Cape Provincial Veterinary Laboratory | University of Pretoria | Abolnik, Celia |
| [EPI_ISL_15852846](https://platform.epicov.org/epi3/start/EPI_ISL/15852846) | South Africa | 2022-Feb-07 | A/chicken/South Africa/QF22465/2022 | SMT Veterinary Laboratory | University of Pretoria | Abolnik, C. |
| [EPI_ISL_15852844](https://platform.epicov.org/epi3/start/EPI_ISL/15852844) | South Africa | 2022-Jan-28 | A/chicken/South Africa/QF22304/2022 | SMT Veterinary Laboratory | University of Pretoria | Abolnik, C. |
| [EPI_ISL_15852626](https://platform.epicov.org/epi3/start/EPI_ISL/15852626) | South Africa | 2022-Jan-24 | A/chicken/South Africa/DW2201/2022 | SMT Veterinary Laboratory | University of Pretoria | Abolnik, C. |
| [EPI_ISL_15852625](https://platform.epicov.org/epi3/start/EPI_ISL/15852625) | South Africa | 2022-Jan-24 | A/chicken/South Africa/JB2201/2022 | SMT Veterinary Laboratory | University of Pretoria | Abolnik, C. |
| [EPI_ISL_15853438](https://platform.epicov.org/epi3/start/EPI_ISL/15853438) | South Africa | 2022-Feb-22 | A/chicken/South Africa/PRL118/2022 | Faculty of Veterinary Science, University of Pretoria | University of Pretoria | Abolnik, C. |
| [EPI_ISL_15852179](https://platform.epicov.org/epi3/start/EPI_ISL/15852179) | South Africa | 2021-Oct-27 | A/chicken/South Africa/UP928/2021 | Faculty of Veterinary Science, University of Pretoria | University of Pretoria | Abolnik, C. |
| [EPI_ISL_14934607](https://platform.epicov.org/epi3/start/EPI_ISL/14934607) | South Africa | 2021-Aug-27 | A/chicken/South Africa/MAB/2021 (H5N1) | Faculty of Veterinary Science, University of Pretoria | University of Pretoria | Abolnik, Celia |
| [EPI_ISL_14573053](https://platform.epicov.org/epi3/start/EPI_ISL/14573053) | South Africa | 2021-May-10 | A/chicken/South Africa/UP481/2021 (H5N1) | Faculty of Veterinary Science, University of Pretoria | University of Pretoria | Abolnik, Celia |
| [EPI_ISL_14542831](https://platform.epicov.org/epi3/start/EPI_ISL/14542831) | South Africa | 2021-May-01 | A/chicken/South Africa/UP01/2021 (H5N1) | Faculty of Veterinary Science, University of Pretoria | University of Pretoria | Abolnik, Celia |
| [EPI_ISL_15853935](https://platform.epicov.org/epi3/start/EPI_ISL/15853935) | South Africa | 2022-Sep-18 | A/chicken/South Africa/697683/2022 | Assurecloud (Pty) Ltd | University of Pretoria | Abolnik, C. |
| [EPI_ISL_14934884](https://platform.epicov.org/epi3/start/EPI_ISL/14934884) | South Africa | 2021-Sep-08 | A/chicken/South Africa/412381/2021 (H5N1) | Assurecloud (Pty) Ltd | University of Pretoria | Abolnik, Celia |
| [EPI_ISL_14934883](https://platform.epicov.org/epi3/start/EPI_ISL/14934883) | South Africa | 2021-Sep-07 | A/chicken/South Africa/412380/2021 (H5N1) | Assurecloud (Pty) Ltd | University of Pretoria | Abolnik, Celia |
| [EPI_ISL_14934479](https://platform.epicov.org/epi3/start/EPI_ISL/14934479) | South Africa | 2021-Aug-18 | A/chicken/South Africa/424469/2021 (H5N1) | Assurecloud (Pty) Ltd | University of Pretoria | Abolnik, Celia |
| [EPI_ISL_14934383](https://platform.epicov.org/epi3/start/EPI_ISL/14934383) | South Africa | 2021-Aug-16 | A/chicken/South Africa/10766/2021 (H5N1) | Assurecloud (Pty) Ltd | University of Pretoria | Abolnik, Celia |
| [EPI_ISL_14934318](https://platform.epicov.org/epi3/start/EPI_ISL/14934318) | South Africa | 2021-Aug-09 | A/chicken/South Africa/411262/2021 (H5N1) | Assurecloud (Pty) Ltd | University of Pretoria | Abolnik, Celia |
| [EPI_ISL_14934317](https://platform.epicov.org/epi3/start/EPI_ISL/14934317) | South Africa | 2021-Jul-30 | A/chicken/South Africa/412374/2021 (H5N1) | Assurecloud (Pty) Ltd | University of Pretoria | Abolnik, Celia |
| [EPI_ISL_14933916](https://platform.epicov.org/epi3/start/EPI_ISL/14933916) | South Africa | 2021-Jul-29 | A/chicken/South Africa/411258/2021 (H5N1) | Assurecloud (Pty) Ltd | University of Pretoria | Abolnik, Celia |
| [EPI_ISL_14933725](https://platform.epicov.org/epi3/start/EPI_ISL/14933725) | South Africa | 2021-Jul-28 | A/chicken/South Africa/412369/2021 (H5N1) | Assurecloud (Pty) Ltd | University of Pretoria | Abolnik, Celia |
| [EPI_ISL_14933723](https://platform.epicov.org/epi3/start/EPI_ISL/14933723) | South Africa | 2021-Jul-28 | A/chicken/South Africa/412372/2021 (H5N1) | Assurecloud (Pty) Ltd | University of Pretoria | Abolnik, Celia |
| [EPI_ISL_14933721](https://platform.epicov.org/epi3/start/EPI_ISL/14933721) | South Africa | 2021-Jul-27 | A/chicken/South Africa/33081/2021 (H5N1) | Assurecloud (Pty) Ltd | University of Pretoria | Abolnik, Celia |
| [EPI_ISL_14933695](https://platform.epicov.org/epi3/start/EPI_ISL/14933695) | South Africa | 2021-Jul-26 | A/chicken/South Africa/412364/2021 (H5N1) | Assurecloud (Pty) Ltd | University of Pretoria | Abolnik, Celia |
| [EPI_ISL_14933117](https://platform.epicov.org/epi3/start/EPI_ISL/14933117) | South Africa | 2021-Jul-26 | A/chicken/South Africa/33069/2021 (H5N1) | Assurecloud (Pty) Ltd | University of Pretoria | Abolniik, Celia |
| [EPI_ISL_14933096](https://platform.epicov.org/epi3/start/EPI_ISL/14933096) | South Africa | 2021-Jul-25 | A/chicken/South Africa/33071/2021 (H5N1) | Assurecloud (Pty) Ltd | University of Pretoria | Abolnik, Celia |
| [EPI_ISL_14933095](https://platform.epicov.org/epi3/start/EPI_ISL/14933095) | South Africa | 2021-Jul-23 | A/chicken/South Africa/411554/2021 (H5N1) | Assurecloud (Pty) Ltd | University of Pretoria | Abolnik, Celia |
| [EPI_ISL_14932914](https://platform.epicov.org/epi3/start/EPI_ISL/14932914) | South Africa | 2021-Jul-20 | A/chicken/South Africa/411542/2021 (H5N1) | Assurecloud (Pty) Ltd | University of Pretoria | Abolnik, Celia |
| [EPI_ISL_14918903](https://platform.epicov.org/epi3/start/EPI_ISL/14918903) | South Africa | 2021-Jul-19 | A/chicken/South Africa/411255/2021 (H5N1) | Assurecloud (Pty) Ltd | University of Pretoria | Abolnik, Celia |
| [EPI_ISL_14918892](https://platform.epicov.org/epi3/start/EPI_ISL/14918892) | South Africa | 2021-Jun-30 | A/chicken/South Africa/683320/2021 (H5N1) | Assurecloud (Pty) Ltd | University of Pretoria | Abolnik, Celia |
| [EPI_ISL_14918217](https://platform.epicov.org/epi3/start/EPI_ISL/14918217) | South Africa | 2021-Jun-20 | A/chicken/South Africa/697352/2021 (H5N1) | Assurecloud (Pty) Ltd | University of Pretoria | Abolnik, Celia |
| [EPI_ISL_14711044](https://platform.epicov.org/epi3/start/EPI_ISL/14711044) | South Africa | 2021-Jun-08 | A/chicken/South Africa/695344/2021 (H5N1) | Assurecloud (Pty) Ltd | University of Pretoria | Abolnik, Celia |
| [EPI_ISL_14710239](https://platform.epicov.org/epi3/start/EPI_ISL/14710239) | South Africa | 2021-May-31 | A/chicken/South Africa/693965/2021 (H5N1) | Assurecloud (Pty) Ltd | University of Pretoria | Abolnik, Celia |
| [EPI_ISL_14645717](https://platform.epicov.org/epi3/start/EPI_ISL/14645717) | South Africa | 2021-May-27 | A/chicken/South Africa/693781/2021 (H5N1) | Assurecloud (Pty) Ltd | University of Pretoria | Abolnik, Celia |
| [EPI_ISL_14644201](https://platform.epicov.org/epi3/start/EPI_ISL/14644201) | South Africa | 2021-May-23 | A/chicken/South Africa/693331/2021 (H5N1) | Assurecloud (Pty) Ltd | University of Pretoria | Abolnik, Celia |
| [EPI_ISL_14643779](https://platform.epicov.org/epi3/start/EPI_ISL/14643779) | South Africa | 2021-May-20 | A/chicken/South Africa/692881/2021 (H5N1) | Assurecloud (Pty) Ltd | University of Pretoria | Abolnik, Celia |
| [EPI_ISL_14620174](https://platform.epicov.org/epi3/start/EPI_ISL/14620174) | South Africa | 2021-May-18 | A/chicken/South Africa/692329/2021 (H5N1) | Assurecloud (Pty) Ltd | University of Pretoria | Abolnik, Celia |
| [EPI_ISL_14619775](https://platform.epicov.org/epi3/start/EPI_ISL/14619775) | South Africa | 2021-May-18 | A/chicken/South Africa/692328/2021 (H5N1) | Assurecloud (Pty) Ltd | University of Pretoria | Abolnik, Celia |
| [EPI_ISL_14573038](https://platform.epicov.org/epi3/start/EPI_ISL/14573038) | South Africa | 2021-May-10 | A/chicken/South Africa/690813/2021 (H5N1) | Assurecloud (Pty) Ltd | University of Pretoria | Abolnik, Celia |
| [EPI_ISL_14573036](https://platform.epicov.org/epi3/start/EPI_ISL/14573036) | South Africa | 2021-May-10 | A/chicken/South Africa/690841/2021 (H5N1) | Assurecloud (Pty) Ltd | University of Pretoria | Abolnik, Celia |
| [EPI_ISL_14542586](https://platform.epicov.org/epi3/start/EPI_ISL/14542586) | South Africa | 2021-Apr-26 | A/chicken/South Africa/690109/2021 (mixed) | Assurecloud (Pty) Ltd | University of Pretoria | Abolnik, Celia |
| [EPI_ISL_14542530](https://platform.epicov.org/epi3/start/EPI_ISL/14542530) | South Africa | 2021-Apr-19 | A/chicken/South Africa/26700/2021 (H5N1) | Assurecloud (Pty) Ltd | University of Pretoria | Abolnik, Celia |
| [EPI_ISL_14542488](https://platform.epicov.org/epi3/start/EPI_ISL/14542488) | South Africa | 2021-Apr-09 | A/chicken/South Africa/26683/2021 (H5N1) | Assurecloud (Pty) Ltd | University of Pretoria | Abolnik, Celia |
| [EPI_ISL_15838771](https://platform.epicov.org/epi3/start/EPI_ISL/15838771) | South Africa | 2021-Oct-19 | A/chicken/South Africa/236744/2021 | Assurecloud (Pty) Ltd | University of Pretoria | Abolnik, C. |
| [EPI_ISL_14573033](https://platform.epicov.org/epi3/start/EPI_ISL/14573033) | South Africa | 2021-May-10 | A/chicken/South Africa/690575/2021 (H5N1) | Assurecloud (Pty) Ltd | University of Pretoria | Abolnik, Celia |
| [EPI_ISL_12045320](https://platform.epicov.org/epi3/start/EPI_ISL/12045320) | Botswana | 2021-Sep-08 | A/chicken/Botswana/2248-A/2021 | Botswana National Veterinary Laboratory | Animal and Plant Health Agency (APHA) | na |
| [EPI_ISL_12045319](https://platform.epicov.org/epi3/start/EPI_ISL/12045319) | Botswana | 2021-Aug-18 | A/chicken/Botswana/2163-B/2021 | Botswana National Veterinary Laboratory | Animal and Plant Health Agency (APHA) | na |
| [EPI_ISL_16811183](https://platform.epicov.org/epi3/start/EPI_ISL/16811183) | United Kingdom | 2023-Jan-08 | A/chicken/England/005094/2023 | Animal and Plant Health Agency (APHA) | Animal and Plant Health Agency (APHA) | na |
| [EPI_ISL_15925878](https://platform.epicov.org/epi3/start/EPI_ISL/15925878) | Netherlands | 2022-Nov-01 | A/Common Teal/Netherlands/5/2022 | Erasmus Medical Center | Erasmus Medical Center | na |
| [EPI_ISL_16809297](https://platform.epicov.org/epi3/start/EPI_ISL/16809297) | Belgium | 2023-Jan-19 | A/Gallus_gallus/Belgium/00548_0001/2023 | Sciensano - Animal Infectious Diseases | Sciensano, Department of Animal Infectious Diseases | Van Borm, Steven; Roupie, Virginie; Hostyn, Pierre; Lambrecht, Benedicte; Steensels, Mieke |
| [EPI_ISL_16833158](https://platform.epicov.org/epi3/start/EPI_ISL/16833158) | Luxembourg | 2023-Jan-25 | A/chicken/Luxembourg/23023602/2023 | Laboratoire de médecine vétérinaire de l'Etat | Luxembourg Institute of Health | Chantal Snoeck, Aurélie Sausy |
| [EPI_ISL_8814146](https://platform.epicov.org/epi3/start/EPI_ISL/8814146) | United Kingdom | 2021-Nov-06 | A/turkey/England/055251/2021 | Animal and Plant Health Agency (APHA) | Animal and Plant Health Agency (APHA) | na |
| [EPI_ISL_13370702](https://platform.epicov.org/epi3/start/EPI_ISL/13370702) | United Kingdom | 2022-Jan-27 | A/mallard_duck/England/388009/2022 | Animal and Plant Health Agency (APHA) | Animal and Plant Health Agency (APHA) | na |
| [EPI_ISL_13370703](https://platform.epicov.org/epi3/start/EPI_ISL/13370703) | United Kingdom | 2022-Feb-03 | A/domestic_duck/England/012247/2022 | Animal and Plant Health Agency (APHA) | Animal and Plant Health Agency (APHA) | na |
| [EPI_ISL_13370704](https://platform.epicov.org/epi3/start/EPI_ISL/13370704) | United Kingdom | 2022-Feb-12 | A/chicken/England/014330/2022 | Animal and Plant Health Agency (APHA) | Animal and Plant Health Agency (APHA) | na |
| [EPI_ISL_13370705](https://platform.epicov.org/epi3/start/EPI_ISL/13370705) | United Kingdom | 2022-Feb-20 | A/pheasant/Wales/016441/2022 | Animal and Plant Health Agency (APHA) | Animal and Plant Health Agency (APHA) | na |
| [EPI_ISL_13370706](https://platform.epicov.org/epi3/start/EPI_ISL/13370706) | United Kingdom | 2022-Feb-20 | A/pheasant/Wales/016303/2022 | Animal and Plant Health Agency (APHA) | Animal and Plant Health Agency (APHA) | na |
| [EPI_ISL_13370707](https://platform.epicov.org/epi3/start/EPI_ISL/13370707) | United Kingdom | 2022-Feb-21 | A/domestic_duck/England/017166/2022 | Animal and Plant Health Agency (APHA) | Animal and Plant Health Agency (APHA) | na |
| [EPI_ISL_13370559](https://platform.epicov.org/epi3/start/EPI_ISL/13370559) | United Kingdom | 2021-Dec-09 | A/turkey/England/068583/2021 | Animal and Plant Health Agency (APHA) | Animal and Plant Health Agency (APHA) | na |
| [EPI_ISL_1224949](https://platform.epicov.org/epi3/start/EPI_ISL/1224949) | Netherlands | 2021-Feb-04 | A/mute swan/Netherlands/21022898-002/2021 | Wageningen Bioveterinary Research | Wageningen Bioveterinary Research | Beerens, Nancy; Harders, Frank; Pritz-Verschuren, Sylvia; Roose, Marit; Germeraad, Evelien; Engelsma, Marc; Bossers, Alex; Heutink, Rene |
| [EPI_ISL_1224989](https://platform.epicov.org/epi3/start/EPI_ISL/1224989) | Netherlands | 2021-Feb-01 | A/common buzzard/Netherlands/21022834-002/2021 | Wageningen Bioveterinary Research | Wageningen Bioveterinary Research | Beerens, Nancy; Harders, Frank; Pritz-Verschuren, Sylvia; Roose, Marit; Germeraad, Evelien; Engelsma, Marc; Bossers, Alex; Heutink, Rene |
| [EPI_ISL_1225077](https://platform.epicov.org/epi3/start/EPI_ISL/1225077) | Netherlands | 2021-Feb-21 | A/european herring gull/Netherlands/21023937-002/2021 | Wageningen Bioveterinary Research | Wageningen Bioveterinary Research | Beerens, Nancy; Harders, Frank; Pritz-Verschuren, Sylvia; Roose, Marit; Germeraad, Evelien; Engelsma, Marc; Bossers, Alex; Heutink, Rene |
| [EPI_ISL_1225079](https://platform.epicov.org/epi3/start/EPI_ISL/1225079) | Netherlands | 2021-Feb-15 | A/eurasian curlew/Netherlands/21024069-002/2021 | Wageningen Bioveterinary Research | Wageningen Bioveterinary Research | Beerens, Nancy; Harders, Frank; Pritz-Verschuren, Sylvia; Roose, Marit; Germeraad, Evelien; Engelsma, Marc; Bossers, Alex; Heutink, Rene |
| [EPI_ISL_654958](https://platform.epicov.org/epi3/start/EPI_ISL/654958) | Italy | 2020-Nov-14 | A/mallard/Italy/20VIR7139-73/2020 | Istituto Zooprofilattico Sperimentale delle Venezie, EU/OIE/Reference Laboratory and FAO Reference Centre for AI and ND | Istituto Zooprofilattico Sperimentale Delle Venezie | Zecchin, B.; Fusaro, A.; Pastori, A.; Milani, A.; Salviato, A.; Schivo, A.;Monne, I.; Terregino, C. |
| [EPI_ISL_683592](https://platform.epicov.org/epi3/start/EPI_ISL/683592) | Italy | 2020-Nov-21 | A/Eurasian_wigeon/Italy/20VIR7301-206/2020 | Istituto Zooprofilattico Sperimentale delle Venezie, EU/OIE/Reference Laboratory and FAO Reference Centre for AI and ND | Istituto Zooprofilattico Sperimentale Delle Venezie | Zecchin, B.; Fusaro, A.; Pastori, A.; Milani, A.; Salviato, A.; Schivo, A.; Monne, I.; Terregino, C. |
| [EPI_ISL_683593](https://platform.epicov.org/epi3/start/EPI_ISL/683593) | Italy | 2020-Nov-14 | A/Eurasian_wigeon/Italy/20VIR7139-121/2020 | Istituto Zooprofilattico Sperimentale delle Venezie, EU/OIE/Reference Laboratory and FAO Reference Centre for AI and ND | Istituto Zooprofilattico Sperimentale Delle Venezie | Zecchin, B.; Fusaro, A.; Pastori, A.; Milani, A.; Salviato, A.; Schivo, A.; Monne, I.; Terregino, C. |
| [EPI_ISL_1139101](https://platform.epicov.org/epi3/start/EPI_ISL/1139101) | Netherlands | 2020-Nov-09 | A/swan/Netherlands/20017605-002/2020 | Wageningen Bioveterinary Research | Wageningen Bioveterinary Research | Beerens, Nancy; Harders, Frank; Pritz-Verschuren, Sylvia; Roose, Marit; Germeraad, Evelien; Engelsma, Marc; Bossers, Alex; Heutink, Rene |
| [EPI_ISL_1139102](https://platform.epicov.org/epi3/start/EPI_ISL/1139102) | Netherlands | 2020-Nov-10 | A/swan/Netherlands/20017772-002/2020 | Wageningen Bioveterinary Research | Wageningen Bioveterinary Research | Beerens, Nancy; Harders, Frank; Pritz-Verschuren, Sylvia; Roose, Marit; Germeraad, Evelien; Engelsma, Marc; Bossers, Alex; Heutink, Rene |
| [EPI_ISL_1139103](https://platform.epicov.org/epi3/start/EPI_ISL/1139103) | Netherlands | 2020-Nov-01 | A/peregrine falcon/Netherlands/20017773-002/2020 | Wageningen Bioveterinary Research | Wageningen Bioveterinary Research | Beerens, Nancy; Harders, Frank; Pritz-Verschuren, Sylvia; Roose, Marit; Germeraad, Evelien; Engelsma, Marc; Bossers, Alex; Heutink, Rene |
| [EPI_ISL_1123360](https://platform.epicov.org/epi3/start/EPI_ISL/1123360) | United Kingdom | 2020-Dec-01 | A/mute_swan/England/234135/2020 | Animal and Plant Health Agency (APHA) | Animal and Plant Health Agency (APHA) | na |
| [EPI_ISL_1123361](https://platform.epicov.org/epi3/start/EPI_ISL/1123361) | United Kingdom | 2021-Feb-10 | A/pheasant/Scotland/000348/2021 | Animal and Plant Health Agency (APHA) | Animal and Plant Health Agency (APHA) | na |
| [EPI_ISL_1139014](https://platform.epicov.org/epi3/start/EPI_ISL/1139014) | Netherlands | 2020-Oct-27 | A/barnacle goose/Netherlands/20016511-002/2020 | Wageningen Bioveterinary Research | Wageningen Bioveterinary Research | Beerens, Nancy; Harders, Frank; Pritz-Verschuren, Sylvia; Roose, Marit; Germeraad, Evelien; Engelsma, Marc; Bossers, Alex; Heutink, Rene |
| [EPI_ISL_1122425](https://platform.epicov.org/epi3/start/EPI_ISL/1122425) | United Kingdom | 2020-Dec-15 | A/chicken/England/043315/2020 | Animal and Plant Health Agency (APHA) | Animal and Plant Health Agency (APHA) | na |
| [EPI_ISL_1123263](https://platform.epicov.org/epi3/start/EPI_ISL/1123263) | United Kingdom | 2020-Dec-16 | A/chicken/Scotland/043405/2020 | Animal and Plant Health Agency (APHA) | Animal and Plant Health Agency (APHA) | na |
| [EPI_ISL_1123350](https://platform.epicov.org/epi3/start/EPI_ISL/1123350) | United Kingdom | 2020-Dec-17 | A/chicken/England/043683/2020 | Animal and Plant Health Agency (APHA) | Animal and Plant Health Agency (APHA) | na |
| [EPI_ISL_1139101](https://platform.epicov.org/epi3/start/EPI_ISL/1139101) | Netherlands | 2020-Nov-09 | A/swan/Netherlands/20017605-002/2020 | Wageningen Bioveterinary Research | Wageningen Bioveterinary Research | Beerens, Nancy; Harders, Frank; Pritz-Verschuren, Sylvia; Roose, Marit; Germeraad, Evelien; Engelsma, Marc; Bossers, Alex; Heutink, Rene |
| [EPI_ISL_1139102](https://platform.epicov.org/epi3/start/EPI_ISL/1139102) | Netherlands | 2020-Nov-10 | A/swan/Netherlands/20017772-002/2020 | Wageningen Bioveterinary Research | Wageningen Bioveterinary Research | Beerens, Nancy; Harders, Frank; Pritz-Verschuren, Sylvia; Roose, Marit; Germeraad, Evelien; Engelsma, Marc; Bossers, Alex; Heutink, Rene |
| [EPI_ISL_1665250](https://platform.epicov.org/epi3/start/EPI_ISL/1665250) | Slovakia | 2021-Jan-22 | A/chicken/Slovakia/Pah10_21VIR1086-5/2021 | Istituto Zooprofilattico Sperimentale delle Venezie, EU/OIE/Reference Laboratory and FAO Reference Centre for AI and ND | Istituto Zooprofilattico Sperimentale Delle Venezie | Dirb?kov?, Z.; Tin?k, M.; Zecchin, B.; Fusaro, A.; Pastori, A.; Schivo, A.; Salviato, A.; Monne, I.; Terregino, C. |
| [EPI_ISL_1665251](https://platform.epicov.org/epi3/start/EPI_ISL/1665251) | Italy | 2020-Nov-28 | A/common_teal/Italy/20VIR7439-191/2020 | Istituto Zooprofilattico Sperimentale delle Venezie, EU/OIE/Reference Laboratory and FAO Reference Centre for AI and ND | Istituto Zooprofilattico Sperimentale Delle Venezie | Zecchin, B.; Fusaro, A.; Pastori, A.; Schivo, A.; Salviato, A.; Monne, I.; Terregino, C. |
